# Supplementary material for: Different modes of barrel opening suggest a complex pathway of ligand binding in human gastrotropin
Source: PLoS One. 2019 May 10;14(5):e0216142. doi: 10.1371/journal.pone.0216142 (PMC6510414; doi:10.1371/journal.pone.0216142)
Supplement: S1 Fig — (A) Apo MUMO ensembles by amino acids (B) Apo MUMO ensembles as correlation plots (C) Holo MUMO ensembles by amino acids (D)Holo MUMO ensembles as correlation plots (E) Unrestrained ensembles by amino acids (F) Unrestrained ensembles as correlation plots The PDB ensembles are depicted in all panels as references. All the plots were generated by the CoNSEnsX webserver. (PDF) [file pone.0216142.s001.pdf]

A

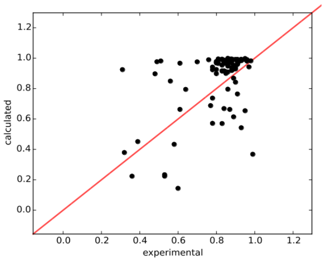

1O1U PDB 313

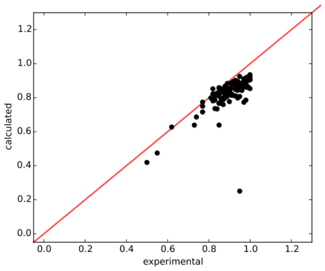

Apo 6 ns 283 K

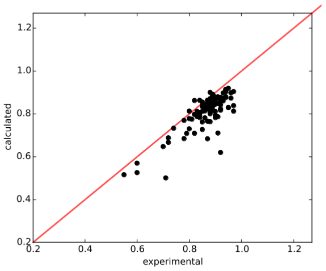

Apo 6 ns 291 K

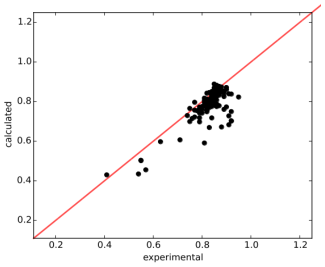

Apo 6 ns 298 K

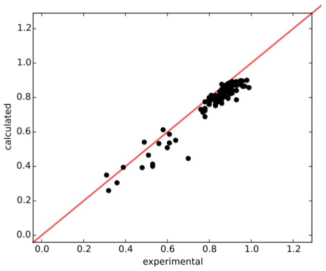

Apo 6 ns 313 K

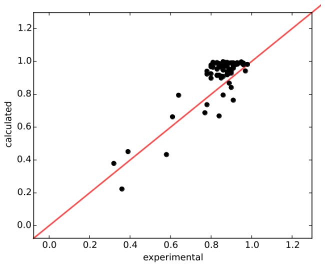

1O1U PDB 313 corrected

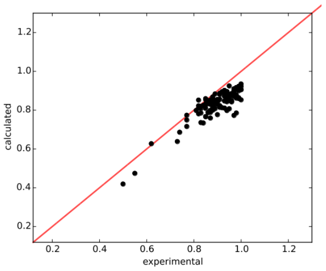

Apo 6 ns 283 K corrected

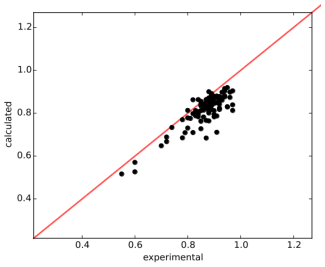

Apo 6 ns 291 K corrected

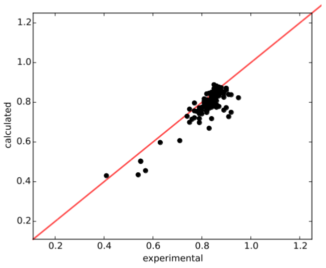

Apo 6 ns 298 K corrected

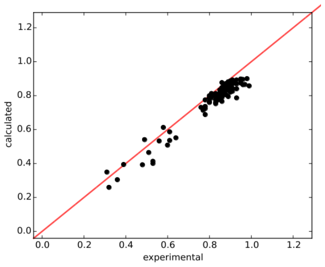

Apo 6 ns 313 K corrected

B	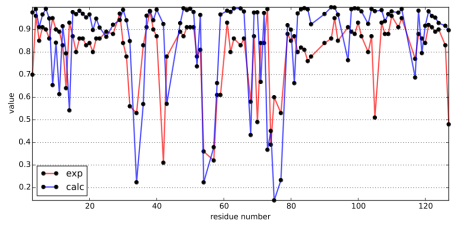	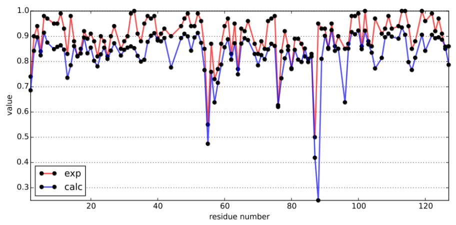
	1O1U PDB 313	Apo 6 ns 283 K
	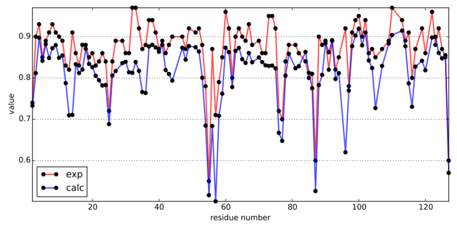	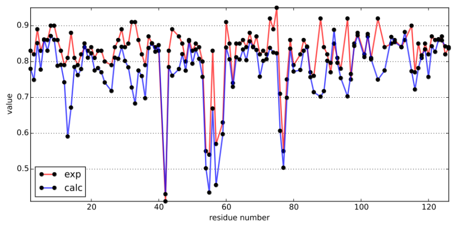
	Apo 6 ns 291 K	Apo 6 ns 298 K
	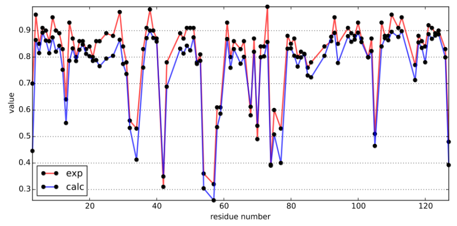	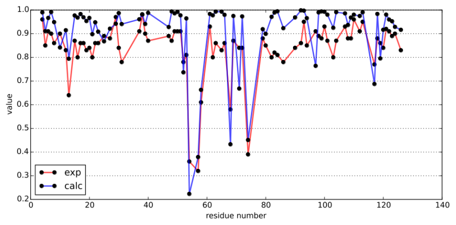
	Apo 6 ns 313 K	1O1U PDB 313 corrected
	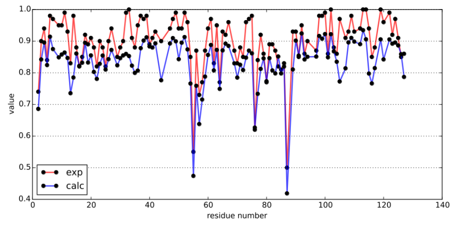	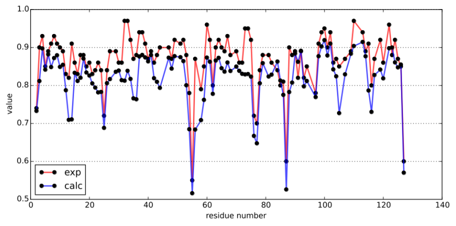
	Apo 6 ns 283 K corrected	Apo 6 ns 291 K corrected
	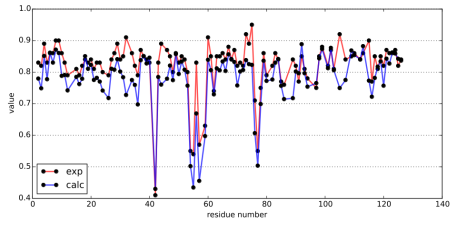	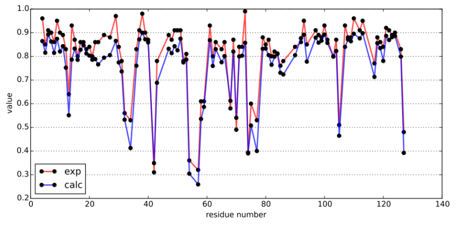
	Apo 6 ns 298 K corrected	Apo 6 ns 313 K corrected

C

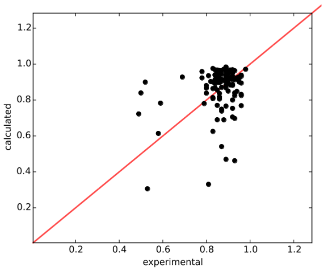

2MM3 PDB 298

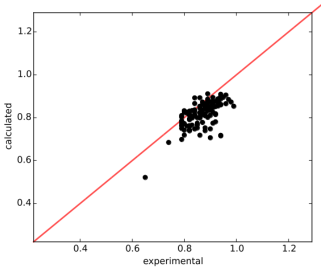

Holo 6 ns 283 K

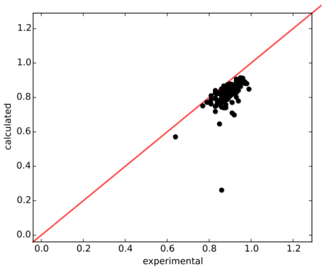

Holo 6 ns 291 K

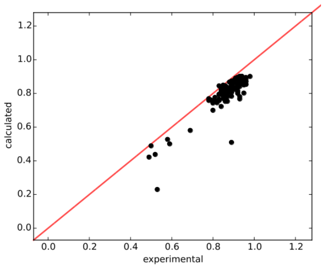

Holo 6 ns 298 K

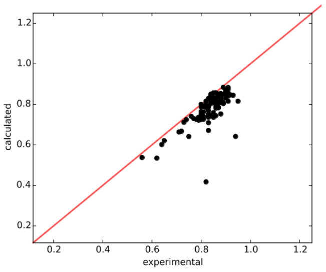

Holo 6 ns 313 K

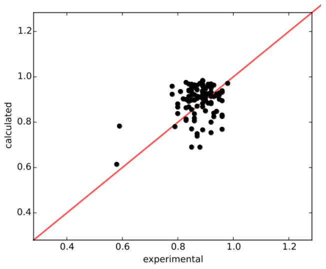

2MM3 PDB 298 corrected

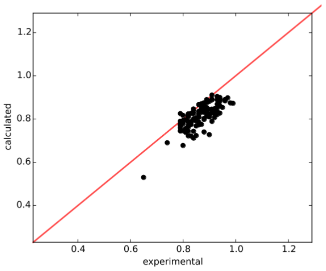

Holo 6 ns 283 K corrected

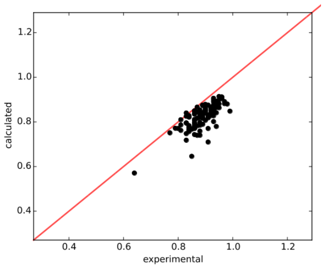

Holo 6 ns 291 K corrected

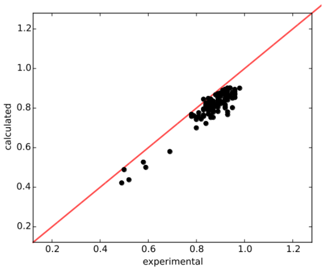

Holo 6 ns 298 K corrected

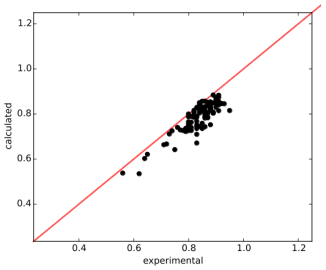

Holo 6 ns 313 K corrected

D	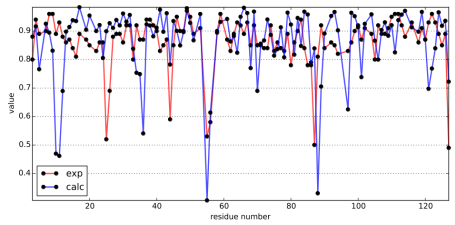	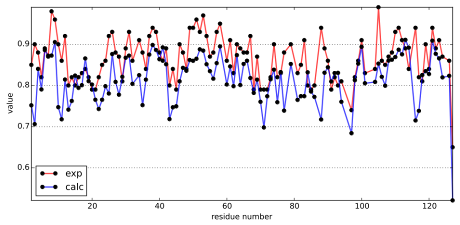
	2MM3 PDB 298	Holo 6 ns 283 K
	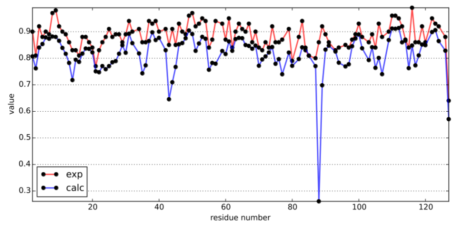	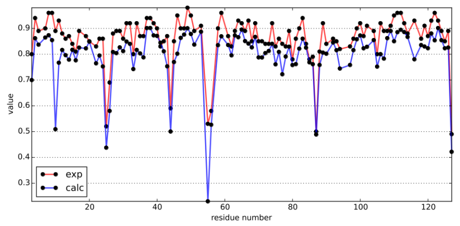
	Holo 6 ns 291 K	Holo 6 ns 298 K
	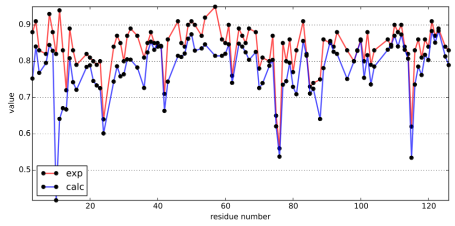	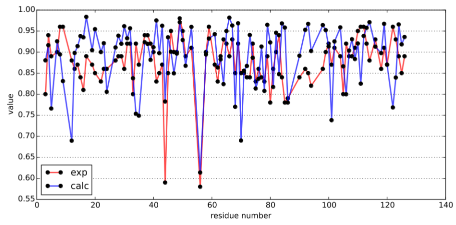
	Holo 6 ns 313 K	2MM3 PDB 298 corrected
	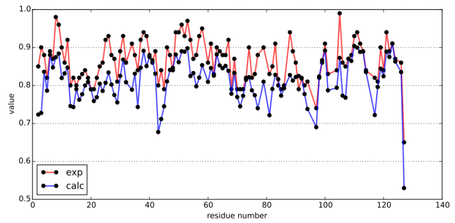	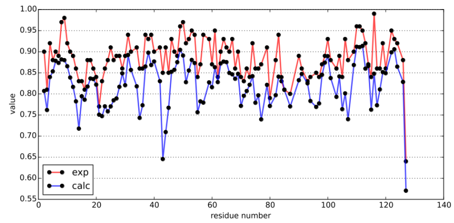
	Holo 6 ns 283 K corrected	Holo 6 ns 291 K corrected
	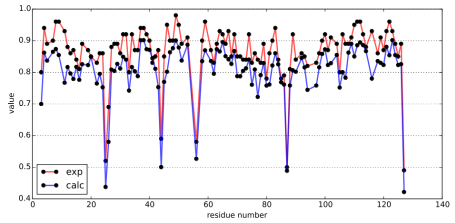	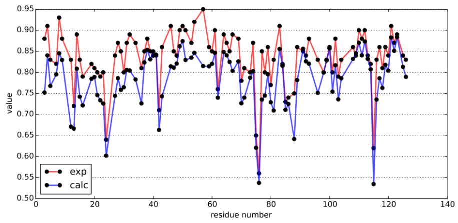
	Holo 6 ns 298 K corrected	Holo 6 ns 313 K corrected

E

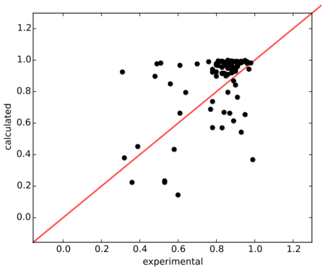

1O1U PDB 313

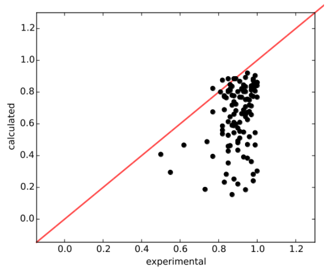

Apo 2 ns 283 K

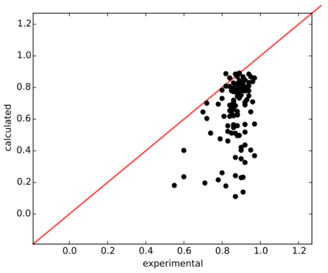

Apo 2 ns 291 K

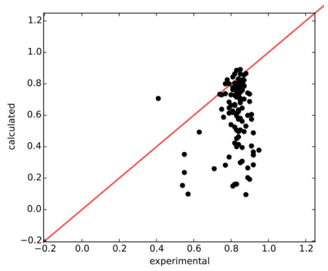

Apo 2 ns 298 K

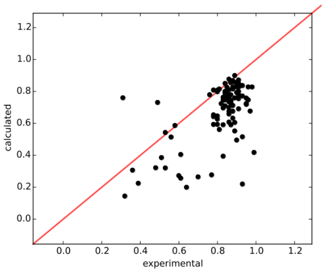

Apo 2 ns 313 K

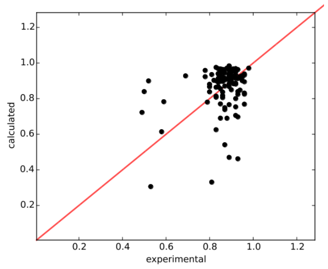

2MM3 PDB 298

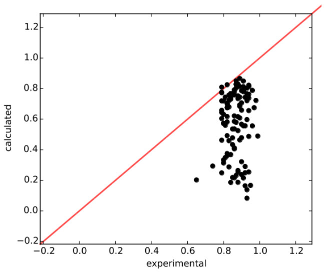

Holo 2 ns 283 K

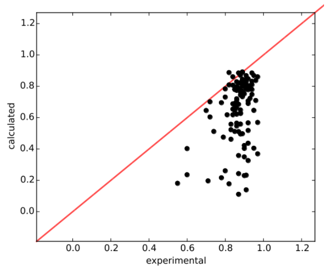

Holo 2 ns 291 K

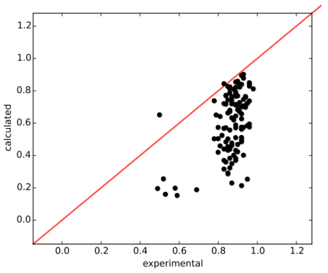

Holo 2 ns 298 K

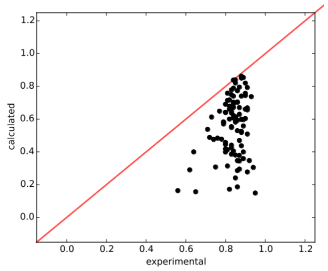

Holo 2 ns 313 K

F		
	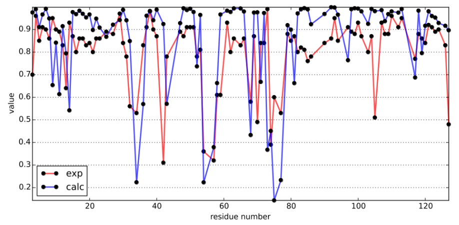	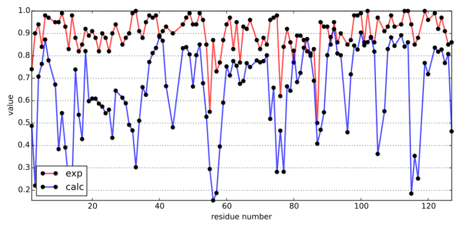
	1O1U PDB 313	Apo 2 ns 283 K
	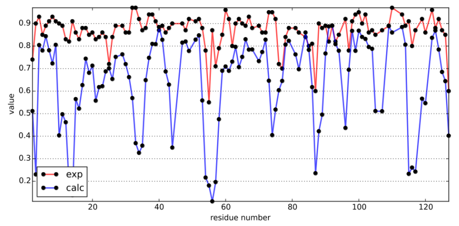	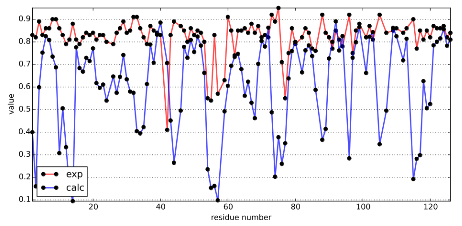
	Apo 2 ns 291 K	Apo 2 ns 298 K
	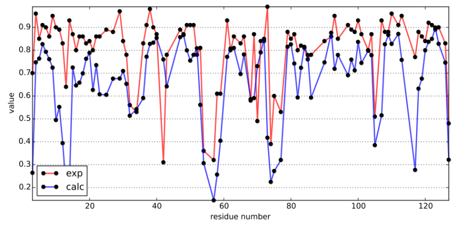	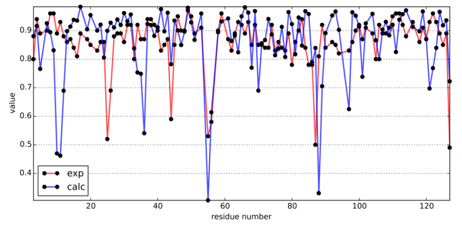
	Apo 2 ns 313 K	2MM3 PDB 298
	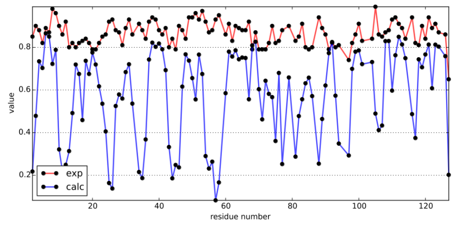	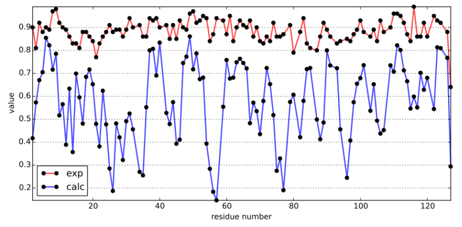
	Holo 2 ns 283 K	Holo 2 ns 291 K
	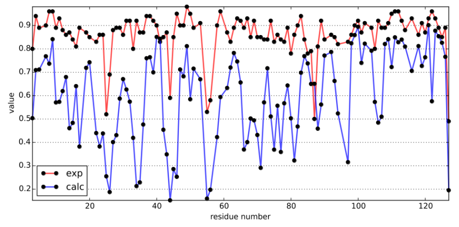	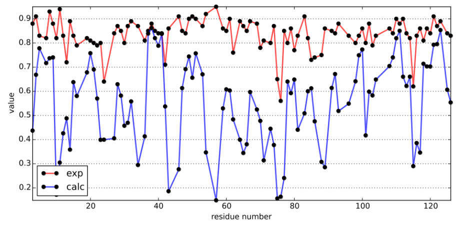
	Holo 2 ns 298 K	Holo 2 ns 313 K
