# Supplementary figures and images for: Different modes of barrel opening suggest a complex pathway of ligand binding in human gastrotropin
Source: PLoS One. 2019 May 10;14(5):e0216142. doi: 10.1371/journal.pone.0216142 (PMC6510414; doi:10.1371/journal.pone.0216142)

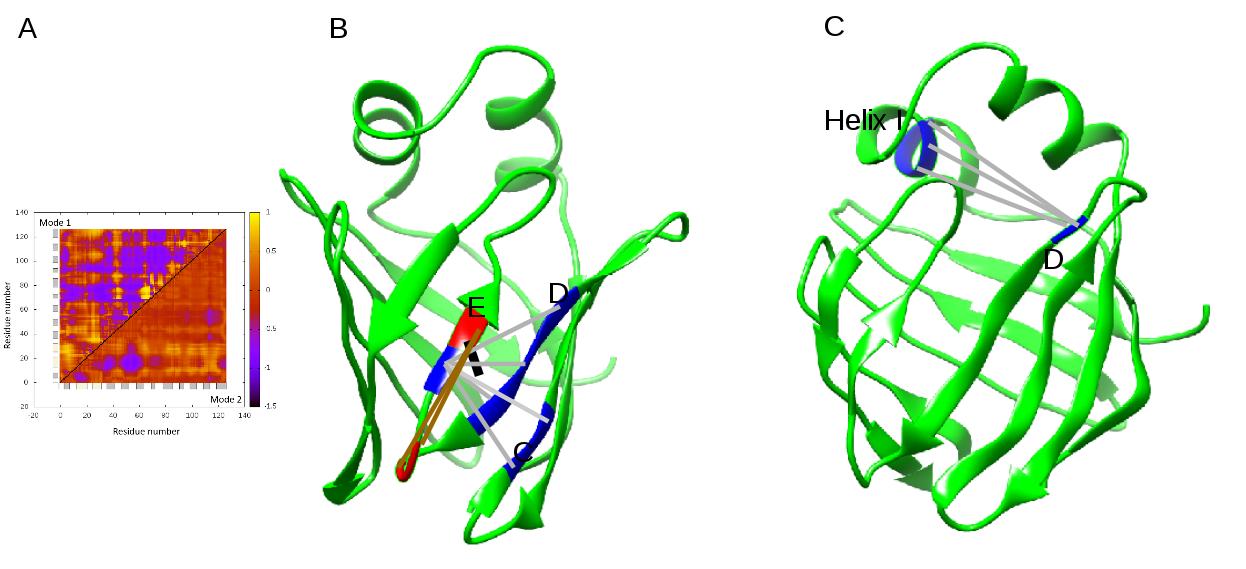

Supplement: S2 Fig — (A) Cα atom distance correlation matrix with PC1 (above diagonal) and PC2 (below diagonal) for each amino acid pair. Helical regions are labeled with beige rectangles, β strand regions with gray rectangles (B) The highest (red, brown lines) and lowest (blue, gray lines) distances depicted on the structure correalted with PC1 (C) The lowest (blue, gray lines) distances depicted on the structure correalted with PC2. (JPG) [file pone.0216142.s002.jpg]

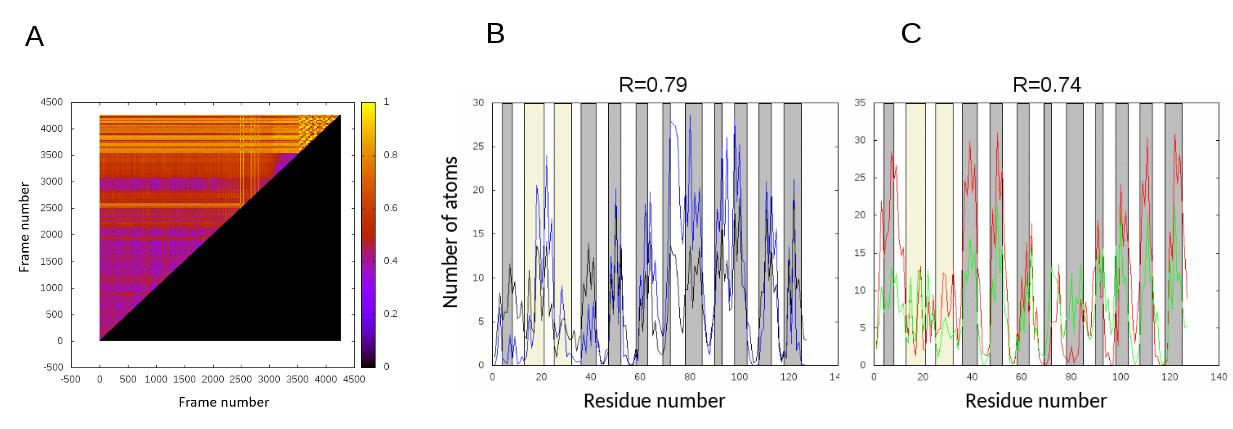

Supplement: S3 Fig — (A) Tanimoto distances of the simulation frames and the docked frames based on ligand contact data (see text) (B) Comparison of the average of the number of ligand heavy atoms (vertical axis) being closer, than 4 Å to each amino acid (horizontal axis) of CHO (blue all frames, black docked frames). (C). Comparison of the average of the number of ligand heavy atoms (vertical axis) being closer, than 4 Å to each amino acid (horizontal axis) of GCH: green simulated frames, red docked frames. Correlations are listed on the top. (JPG) [file pone.0216142.s003.jpg]

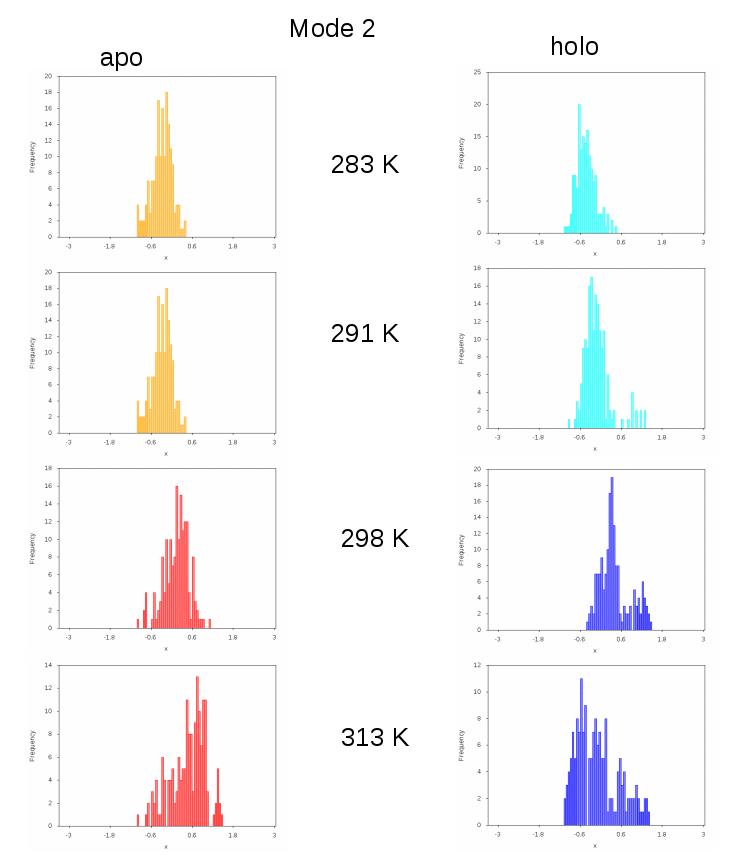

Supplement: S4 Fig — (JPG) [file pone.0216142.s004.jpg]
