# Supplementary material for: Different modes of barrel opening suggest a complex pathway of ligand binding in human gastrotropin
Source: PLoS One. 2019 May 10;14(5):e0216142. doi: 10.1371/journal.pone.0216142 (PMC6510414; doi:10.1371/journal.pone.0216142)
Supplement: S1 Table — The first column contains the specification of the hydrogen bonds in the structures as: a___a__. (PDF) [file pone.0216142.s005.pdf]

	3.7							
	Apo 283	*Apo 291*	*Apo 298*	*Apo 313*	*Holo 283*	*Holo 291*	*Holo 298*	*Holo 313*
a_100_N__a_110_OE1	0	0	0	0	0	0	2	0
a_100_N__a_111_O	230	233	232	226	226	223	204	226
a_100_N__a_99_OE1	4	0	1	1	0	0	0	0
a_100_OG1__a_100_O	0	0	0	0	0	0	0	0
a_100_OG1__a_101_O	0	0	0	0	0	2	0	7
a_100_OG1__a_111_O	13	26	18	30	9	23	5	20
a_100_OG1__a_90_O	7	10	15	6	31	9	25	17
a_100_OG1__a_98_ND1	0	0	0	0	0	0	0	0
a_100_OG1__a_98_NE2	0	0	0	0	2	0	1	0
a_100_OG1__a_99_O	0	0	0	0	0	1	0	0
a_101_N__a_100_OG1	0	0	0	0	0	0	0	0
a_101_N__a_90_O	233	222	225	229	92	68	82	73
a_101_N__a_99_OE1	0	0	1	0	0	0	0	0
a_101_OG__a_100_O	0	0	0	0	0	0	0	0
a_101_OG__a_101_O	0	0	0	0	0	0	0	0
a_101_OG__a_109_O	43	96	62	38	30	0	0	5
a_101_OG__a_110_OE1	1	2	3	2	4	9	10	10
a_101_OG__a_110_OE2	4	2	3	2	54	22	76	18
**a_101_OG__a_201_O**	**0**	**0**	**0**	**0**	**13**	**53**	**0**	**15**
**a_101_OG__a_201_O1**	**0**	**0**	**0**	**0**	**25**	**0**	**14**	**35**
**a_101_OG__a_201_O3**	**0**	**0**	**0**	**0**	**0**	**0**	**0**	**0**
a_101_OG__a_90_O	11	5	5	18	24	54	14	52
a_101_OG__a_99_OE1	25	30	23	31	0	2	0	8
a_102_N__a_100_O	0	0	0	0	0	0	0	0
a_102_N__a_101_OG	0	0	0	0	0	0	0	0
a_102_N__a_109_O	220	224	217	221	213	211	160	204
**a_102_N__a_201_O**	**0**	**0**	**0**	**0**	**0**	**1**	**0**	**0**
**a_102_N__a_201_O1**	**0**	**0**	**0**	**0**	**0**	**0**	**0**	**0**
a_103_N__a_102_OE1	2	0	0	1	37	38	22	34
a_103_N__a_102_OE2	2	0	1	2	35	45	27	46
a_103_N__a_88_O	32	145	92	113	0	0	0	1
a_104_N__a_102_O	0	0	0	0	0	0	0	0
a_104_N__a_107_O	221	218	212	219	226	228	220	211
a_105_N__a_103_O	0	0	0	0	0	0	0	0
a_105_N__a_107_O	0	15	15	14	0	0	0	4
a_106_N__a_104_O	0	0	0	0	0	0	0	0
a_106_N__a_106_OD1	0	0	0	0	0	0	0	0
a_106_N__a_106_OD2	0	0	0	0	0	0	0	0
a_107_NZ__a_106_OD1	2	6	1	6	1	5	6	1
a_107_NZ__a_106_OD2	0	4	1	0	2	7	4	6
a_107_NZ__a_10_OG	0	0	0	0	0	0	0	0
a_107_NZ__a_120_OE2	0	0	0	0	0	0	0	1
a_107_NZ__a_9_OE1	10	7	5	14	9	33	27	26
a_107_NZ__a_9_OE2	4	8	1	6	18	24	23	24
a_107_N__a_104_O	0	0	0	2	8	0	1	0
a_107_N__a_105_O	0	0	0	0	0	0	0	0
a_107_N__a_106_OD1	0	0	0	0	0	0	0	0
a_107_N__a_106_OD2	0	0	0	0	0	0	0	0

a_108_N__a_123_O	248	247	246	244	235	231	226	222
a_108_N__a_123_OG	0	0	0	0	0	0	0	1
a_109_N__a_102_O	237	238	232	231	220	225	226	213
a_10_N__a_11_OE1	0	0	0	0	1	5	3	6
a_10_N__a_11_OE2	0	0	0	0	2	11	9	12
a_10_N__a_122_O	101	125	71	67	1	82	0	58
a_10_N__a_8_SD	0	0	0	1	2	0	0	0
a_10_N__a_9_OE1	0	0	0	0	0	0	5	0
a_10_N__a_9_OE2	0	0	0	0	0	0	11	0
a_10_OG__a_10_O	0	0	0	0	0	0	0	0
a_10_OG__a_11_O	0	0	0	0	0	0	1	0
a_10_OG__a_11_OE1	0	0	0	0	2	0	0	1
a_10_OG__a_11_OE2	0	0	0	0	1	0	0	1
a_10_OG__a_120_O	0	0	0	0	0	0	0	0
a_10_OG__a_122_O	0	0	1	1	0	0	0	0
a_10_OG__a_9_O	0	0	0	0	47	0	2	7
a_10_OG__a_9_OE1	115	95	68	78	2	49	48	49
a_10_OG__a_9_OE2	99	79	97	74	1	53	55	40
a_110_N__a_121_O	232	230	229	229	228	221	172	233
a_111_N__a_100_O	236	244	229	228	201	221	215	216
a_111_N__a_110_OE2	0	0	0	0	0	0	0	0
a_111_N__a_99_OE1	0	0	0	0	0	0	0	0
a_112_N__a_119_O	240	228	234	215	213	229	213	210
a_112_OG__a_110_O	0	0	0	0	0	0	0	1
a_112_OG__a_110_OE1	1	4	1	0	0	1	9	4
a_112_OG__a_110_OE2	0	4	0	0	2	0	0	4
a_112_OG__a_111_O	0	0	0	0	0	0	0	0
a_112_OG__a_112_O	0	0	0	0	0	0	0	0
a_112_OG__a_119_O	4	2	11	7	13	12	54	9
a_112_OG__a_119_OH	0	0	0	0	0	0	0	0
**a_112_OG__a_201_O2**	**0**	**0**	**0**	**0**	**0**	**1**	**0**	**0**
a_112_OG__a_97_OH	0	0	0	0	11	5	11	1
a_112_OG__a_98_O	1	20	6	14	0	37	6	10
a_112_OG__a_99_OE1	35	29	27	29	8	17	9	2
a_113_N__a_112_OG	0	0	0	0	0	0	0	0
a_113_N__a_98_O	245	240	232	239	235	209	228	221
a_113_OG1__a_111_O	0	0	0	0	0	0	0	0
a_113_OG1__a_112_O	0	0	0	0	0	0	0	0
a_113_OG1__a_113_O	0	0	0	0	0	0	0	0
a_113_OG1__a_114_O	0	1	1	0	0	0	0	0
a_113_OG1__a_115_O	0	0	0	0	0	0	0	1
a_113_OG1__a_117_O	0	1	7	12	0	6	0	14
a_113_OG1__a_118_OG1	17	30	80	23	58	36	42	9
a_113_OG1__a_98_ND1	0	0	0	1	0	0	0	0
a_113_OG1__a_98_O	9	6	4	10	10	4	13	13
a_114_N__a_113_OG1	0	0	0	0	0	0	0	0
a_114_N__a_117_O	142	99	120	176	209	226	215	205
a_114_N__a_118_OG1	0	0	0	0	0	0	0	0
a_115_N__a_113_O	0	0	0	0	0	0	0	0
a_115_N__a_113_OG1	0	0	0	12	0	0	0	0

a_115_N__a_117_O	0	0	0	0	2	0	0	0
a_115_N__a_96_O	0	0	0	0	28	12	25	18
a_115_N__a_96_OD1	0	0	0	0	5	2	7	10
a_116_N__a_113_OG1	0	0	0	0	0	0	0	2
a_116_N__a_114_O	0	0	0	0	1	0	2	0
a_116_N__a_117_O	0	0	0	0	0	0	0	0
a_117_N__a_114_O	14	0	1	11	142	46	103	104
a_117_N__a_115_O	0	0	0	0	0	0	0	0
a_118_N__a_116_O	0	0	0	0	0	0	0	0
a_118_OG1__a_112_O	7	2	1	15	13	0	0	3
a_118_OG1__a_113_OG1	1	12	10	6	13	3	18	5
a_118_OG1__a_114_O	0	0	0	0	0	0	0	0
a_118_OG1__a_116_O	0	0	0	18	14	42	31	8
a_118_OG1__a_117_O	0	0	0	0	0	0	0	0
a_118_OG1__a_118_O	0	0	0	0	0	0	0	0
a_118_OG1__a_119_O	0	0	0	0	0	0	0	0
a_118_OG1__a_13_OD1	0	0	0	0	0	0	0	0
a_119_N__a_112_O	232	232	233	207	224	234	228	195
a_119_N__a_112_OG	0	0	0	0	0	0	0	6
a_119_N__a_118_OG1	0	0	0	0	0	0	0	0
a_119_OH__a_110_OE1	0	1	1	14	0	0	0	6
a_119_OH__a_110_OE2	0	0	0	1	0	0	0	2
a_119_OH__a_112_OG	0	0	0	0	0	0	0	0
a_119_OH__a_11_OE1	22	13	40	45	35	14	0	35
a_119_OH__a_11_OE2	95	92	94	96	6	32	0	3
a_119_OH__a_12_O	0	0	0	8	0	12	24	3
a_119_OH__a_13_O	0	0	0	0	0	5	0	0
a_119_OH__a_14_OH	0	0	0	0	0	0	0	0
**a_119_OH__a_201_N**	**0**	**0**	**0**	**0**	**0**	**0**	**0**	**0**
**a_119_OH__a_201_O2**	**0**	**0**	**0**	**0**	**0**	**5**	**0**	**20**
**a_119_OH__a_201_O4**	**0**	**0**	**0**	**0**	**5**	**3**	**58**	**16**
**a_119_OH__a_201_O5**	**0**	**0**	**0**	**0**	**19**	**7**	**54**	**12**
a_119_OH__a_74_SD	0	1	0	0	0	0	0	0
a_119_OH__a_8_SD	0	0	0	0	0	0	0	0
a_119_OH__a_97_OH	0	0	0	1	0	0	0	2
a_119_OH__a_99_OE1	0	0	0	0	0	0	0	2
a_11_N__a_10_OG	0	0	0	0	0	0	0	0
a_11_N__a_11_OE1	6	5	2	1	3	4	28	12
a_11_N__a_11_OE2	3	4	3	1	3	12	21	22
a_120_N__a_120_OE1	0	0	0	0	0	0	0	0
a_120_N__a_120_OE2	0	0	0	0	0	0	0	0
a_120_N__a_12_O	21	15	0	42	135	127	105	110
a_120_N__a_13_OD1	0	0	0	1	0	0	0	4
a_121_NE__a_110_OE1	0	0	27	30	0	0	0	0
a_121_NE__a_110_OE2	0	1	34	42	0	0	0	12
a_121_NE__a_112_OG	0	0	0	0	0	0	0	0
a_121_NE__a_119_O	0	0	0	0	0	1	0	0
a_121_NE__a_119_OH	16	51	45	21	50	37	9	30
a_121_NE__a_11_OE1	10	0	1	12	0	0	0	1
a_121_NE__a_11_OE2	18	15	2	0	0	9	0	5

a_121_NE__a_123_OG	1	0	0	0	0	0	0	0
a_121_NE__a_14_OH	0	0	0	0	0	0	0	0
a_121_NE__a_16_OE1	0	0	0	0	0	0	0	3
a_121_NE__a_16_OE2	0	0	0	0	0	0	0	2
**a_121_NE__a_201_N**	**0**	**0**	**0**	**0**	**0**	**0**	**0**	**0**
**a_121_NE__a_201_O1**	**0**	**0**	**0**	**0**	**0**	**0**	**0**	**3**
**a_121_NE__a_201_O2**	**0**	**0**	**0**	**0**	**2**	**4**	**5**	**13**
**a_121_NE__a_201_O4**	**0**	**0**	**0**	**0**	**2**	**2**	**42**	**4**
**a_121_NE__a_201_O5**	**0**	**0**	**0**	**0**	**3**	**2**	**17**	**5**
a_121_NE__a_74_SD	0	0	1	0	0	0	0	0
a_121_NE__a_8_SD	7	4	1	4	9	3	0	5
a_121_NH1__a_110_OE1	34	53	53	40	140	137	113	115
a_121_NH1__a_110_OE2	94	107	64	46	47	53	33	46
a_121_NH1__a_112_OG	0	0	0	0	3	0	18	0
a_121_NH1__a_119_OH	1	9	4	5	0	0	0	0
a_121_NH1__a_11_OE1	0	0	0	7	0	0	0	0
a_121_NH1__a_11_OE2	0	0	0	6	0	0	0	0
a_121_NH1__a_121_O	0	0	0	1	0	0	0	0
a_121_NH1__a_123_OG	11	8	12	8	0	0	0	0
a_121_NH1__a_14_OH	0	0	1	0	0	0	0	0
**a_121_NH1__a_201_N**	**0**	**0**	**0**	**0**	**0**	**0**	**0**	**2**
**a_121_NH1__a_201_O1**	**0**	**0**	**0**	**0**	**2**	**0**	**1**	**0**
**a_121_NH1__a_201_O2**	**0**	**0**	**0**	**0**	**6**	**12**	**0**	**23**
**a_121_NH1__a_201_O3**	**0**	**0**	**0**	**0**	**12**	**42**	**30**	**14**
**a_121_NH1__a_201_O4**	**0**	**0**	**0**	**0**	**1**	**0**	**4**	**7**
**a_121_NH1__a_201_O5**	**0**	**0**	**0**	**0**	**0**	**1**	**0**	**6**
**a_121_NH1__a_202_O3**	**0**	**0**	**0**	**0**	**1**	**19**	**29**	**7**
**a_121_NH1__a_202_O7**	**0**	**0**	**0**	**0**	**7**	**8**	**22**	**8**
a_121_NH1__a_51_OE1	1	8	6	0	0	0	0	0
a_121_NH1__a_53_OH	0	0	0	4	0	0	0	0
a_121_NH1__a_73_O	0	0	1	0	0	0	0	0
a_121_NH1__a_74_SD	0	2	3	1	0	0	0	0
a_121_NH1__a_8_SD	0	0	0	0	0	0	0	0
a_121_NH1__a_97_OH	0	0	0	0	0	0	0	2
a_121_NH1__a_99_OE1	0	0	0	0	2	1	0	1
a_121_NH2__a_110_OE1	5	12	29	26	1	0	0	7
a_121_NH2__a_110_OE2	2	2	24	27	0	0	0	8
a_121_NH2__a_112_OG	0	0	0	0	0	1	2	0
a_121_NH2__a_119_O	0	0	0	0	0	1	0	0
a_121_NH2__a_119_OH	4	14	12	5	4	12	0	4
a_121_NH2__a_11_OE1	3	0	0	3	0	0	0	0
a_121_NH2__a_11_OE2	11	3	0	0	0	0	0	3
a_121_NH2__a_13_OD1	0	0	0	0	0	0	0	1
a_121_NH2__a_14_OH	6	0	6	2	0	0	0	1
a_121_NH2__a_16_OE2	0	0	0	0	0	0	0	2
**a_121_NH2__a_201_N**	**0**	**0**	**0**	**0**	**0**	**3**	**2**	**1**
**a_121_NH2__a_201_O2**	**0**	**0**	**0**	**0**	**12**	**38**	**29**	**25**
**a_121_NH2__a_201_O3**	**0**	**0**	**0**	**0**	**1**	**0**	**4**	**8**
**a_121_NH2__a_201_O4**	**0**	**0**	**0**	**0**	**32**	**23**	**19**	**16**
**a_121_NH2__a_201_O5**	**0**	**0**	**0**	**0**	**45**	**23**	**10**	**23**

**a_121_NH2__a_202_O7**	**0**	**0**	**0**	**0**	**1**	**3**	**48**	**10**
a_121_NH2__a_31_O	0	0	0	1	0	0	0	0
a_121_NH2__a_51_OE1	8	4	9	1	0	0	0	0
a_121_NH2__a_53_OH	0	0	0	3	0	0	0	0
a_121_NH2__a_74_SD	0	6	6	3	0	0	1	0
a_121_NH2__a_8_SD	6	1	0	2	0	2	0	0
a_121_NH2__a_97_OH	0	0	0	0	2	0	0	6
a_121_NH2__a_99_OE1	0	0	0	1	0	0	0	0
a_121_N__a_110_O	239	232	235	240	243	242	245	244
a_121_N__a_119_O	0	0	0	0	0	0	0	0
a_122_N__a_10_O	183	204	128	99	198	156	6	109
a_122_N__a_10_OG	9	0	2	2	0	0	0	1
a_122_N__a_120_O	0	0	0	0	0	0	0	0
a_122_N__a_8_SD	0	0	0	0	0	1	0	5
a_122_N__a_9_O	0	0	0	0	0	4	1	1
a_123_N__a_108_O	241	246	236	241	240	236	216	225
**a_123_N__a_201_O**	**0**	**0**	**0**	**0**	**0**	**0**	**0**	**0**
a_123_OG__a_108_O	10	16	34	12	5	6	13	15
a_123_OG__a_121_O	0	0	0	0	0	0	1	0
a_123_OG__a_122_O	0	0	0	0	0	0	0	0
a_123_OG__a_123_O	0	0	0	0	0	0	0	0
a_123_OG__a_124_O	0	0	0	0	1	1	2	2
**a_123_OG__a_201_O**	**0**	**0**	**0**	**0**	**0**	**0**	**0**	**1**
**a_123_OG__a_201_O1**	**0**	**0**	**0**	**0**	**0**	**3**	**0**	**4**
a_123_OG__a_7_O	8	0	2	2	146	113	112	92
a_123_OG__a_8_SD	0	0	1	5	0	0	0	0
a_124_NZ__a_106_OD1	3	2	11	11	7	7	8	7
a_124_NZ__a_106_OD2	2	8	10	2	3	12	15	9
a_124_NZ__a_125_O	3	4	18	8	25	21	25	11
a_124_NZ__a_7_OE1	3	2	5	8	30	15	21	15
a_124_NZ__a_7_OE2	4	3	3	8	35	27	12	10
a_124_NZ__a_8_O	1	0	0	0	5	0	0	0
a_124_NZ__a_9_OE1	0	0	0	0	23	2	3	4
a_124_NZ__a_9_OE2	0	0	0	3	20	3	2	3
a_124_N__a_123_OG	0	0	0	0	0	0	0	0
a_124_N__a_7_O	237	237	243	242	213	207	221	196
a_125_NE__a_105_O	2	3	1	9	3	0	1	5
a_125_NE__a_106_O	0	0	0	0	14	1	10	10
a_125_NE__a_106_OD1	10	12	11	4	26	7	7	22
a_125_NE__a_106_OD2	18	4	11	10	38	13	14	25
a_125_NE__a_125_O	0	0	0	0	0	0	0	0
a_125_NE__a_126_O	5	5	9	2	1	0	4	2
a_125_NE__a_3_O	0	0	0	1	0	1	0	2
a_125_NE__a_5_O	0	0	0	0	0	0	1	0
a_125_NH1__a_105_O	7	9	27	22	1	1	7	9
a_125_NH1__a_106_O	0	0	0	0	0	0	0	3
a_125_NH1__a_106_OD1	14	11	0	2	8	7	6	4
a_125_NH1__a_106_OD2	5	4	2	0	1	7	4	11
a_125_NH1__a_125_O	3	1	1	1	0	0	1	2
a_125_NH1__a_126_O	0	0	0	4	0	0	0	3

a_125_NH1__a_3_O	0	0	1	0	2	0	0	4
a_125_NH1__a_5_O	0	0	0	1	0	0	0	0
a_125_NH2__a_105_O	0	0	0	0	2	0	0	0
a_125_NH2__a_106_O	0	0	0	0	1	0	0	0
a_125_NH2__a_106_OD1	4	16	8	4	42	12	19	45
a_125_NH2__a_106_OD2	21	9	14	12	70	13	5	23
a_125_NH2__a_126_O	0	0	0	0	0	0	0	0
a_125_NH2__a_3_O	0	0	2	4	0	0	0	2
a_125_N__a_106_O	237	237	225	235	81	131	171	117
a_125_N__a_106_OD1	0	0	0	0	0	0	0	0
a_125_N__a_106_OD2	0	0	0	0	0	0	0	0
a_126_N__a_124_O	0	0	0	0	0	0	0	0
a_126_N__a_5_O	209	212	214	211	190	222	201	192
a_127_N__a_125_O	0	0	0	0	0	0	0	0
a_127_N__a_5_O	0	1	5	4	3	6	8	3
a_12_NZ__a_10_O	1	0	0	0	0	19	86	36
a_12_NZ__a_10_OG	32	20	21	24	15	31	25	18
a_12_NZ__a_118_O	0	0	0	11	0	0	0	0
a_12_NZ__a_118_OG1	0	0	0	0	0	0	0	0
a_12_NZ__a_11_O	0	0	0	1	0	0	1	0
a_12_NZ__a_120_O	4	3	11	7	0	0	0	1
a_12_NZ__a_120_OE1	63	51	46	55	54	46	62	52
a_12_NZ__a_120_OE2	43	65	72	44	45	69	45	55
a_12_NZ__a_13_OD1	0	0	0	4	1	0	0	0
a_12_NZ__a_16_OE1	0	0	0	1	0	0	0	2
a_12_NZ__a_16_OE2	0	0	0	1	0	0	0	0
a_12_NZ__a_9_OE1	1	0	1	1	0	0	0	1
a_12_NZ__a_9_OE2	0	0	1	0	0	0	0	0
a_12_N__a_10_O	0	0	0	0	0	0	0	0
a_12_N__a_119_OH	44	19	46	25	0	0	0	0
a_12_N__a_11_OE1	0	0	3	13	0	0	0	1
a_12_N__a_11_OE2	1	0	8	9	0	0	0	2
a_12_N__a_120_O	157	171	86	68	217	179	121	160
**a_12_N__a_201_O4**	**0**	**0**	**0**	**0**	**0**	**0**	**13**	**0**
**a_12_N__a_201_O5**	**0**	**0**	**0**	**0**	**0**	**0**	**3**	**0**
a_13_ND2__a_118_O	76	27	20	72	166	128	117	132
a_13_ND2__a_118_OG1	0	0	0	0	0	0	0	0
a_13_ND2__a_119_OH	0	0	0	0	0	0	0	0
a_13_ND2__a_120_O	0	0	0	0	0	0	0	0
a_13_ND2__a_12_O	0	0	1	0	0	0	0	1
a_13_ND2__a_13_O	3	0	5	6	4	6	16	6
a_13_ND2__a_15_OD1	0	2	14	10	0	0	0	0
a_13_ND2__a_15_OD2	1	2	5	13	0	0	0	0
a_13_ND2__a_16_O	1	0	0	0	0	0	0	0
a_13_ND2__a_16_OE1	12	12	9	19	0	4	0	11
a_13_ND2__a_16_OE2	9	10	12	8	3	8	5	6
a_13_ND2__a_8_SD	0	0	0	0	0	0	0	0
a_13_N__a_119_OH	2	0	4	33	0	0	0	0
a_13_N__a_11_O	0	0	0	0	0	0	0	1
a_13_N__a_11_OE1	0	0	2	5	1	4	0	0

a_13_N__a_11_OE2	0	0	10	5	0	8	0	0
a_13_N__a_13_OD1	0	0	0	0	0	0	0	0
a_14_N__a_119_OH	0	0	0	0	0	0	0	0
a_14_N__a_11_O	0	0	0	0	0	0	0	0
a_14_N__a_11_OE1	0	0	8	37	1	0	0	1
a_14_N__a_11_OE2	0	0	3	32	0	0	0	1
a_14_N__a_12_O	10	4	5	0	0	0	0	0
a_14_N__a_13_OD1	0	0	0	0	0	0	0	0
a_14_N__a_15_OD1	0	10	2	0	0	0	0	0
a_14_N__a_15_OD2	0	3	0	0	0	0	0	0
**a_14_N__a_201_O4**	**0**	**0**	**0**	**0**	**0**	**0**	**0**	**0**
a_14_OH__a_112_OG	0	0	1	1	0	0	0	0
a_14_OH__a_119_OH	0	0	0	0	0	0	0	0
a_14_OH__a_11_OE1	0	1	0	0	14	3	4	6
a_14_OH__a_11_OE2	0	0	1	0	0	4	8	3
a_14_OH__a_15_OD2	0	0	0	0	0	8	0	0
a_14_OH__a_18_SD	0	0	0	0	0	0	0	0
**a_14_OH__a_201_N**	**0**	**0**	**0**	**0**	**0**	**0**	**0**	**0**
**a_14_OH__a_201_O4**	**0**	**0**	**0**	**0**	**16**	**6**	**0**	**0**
**a_14_OH__a_201_O5**	**0**	**0**	**0**	**0**	**19**	**14**	**0**	**3**
a_14_OH__a_24_O	0	0	0	0	0	0	0	0
a_14_OH__a_27_O	0	1	2	0	0	0	0	0
a_14_OH__a_28_O	119	11	29	22	2	0	26	24
a_14_OH__a_29_OE1	0	0	0	3	2	1	0	1
a_14_OH__a_29_OE2	0	1	0	3	1	2	0	0
a_14_OH__a_53_OH	0	0	0	0	0	0	0	0
a_14_OH__a_73_OG1	0	0	0	0	0	0	0	0
a_14_OH__a_74_O	0	1	2	1	0	0	0	0
a_14_OH__a_74_SD	1	39	22	18	0	0	0	0
a_14_OH__a_97_OH	0	0	0	0	0	0	0	0
a_15_N__a_12_O	0	0	0	0	0	0	0	4
a_15_N__a_13_O	0	0	0	0	0	0	0	0
a_15_N__a_13_OD1	2	8	6	0	0	1	0	0
a_15_N__a_15_OD1	0	0	0	0	0	0	0	0
a_15_N__a_15_OD2	0	0	0	0	0	0	0	0
a_15_N__a_16_OE1	0	0	0	0	0	0	0	0
a_16_N__a_13_O	4	0	3	28	2	8	12	20
a_16_N__a_13_OD1	17	49	32	12	2	19	0	0
a_16_N__a_14_O	0	0	0	0	0	0	0	0
a_16_N__a_15_OD1	0	0	0	0	0	0	0	0
a_16_N__a_15_OD2	0	0	0	0	0	0	0	1
a_16_N__a_16_O	0	0	0	0	0	0	0	0
a_16_N__a_16_OE1	0	0	0	0	0	5	1	0
a_16_N__a_16_OE2	0	0	1	0	0	0	0	0
a_17_N__a_13_O	4	0	2	17	21	32	37	48
a_17_N__a_13_OD1	0	0	0	0	0	0	0	0
a_17_N__a_14_O	81	39	107	76	34	24	30	21
a_17_N__a_15_O	0	0	0	0	0	0	0	0
a_18_N__a_14_O	230	201	207	214	182	173	196	157
a_18_N__a_15_O	0	1	3	1	0	2	1	0

a_18_N__a_16_O	0	0	0	0	0	0	0	0
a_18_N__a_18_SD	0	0	0	0	0	0	0	0
a_19_NZ__a_15_O	9	3	0	0	5	1	8	6
a_19_NZ__a_15_OD1	1	3	3	7	3	7	14	19
a_19_NZ__a_15_OD2	1	11	7	2	3	9	24	17
a_19_NZ__a_16_O	0	0	1	0	0	1	0	0
a_19_NZ__a_16_OE1	72	63	62	55	83	67	64	51
a_19_NZ__a_16_OE2	61	66	77	69	77	84	68	63
a_19_NZ__a_24_O	0	0	0	0	0	0	3	1
a_19_N__a_15_O	148	172	181	181	53	94	114	106
a_19_N__a_16_O	18	67	49	19	99	106	95	80
a_19_N__a_17_O	25	2	4	13	5	1	4	0
a_19_N__a_18_SD	0	0	0	0	0	0	0	0
a_19_N__a_23_O	0	0	0	0	0	0	0	0
**a_201_O__a_101_O**	**0**	**0**	**0**	**0**	**4**	**0**	**0**	**4**
**a_201_O__a_101_OG**	**0**	**0**	**0**	**0**	**12**	**13**	**11**	**11**
**a_201_O__a_102_O**	**0**	**0**	**0**	**0**	**0**	**1**	**0**	**0**
**a_201_O__a_108_O**	**0**	**0**	**0**	**0**	**0**	**0**	**0**	**1**
**a_201_O__a_109_O**	**0**	**0**	**0**	**0**	**0**	**0**	**0**	**2**
**a_201_O__a_110_OE1**	**0**	**0**	**0**	**0**	**17**	**17**	**0**	**29**
**a_201_O__a_110_OE2**	**0**	**0**	**0**	**0**	**20**	**95**	**23**	**27**
**a_201_O__a_201_O1**	**0**	**0**	**0**	**0**	**0**	**2**	**7**	**0**
**a_201_O__a_202_O3**	**0**	**0**	**0**	**0**	**31**	**13**	**16**	**21**
**a_201_O__a_2_O**	**0**	**0**	**0**	**0**	**1**	**0**	**9**	**0**
**a_201_O__a_85_SD**	**0**	**0**	**0**	**0**	**0**	**0**	**0**	**0**
**a_201_O__a_88_O**	**0**	**0**	**0**	**0**	**8**	**4**	**1**	**22**
**a_201_O__a_90_O**	**0**	**0**	**0**	**0**	**0**	**0**	**0**	**0**
**a_201_O__a_99_OE1**	**0**	**0**	**0**	**0**	**10**	**0**	**1**	**0**
a_20_N__a_16_O	11	97	46	6	24	46	36	44
a_20_N__a_17_O	69	66	67	99	113	114	112	120
a_20_N__a_18_O	0	0	0	0	0	0	0	0
a_21_N__a_17_O	15	44	27	12	11	27	3	28
a_21_N__a_18_O	14	16	28	29	61	62	67	47
a_21_N__a_19_O	0	0	0	0	0	0	0	0
a_22_N__a_18_O	33	130	95	91	111	115	114	154
a_22_N__a_19_O	2	16	36	28	10	6	11	7
a_22_N__a_20_O	0	0	0	0	0	0	0	0
a_23_N__a_18_O	30	143	88	88	90	84	157	129
a_23_N__a_19_O	0	0	1	0	0	0	0	0
**a_23_N__a_202_OT2**	**0**	**0**	**0**	**0**	**0**	**0**	**0**	**0**
a_23_N__a_21_O	0	0	0	0	0	0	0	0
a_24_N__a_22_O	0	0	0	0	0	0	0	0
a_24_N__a_74_SD	0	0	0	0	0	0	0	1
a_24_OG__a_15_O	0	0	0	0	0	0	0	0
a_24_OG__a_22_O	0	0	0	0	7	10	1	9
a_24_OG__a_23_O	0	0	0	0	0	0	0	0
a_24_OG__a_24_O	0	0	0	0	0	0	0	0
a_24_OG__a_26_OD1	15	15	32	10	0	0	0	1
a_24_OG__a_26_OD2	5	10	10	11	0	0	0	0
a_24_OG__a_75_O	0	4	0	0	0	0	0	0

a_25_N__a_15_OD2	0	0	0	0	0	0	1	0
**a_25_N__a_201_O4**	**0**	**0**	**0**	**0**	**0**	**0**	**0**	**1**
a_25_N__a_23_O	0	0	0	0	0	0	0	0
a_25_N__a_24_OG	0	0	0	0	0	0	0	0
a_25_N__a_74_SD	0	0	0	0	0	0	0	1
a_25_OG__a_14_OH	0	0	0	2	0	0	0	2
a_25_OG__a_15_OD1	0	0	4	7	0	0	0	0
a_25_OG__a_15_OD2	0	1	4	4	0	0	1	0
a_25_OG__a_18_SD	0	0	0	0	0	0	1	0
**a_25_OG__a_201_O4**	**0**	**0**	**0**	**0**	**0**	**0**	**0**	**4**
**a_25_OG__a_201_O5**	**0**	**0**	**0**	**0**	**0**	**0**	**0**	**1**
a_25_OG__a_23_O	0	1	0	0	0	1	0	0
a_25_OG__a_24_O	0	0	0	0	0	10	0	0
a_25_OG__a_24_OG	0	0	0	0	2	1	6	1
a_25_OG__a_25_O	0	0	0	0	0	0	0	0
a_25_OG__a_26_OD1	0	0	0	0	0	0	0	1
a_25_OG__a_29_OE1	0	0	5	1	2	7	1	10
a_25_OG__a_29_OE2	0	0	3	0	3	6	4	3
a_25_OG__a_74_O	0	0	0	0	0	0	0	1
a_25_OG__a_74_SD	0	0	0	0	0	0	0	1
a_26_N__a_24_O	0	0	0	0	0	0	0	0
a_26_N__a_24_OG	2	4	1	5	1	1	1	0
a_26_N__a_25_OG	0	0	0	0	0	0	0	0
a_26_N__a_26_OD1	0	0	0	0	0	0	0	0
a_26_N__a_26_OD2	0	0	0	0	0	0	0	0
a_26_N__a_29_OE1	0	0	6	0	0	0	0	1
a_26_N__a_29_OE2	0	0	11	0	0	0	0	2
a_27_N__a_24_O	140	87	84	96	0	0	5	8
a_27_N__a_24_OG	0	0	0	0	0	0	0	0
a_27_N__a_25_O	0	0	0	0	0	0	1	4
a_27_N__a_25_OG	0	0	0	0	0	0	0	3
a_27_N__a_26_OD1	0	0	0	0	0	0	0	0
a_27_N__a_26_OD2	0	0	0	0	0	0	0	0
a_28_N__a_24_O	19	5	14	16	0	0	0	3
a_28_N__a_25_O	3	46	27	21	75	46	21	32
a_28_N__a_25_OG	0	0	0	0	0	0	0	1
a_28_N__a_26_O	0	0	0	0	0	0	0	0
a_28_N__a_29_OE2	0	0	0	0	0	0	0	0
a_29_N__a_25_O	147	160	142	162	180	179	135	49
a_29_N__a_26_O	4	1	3	1	5	5	18	66
a_29_N__a_27_O	0	0	0	0	0	0	0	0
a_29_N__a_29_OE1	0	0	0	0	2	0	0	0
a_29_N__a_29_OE2	0	0	4	0	1	0	0	0
a_2_N__a_41_OE1	0	0	0	0	0	0	1	0
a_2_N__a_42_O	0	0	0	1	29	31	29	28
a_2_N__a_42_OE1	0	0	0	24	110	164	114	108
a_2_N__a_85_SD	0	0	0	1	0	0	0	0
a_2_N__a_87_O	0	0	0	0	0	0	0	0
a_2_N__a_88_O	0	0	0	0	2	0	2	4
**a_30_NZ__a_201_O4**	**0**	**0**	**0**	**0**	**0**	**0**	**0**	**0**

**a_30_NZ__a_201_O5**	**0**	**0**	**0**	**0**	**2**	**0**	**0**	**0**
a_30_NZ__a_25_O	0	0	0	1	1	0	0	0
a_30_NZ__a_26_O	20	21	18	27	26	27	26	12
a_30_NZ__a_26_OD1	76	59	80	52	48	62	67	85
a_30_NZ__a_26_OD2	80	98	84	60	66	60	33	74
a_30_NZ__a_27_O	0	0	0	1	0	0	0	0
a_30_NZ__a_29_OE1	0	0	0	1	8	13	8	9
a_30_NZ__a_29_OE2	0	0	0	1	1	2	0	0
a_30_NZ__a_33_OD1	1	0	0	1	0	0	0	0
a_30_NZ__a_53_OH	0	0	0	0	6	0	0	11
a_30_NZ__a_54_O	0	0	0	0	0	0	2	2
a_30_NZ__a_54_OG	0	0	0	0	0	0	2	0
a_30_NZ__a_56_O	0	0	0	1	0	0	0	0
a_30_NZ__a_57_ND1	0	1	0	0	0	0	0	0
a_30_NZ__a_75_O	0	0	2	0	0	0	0	0
a_30_N__a_26_O	84	68	74	43	39	39	16	113
a_30_N__a_27_O	7	7	9	21	9	17	29	18
a_30_N__a_28_O	0	0	0	0	0	0	0	0
a_31_N__a_27_O	139	88	94	83	152	184	170	145
a_31_N__a_28_O	11	44	26	46	4	2	3	4
a_31_N__a_29_O	0	0	0	0	0	0	0	0
a_32_NE__a_11_OE1	28	41	72	60	14	18	7	11
a_32_NE__a_11_OE2	22	38	38	48	30	10	18	3
a_32_NE__a_12_O	0	0	0	0	0	0	0	0
a_32_NE__a_13_OD1	0	0	0	1	0	0	0	0
a_32_NE__a_14_OH	1	0	0	2	3	2	12	5
a_32_NE__a_15_OD1	4	0	0	0	0	0	0	0
a_32_NE__a_15_OD2	2	0	0	8	0	0	0	0
a_32_NE__a_28_O	0	0	0	0	1	0	0	5
a_32_NE__a_29_OE1	0	3	0	2	21	17	28	18
a_32_NE__a_29_OE2	0	3	0	11	23	35	25	24
a_32_NE__a_33_OD1	0	0	0	0	0	0	2	0
a_32_NH1__a_11_O	0	1	0	0	0	0	0	0
a_32_NH1__a_11_OE1	0	11	1	0	25	37	5	10
a_32_NH1__a_11_OE2	0	4	2	0	30	10	8	27
a_32_NH1__a_12_O	0	4	2	0	0	0	0	0
a_32_NH1__a_13_OD1	0	0	1	3	0	0	0	0
a_32_NH1__a_14_OH	0	0	0	0	8	1	19	10
a_32_NH1__a_15_OD1	12	41	5	11	18	35	12	21
a_32_NH1__a_15_OD2	19	27	28	26	27	3	10	20
a_32_NH1__a_16_OE2	0	0	0	1	0	0	0	0
a_32_NH1__a_18_SD	0	0	0	0	0	1	0	0
**a_32_NH1__a_201_O4**	**0**	**0**	**0**	**0**	**1**	**0**	**0**	**0**
**a_32_NH1__a_201_O5**	**0**	**0**	**0**	**0**	**3**	**0**	**0**	**0**
a_32_NH1__a_28_O	0	0	0	1	3	0	1	1
a_32_NH1__a_29_OE1	11	21	9	19	5	6	5	11
a_32_NH1__a_29_OE2	22	14	6	21	3	6	16	21
a_32_NH1__a_32_O	0	0	1	0	0	0	0	0
a_32_NH1__a_33_OD1	0	0	0	0	2	1	5	0
a_32_NH2__a_119_OH	0	0	0	0	0	0	0	0

a_32_NH2__a_11_O	1	0	0	1	0	0	8	5
a_32_NH2__a_11_OE1	60	54	95	70	29	12	8	5
a_32_NH2__a_11_OE2	35	32	37	34	25	20	3	9
a_32_NH2__a_12_O	3	20	48	17	0	0	0	0
a_32_NH2__a_13_OD1	0	0	0	8	0	0	0	0
a_32_NH2__a_14_O	0	0	0	0	0	0	0	0
a_32_NH2__a_14_OH	0	0	0	1	1	0	1	1
a_32_NH2__a_15_OD1	26	47	60	39	18	2	18	11
a_32_NH2__a_15_OD2	19	46	10	19	2	21	2	24
a_32_NH2__a_18_SD	0	0	0	0	0	0	0	0
a_32_NH2__a_25_OG	0	0	0	0	0	1	0	0
a_32_NH2__a_28_O	0	0	0	0	0	0	0	9
a_32_NH2__a_29_OE1	5	3	5	18	34	36	61	35
a_32_NH2__a_29_OE2	1	6	2	9	39	38	35	25
a_32_NH2__a_33_OD1	0	0	0	0	0	0	0	0
a_32_N__a_14_OH	0	0	0	0	1	0	0	0
a_32_N__a_28_O	28	73	61	56	124	148	86	33
a_32_N__a_29_O	63	34	40	27	8	3	22	57
a_32_N__a_30_O	0	0	0	0	0	0	0	0
a_33_ND2__a_11_OE1	0	0	0	1	0	0	0	0
a_33_ND2__a_29_O	3	3	2	11	46	49	27	26
a_33_ND2__a_29_OE1	0	0	1	1	10	4	7	4
a_33_ND2__a_29_OE2	0	0	0	1	5	5	2	2
a_33_ND2__a_30_O	1	8	0	1	0	2	0	0
a_33_ND2__a_32_O	0	0	0	0	0	0	0	0
a_33_ND2__a_33_O	0	1	0	0	0	0	0	0
a_33_ND2__a_53_O	0	9	1	0	0	0	1	0
a_33_ND2__a_53_OH	0	0	0	0	0	0	0	0
a_33_ND2__a_54_O	0	9	2	13	0	0	3	0
a_33_ND2__a_54_OG	2	2	3	8	0	1	6	10
a_33_N__a_11_OE1	0	0	0	1	0	0	0	0
a_33_N__a_29_O	12	5	13	25	126	70	114	57
a_33_N__a_30_O	76	34	19	24	20	30	8	36
a_33_N__a_31_O	0	0	0	0	0	0	0	0
a_33_N__a_33_OD1	0	0	0	0	0	0	0	0
a_33_N__a_34_O	0	0	0	0	0	0	0	0
a_33_N__a_53_O	0	0	0	0	0	0	0	0
a_33_N__a_54_OG	0	0	0	2	0	0	0	0
a_34_N__a_29_O	0	0	0	0	1	0	0	0
a_34_N__a_30_O	46	24	32	47	166	148	168	152
a_34_N__a_31_O	8	13	9	12	5	17	11	5
a_34_N__a_32_O	0	0	0	0	0	0	0	0
a_34_N__a_33_OD1	0	0	0	0	0	0	1	0
a_34_N__a_53_O	0	1	5	0	0	0	0	0
a_34_N__a_54_OG	0	0	0	2	0	0	0	0
a_35_NZ__a_11_OE1	0	0	0	0	0	3	19	11
a_35_NZ__a_11_OE2	0	0	0	0	20	10	27	7
**a_35_NZ__a_201_O4**	**0**	**0**	**0**	**0**	**1**	**0**	**0**	**0**
**a_35_NZ__a_201_O5**	**0**	**0**	**0**	**0**	**1**	**0**	**0**	**0**
a_35_NZ__a_31_O	0	0	0	0	4	0	3	2

a_35_NZ__a_32_O	0	0	0	0	1	0	8	1
a_35_NZ__a_33_O	0	0	0	0	1	0	0	0
a_35_NZ__a_36_O	3	0	0	0	0	2	0	0
a_35_NZ__a_54_OG	1	0	0	0	0	0	0	0
a_35_NZ__a_7_OE1	53	31	62	44	66	56	21	29
a_35_NZ__a_7_OE2	60	45	67	84	70	56	16	31
a_35_NZ__a_8_O	16	24	21	14	52	52	41	67
a_35_NZ__a_8_SD	0	0	0	0	1	0	0	0
a_35_NZ__a_9_O	10	39	15	8	0	3	4	22
a_35_NZ__a_9_OE1	0	2	0	0	38	3	10	3
a_35_NZ__a_9_OE2	8	10	10	12	33	2	7	8
a_35_N__a_30_O	0	0	0	0	94	42	87	72
a_35_N__a_31_O	0	0	0	0	67	87	42	21
a_35_N__a_32_O	0	0	0	0	0	1	1	0
a_35_N__a_33_O	0	0	0	0	0	0	0	0
a_35_N__a_33_OD1	0	0	0	5	0	0	0	0
a_35_N__a_53_O	0	0	0	7	0	0	0	0
a_35_N__a_54_OG	0	0	0	12	0	0	0	0
a_35_N__a_8_SD	0	0	0	0	0	0	0	0
**a_36_N__a_201_O4**	**0**	**0**	**0**	**0**	**1**	**2**	**0**	**2**
**a_36_N__a_201_O5**	**0**	**0**	**0**	**0**	**0**	**1**	**0**	**0**
a_36_N__a_31_O	0	0	0	0	2	0	0	0
a_36_N__a_34_O	0	0	0	0	0	0	0	0
a_36_N__a_8_SD	36	47	38	57	14	18	39	27
a_37_N__a_35_O	0	0	0	0	0	0	0	0
a_37_N__a_51_OE1	0	0	0	0	0	0	0	0
a_37_N__a_52_O	211	193	161	144	179	165	216	176
a_38_N__a_6_O	242	244	246	240	166	184	220	194
a_38_OG1__a_123_OG	0	0	0	0	0	0	0	0
**a_38_OG1__a_201_O4**	**0**	**0**	**0**	**0**	**3**	**0**	**0**	**1**
a_38_OG1__a_36_O	0	0	1	0	0	0	0	0
a_38_OG1__a_37_O	0	0	0	0	0	0	1	0
a_38_OG1__a_38_O	0	0	0	0	0	0	0	0
a_38_OG1__a_39_O	0	0	0	0	0	3	0	0
a_38_OG1__a_50_O	0	25	36	34	124	128	149	86
a_38_OG1__a_51_OE1	133	21	57	8	10	6	2	1
a_38_OG1__a_6_O	3	10	15	16	4	20	1	16
a_38_OG1__a_8_SD	0	0	1	0	0	0	0	0
a_39_N__a_37_O	0	0	0	0	0	0	0	0
a_39_N__a_38_OG1	0	0	0	0	0	0	0	0
a_39_N__a_39_OE1	0	0	0	0	0	0	0	0
a_39_N__a_39_OE2	0	0	0	0	0	0	0	0
a_39_N__a_50_O	245	226	221	231	203	196	174	192
a_39_N__a_50_OG	0	1	0	1	1	2	0	1
a_3_N__a_1_O	0	0	0	0	0	0	0	0
a_3_OG1__a_1_O	0	0	0	3	1	11	0	7
a_3_OG1__a_2_O	0	0	0	0	1	0	0	0
a_3_OG1__a_3_O	0	0	0	0	0	0	0	0
a_3_OG1__a_40_O	20	8	39	17	0	3	17	1
a_3_OG1__a_41_OE1	9	13	8	3	14	13	8	1

a_3_OG1__a_42_O	0	0	0	0	20	8	4	10
a_3_OG1__a_42_OE1	1	1	3	0	0	0	0	0
a_40_N__a_4_O	241	230	207	221	232	222	217	221
a_41_NE2__a_39_OE1	0	2	1	8	1	2	5	2
a_41_NE2__a_39_OE2	4	3	2	7	0	1	5	3
a_41_NE2__a_3_OG1	0	0	0	0	3	0	0	0
a_41_NE2__a_40_O	4	3	5	7	5	11	2	13
a_41_NE2__a_41_O	0	0	0	1	0	0	0	0
a_41_NE2__a_42_O	5	8	5	4	4	0	2	0
a_41_NE2__a_42_OE1	0	0	0	0	0	0	0	0
a_41_NE2__a_43_OD1	0	0	2	1	0	1	0	2
a_41_NE2__a_43_OD2	1	0	0	0	0	0	0	0
a_41_NE2__a_48_O	5	4	6	4	0	6	7	1
a_41_NE2__a_48_OG1	9	17	7	13	0	1	1	2
a_41_NE2__a_50_OG	0	0	0	1	0	0	0	0
a_41_N__a_41_OE1	0	0	0	0	0	0	0	0
a_41_N__a_48_O	210	215	221	203	220	235	220	231
a_41_N__a_48_OG1	0	0	0	0	0	0	0	0
a_42_NE2__a_1_O	0	0	7	0	0	0	0	0
a_42_NE2__a_2_O	3	0	6	0	0	0	0	0
a_42_NE2__a_3_OG1	0	0	0	0	0	0	0	0
a_42_NE2__a_41_O	0	0	0	0	0	0	0	0
a_42_NE2__a_42_O	8	0	8	1	0	0	0	0
a_42_NE2__a_43_O	0	0	0	0	0	0	0	0
a_42_NE2__a_44_O	68	85	79	95	126	129	109	133
a_42_NE2__a_45_O	0	1	0	6	1	1	0	0
a_42_NE2__a_46_O	6	7	1	6	15	12	16	4
a_42_NE2__a_85_SD	0	0	1	0	0	3	1	1
a_42_N__a_2_O	0	1	27	13	19	35	29	32
a_42_N__a_3_OG1	1	0	2	3	52	17	7	20
a_42_N__a_40_O	0	0	0	0	0	0	0	0
a_42_N__a_41_OE1	15	16	16	7	10	3	15	5
a_42_N__a_42_OE1	0	2	1	0	0	0	0	0
a_43_N__a_41_O	0	0	0	0	0	0	0	0
a_43_N__a_44_O	0	0	0	0	0	0	0	0
a_43_N__a_46_O	211	233	225	194	95	73	89	116
a_44_N__a_42_O	0	0	0	0	0	0	0	0
a_44_N__a_42_OE1	4	2	3	0	0	0	0	1
a_44_N__a_43_OD1	0	0	0	0	0	0	0	0
a_44_N__a_43_OD2	0	0	0	0	0	0	0	0
a_44_N__a_46_O	0	0	0	4	0	0	0	1
a_45_NE2__a_43_OD2	0	0	0	0	0	0	0	3
a_45_NE2__a_44_O	0	0	3	1	0	0	0	3
a_45_NE2__a_45_O	1	3	2	2	1	1	0	0
a_45_NE2__a_46_OD1	0	0	2	0	0	0	0	0
a_45_NE2__a_46_OD2	0	0	0	0	0	0	1	0
a_45_NE2__a_64_OG1	0	0	0	0	0	0	0	1
a_45_NE2__a_65_O	51	38	57	24	98	92	79	57
a_45_NE2__a_85_SD	0	0	0	0	0	1	2	1
a_45_N__a_43_O	0	0	0	0	0	0	0	0

a_45_N__a_43_OD1	0	0	0	1	0	0	0	1
a_45_N__a_43_OD2	0	0	0	0	0	0	0	4
a_45_N__a_45_OE1	0	0	0	0	0	0	0	0
a_46_N__a_43_O	0	1	3	2	0	1	4	1
a_46_N__a_43_OD1	0	0	0	1	0	0	0	1
a_46_N__a_43_OD2	0	0	0	0	0	0	0	4
a_46_N__a_44_O	0	0	0	0	0	0	0	0
a_46_N__a_46_OD1	0	0	0	0	0	0	0	0
a_46_N__a_46_OD2	0	0	0	0	0	0	0	0
a_47_N__a_45_O	0	0	0	0	0	0	0	0
a_47_N__a_46_OD2	0	0	0	0	0	0	0	0
a_47_N__a_63_O	246	246	246	240	238	246	244	242
a_48_N__a_41_O	241	240	220	198	237	226	220	202
a_48_N__a_41_OE1	0	0	0	0	0	0	0	0
a_48_N__a_43_OD2	0	0	0	0	0	0	0	0
a_48_N__a_46_O	0	0	0	0	0	0	0	0
a_48_OG1__a_41_O	14	37	24	28	4	22	19	21
a_48_OG1__a_41_OE1	1	0	0	3	0	0	0	0
a_48_OG1__a_43_OD2	0	0	1	1	0	0	0	0
a_48_OG1__a_46_O	0	0	1	0	0	0	2	2
a_48_OG1__a_46_OD1	0	0	0	0	4	0	0	1
a_48_OG1__a_46_OD2	0	0	0	0	0	0	0	3
a_48_OG1__a_47_O	0	0	0	0	0	0	0	0
a_48_OG1__a_48_O	0	0	0	0	0	0	0	0
a_48_OG1__a_49_O	0	0	0	0	0	0	0	0
a_48_OG1__a_50_OG	0	0	0	0	0	0	0	0
a_48_OG1__a_60_OG1	0	0	0	0	0	0	0	0
a_48_OG1__a_61_O	0	0	12	9	28	51	17	43
a_49_NE1__a_110_OE1	0	0	1	0	0	0	0	0
a_49_NE1__a_110_OE2	0	1	0	0	0	0	0	0
**a_49_NE1__a_201_O**	**0**	**0**	**0**	**0**	**0**	**0**	**0**	**0**
**a_49_NE1__a_201_O2**	**0**	**0**	**0**	**0**	**0**	**13**	**27**	**21**
**a_49_NE1__a_201_O4**	**0**	**0**	**0**	**0**	**4**	**2**	**0**	**0**
**a_49_NE1__a_201_O5**	**0**	**0**	**0**	**0**	**2**	**3**	**0**	**0**
**a_49_NE1__a_202_O7**	**0**	**0**	**0**	**0**	**0**	**0**	**0**	**1**
a_49_NE1__a_38_OG1	3	9	20	22	5	1	35	17
a_49_NE1__a_51_OE1	17	73	13	46	48	12	7	16
a_49_NE1__a_59_SD	0	0	1	0	0	0	0	0
a_49_NE1__a_61_OD1	0	0	0	0	8	14	19	11
a_49_NE1__a_73_O	0	0	0	0	0	0	0	0
a_49_NE1__a_74_SD	0	0	0	0	7	1	0	1
a_49_N__a_48_OG1	0	0	0	0	0	0	0	0
a_49_N__a_60_OG1	0	0	0	1	0	0	0	0
a_49_N__a_61_O	234	233	223	222	230	201	227	218
a_49_N__a_61_OD1	0	0	0	0	0	1	0	0
a_4_N__a_3_OG1	0	0	0	0	0	0	0	0
a_4_N__a_40_O	204	206	145	186	211	126	143	168
a_4_N__a_41_OE1	0	0	0	1	0	1	0	1
a_50_N__a_38_OG1	0	0	0	0	0	2	0	0
a_50_N__a_39_O	238	232	228	223	207	209	195	212

a_50_N__a_41_OE1	0	0	0	0	0	0	0	0
a_50_OG__a_39_O	15	23	53	43	33	20	17	23
a_50_OG__a_39_OE1	0	0	0	1	1	0	0	0
a_50_OG__a_39_OE2	0	0	0	1	0	0	0	0
a_50_OG__a_41_OE1	2	0	2	3	0	0	1	8
a_50_OG__a_48_OG1	0	0	2	0	0	0	0	0
a_50_OG__a_49_O	0	0	0	0	0	0	0	0
a_50_OG__a_50_O	0	0	0	0	0	0	0	0
a_50_OG__a_52_NE2	0	0	0	0	2	2	1	8
a_50_OG__a_58_O	0	0	0	0	0	0	0	0
a_50_OG__a_58_OG1	0	2	0	0	1	0	2	0
a_50_OG__a_59_O	2	9	3	7	20	52	29	18
a_50_OG__a_60_OG1	110	97	48	26	19	13	1	0
a_51_NE2__a_110_OE1	0	0	0	0	0	0	0	0
**a_51_NE2__a_201_N**	**0**	**0**	**0**	**0**	**0**	**0**	**0**	**1**
**a_51_NE2__a_201_O2**	**0**	**0**	**0**	**0**	**46**	**25**	**92**	**44**
**a_51_NE2__a_201_O4**	**0**	**0**	**0**	**0**	**5**	**0**	**0**	**1**
**a_51_NE2__a_201_O5**	**0**	**0**	**0**	**0**	**3**	**0**	**0**	**3**
a_51_NE2__a_37_O	1	8	4	1	0	0	0	0
a_51_NE2__a_38_OG1	2	11	43	48	8	4	1	15
a_51_NE2__a_50_O	2	2	0	0	0	0	0	0
a_51_NE2__a_51_O	0	0	0	0	0	0	0	0
a_51_NE2__a_52_O	0	0	0	0	0	0	0	3
a_51_NE2__a_53_OH	6	31	2	19	0	0	0	6
a_51_NE2__a_59_O	2	7	2	2	0	1	0	0
a_51_NE2__a_59_SD	0	2	0	1	0	1	0	1
a_51_NE2__a_72_OE1	0	1	0	0	0	0	0	0
a_51_NE2__a_73_O	3	0	5	0	0	0	0	0
a_51_NE2__a_74_SD	0	0	0	0	1	2	0	0
a_51_N__a_50_OG	0	0	0	0	0	0	0	0
a_51_N__a_51_OE1	0	0	0	0	0	2	0	0
a_51_N__a_58_OG1	0	0	2	0	0	0	0	0
a_51_N__a_59_O	243	237	236	230	229	186	205	220
a_52_ND1__a_37_O	0	0	0	0	2	4	3	4
a_52_ND1__a_39_OE1	0	0	0	0	8	8	8	0
a_52_ND1__a_50_O	0	0	0	0	2	1	3	0
a_52_ND1__a_50_OG	0	0	0	0	12	3	18	2
a_52_ND1__a_51_O	0	0	0	0	0	0	0	0
a_52_ND1__a_52_O	0	0	0	0	0	0	0	0
a_52_ND1__a_53_O	0	0	0	0	0	0	0	2
a_52_ND1__a_54_OG	0	0	0	0	0	0	1	0
a_52_ND1__a_57_O	0	0	0	0	0	4	0	20
a_52_ND1__a_58_OG1	0	0	0	0	23	19	4	46
a_52_NE2__a_39_OE1	56	12	26	10	0	0	0	0
a_52_NE2__a_39_OE2	40	8	21	2	0	0	0	0
a_52_NE2__a_50_OG	1	0	0	0	0	0	0	0
a_52_NE2__a_53_O	0	0	0	0	0	0	0	0
a_52_NE2__a_54_O	0	0	0	0	0	0	0	0
a_52_NE2__a_55_O	4	0	0	3	0	0	0	0
a_52_NE2__a_56_O	0	0	0	0	0	0	0	0

a_52_NE2__a_58_OG1	0	0	0	0	0	0	0	0
a_52_N__a_37_O	246	241	231	223	191	187	217	209
a_52_N__a_50_O	0	0	0	0	0	0	0	0
a_52_N__a_51_OE1	0	0	0	0	0	0	0	0
a_52_N__a_52_ND1	0	0	0	0	0	0	0	0
a_53_N__a_51_O	0	0	0	0	0	0	0	0
a_53_N__a_52_ND1	0	0	0	0	0	0	0	0
a_53_N__a_54_O	0	0	0	0	0	0	0	0
a_53_N__a_57_ND1	0	0	0	0	0	0	0	0
a_53_N__a_57_O	71	33	87	156	88	47	55	75
a_53_N__a_58_OG1	0	1	0	2	0	0	1	0
a_53_OH__a_14_OH	0	0	1	10	0	0	0	0
**a_53_OH__a_201_O2**	**0**	**0**	**0**	**0**	**0**	**0**	**0**	**1**
**a_53_OH__a_201_O4**	**0**	**0**	**0**	**0**	**0**	**0**	**0**	**3**
**a_53_OH__a_201_O5**	**0**	**0**	**0**	**0**	**0**	**0**	**0**	**3**
a_53_OH__a_26_OD1	0	0	0	0	0	0	3	4
a_53_OH__a_26_OD2	0	0	0	0	0	0	2	7
a_53_OH__a_27_O	0	0	3	2	0	0	0	1
a_53_OH__a_30_O	0	3	0	0	0	0	0	0
a_53_OH__a_31_O	0	0	1	0	0	0	0	0
a_53_OH__a_51_OE1	29	20	88	45	6	0	0	7
a_53_OH__a_57_ND1	3	9	10	0	0	0	2	0
a_53_OH__a_57_NE2	0	0	0	0	0	0	0	0
a_53_OH__a_58_O	0	0	1	0	0	0	0	0
a_53_OH__a_59_SD	0	3	4	0	0	0	0	0
a_53_OH__a_73_O	0	0	0	21	0	0	0	0
a_53_OH__a_74_O	0	0	0	3	0	0	0	0
a_53_OH__a_74_SD	5	0	2	3	0	0	1	0
a_53_OH__a_75_O	1	0	1	0	0	0	0	0
a_54_N__a_30_O	0	0	0	0	0	0	0	0
a_54_N__a_33_OD1	0	0	0	4	0	0	0	0
a_54_N__a_34_O	0	0	0	0	4	1	1	0
a_54_N__a_35_O	0	0	0	16	0	0	0	0
a_54_N__a_52_ND1	3	3	14	2	0	0	0	0
a_54_N__a_52_O	0	0	0	0	0	0	0	0
a_54_N__a_57_O	49	16	28	18	0	0	0	0
a_54_OG__a_30_O	0	2	0	33	0	7	0	3
a_54_OG__a_31_O	0	0	0	0	0	0	0	0
a_54_OG__a_33_OD1	0	4	1	9	0	1	0	2
a_54_OG__a_34_O	0	0	0	7	13	6	2	0
a_54_OG__a_35_O	0	1	8	9	0	0	0	0
a_54_OG__a_53_O	5	3	3	3	0	0	0	0
a_54_OG__a_54_O	0	0	0	0	0	0	0	0
a_54_OG__a_55_O	0	0	0	1	0	0	0	0
a_54_OG__a_57_ND1	0	0	0	0	0	0	0	0
a_54_OG__a_57_O	1	3	4	0	0	0	0	0
a_55_N__a_52_ND1	0	1	0	0	0	0	0	0
a_55_N__a_53_O	0	0	0	0	0	0	0	0
a_55_N__a_54_OG	0	0	0	0	0	0	0	0
a_55_N__a_57_O	19	1	16	7	0	0	0	0

a_55_N__a_58_OG1	1	0	1	0	0	0	0	0
a_56_N__a_53_O	0	0	0	47	38	36	23	60
a_56_N__a_54_O	0	0	0	0	0	0	0	0
a_56_N__a_54_OG	86	47	69	13	0	0	0	0
a_56_N__a_57_O	0	0	0	0	0	0	0	0
a_57_NE2__a_14_OH	0	0	0	1	0	0	0	0
**a_57_NE2__a_201_O5**	**0**	**0**	**0**	**0**	**0**	**0**	**0**	**0**
a_57_NE2__a_27_O	0	0	0	0	0	0	0	0
a_57_NE2__a_30_O	1	0	0	0	0	0	0	0
a_57_NE2__a_51_OE1	0	0	0	0	22	20	35	12
a_57_NE2__a_53_OH	0	0	1	0	0	0	0	0
a_57_NE2__a_59_SD	0	0	0	0	0	0	0	0
a_57_NE2__a_73_O	0	0	0	11	0	0	0	0
a_57_NE2__a_74_O	0	0	0	39	0	0	0	1
a_57_NE2__a_74_SD	0	0	1	5	1	0	0	0
a_57_NE2__a_75_O	0	8	10	1	0	0	0	0
a_57_N__a_30_O	0	0	0	1	0	0	0	0
a_57_N__a_53_O	0	0	0	13	17	8	14	9
a_57_N__a_54_O	12	3	10	23	0	0	0	0
a_57_N__a_54_OG	96	45	84	12	0	0	0	0
a_57_N__a_55_O	0	0	0	0	0	0	0	0
a_58_N__a_56_O	0	0	0	0	0	0	0	0
a_58_N__a_57_ND1	0	0	0	0	0	0	0	0
a_58_N__a_58_OG1	2	2	14	18	8	0	0	6
a_58_OG1__a_50_OG	0	0	1	0	0	0	0	0
a_58_OG1__a_51_O	2	55	14	16	6	11	5	19
a_58_OG1__a_52_ND1	56	17	38	25	0	0	0	0
a_58_OG1__a_52_NE2	0	0	0	0	0	8	0	0
a_58_OG1__a_53_O	0	0	0	0	0	0	0	0
a_58_OG1__a_54_O	0	2	0	0	0	0	0	0
a_58_OG1__a_56_O	5	6	0	4	0	0	1	5
a_58_OG1__a_57_O	0	0	0	0	1	1	0	0
a_58_OG1__a_58_O	0	0	0	0	0	0	0	0
a_58_OG1__a_59_O	0	0	0	0	0	0	0	0
a_58_OG1__a_60_OG1	0	0	0	0	0	0	0	0
a_59_N__a_51_O	200	147	189	213	218	232	224	219
a_59_N__a_57_O	0	0	0	0	0	0	0	0
a_59_N__a_58_OG1	3	41	24	10	10	10	6	13
a_5_NZ__a_126_O	0	0	1	0	0	0	0	0
a_5_NZ__a_38_O	0	0	0	0	0	1	0	0
a_5_NZ__a_39_OE1	8	16	17	18	19	27	18	10
a_5_NZ__a_39_OE2	27	19	29	35	21	10	12	6
a_5_NZ__a_7_OE1	0	0	0	0	2	0	2	9
a_5_NZ__a_7_OE2	0	0	0	0	8	1	14	13
a_5_N__a_126_O	1	0	1	1	1	11	7	6
a_60_N__a_58_O	1	1	3	1	2	0	3	1
a_60_N__a_59_SD	0	2	1	8	2	3	6	11
a_60_N__a_60_O	11	17	15	31	14	11	13	17
a_60_N__a_60_OG1	1	1	1	3	9	7	24	11
a_60_N__a_72_OE1	8	23	12	26	0	0	0	0

a_60_N__a_74_O	0	0	0	0	0	2	3	1
a_60_OG1__a_48_OG1	0	0	0	1	0	0	0	0
a_60_OG1__a_49_O	8	9	17	27	0	4	0	24
a_60_OG1__a_50_OG	21	8	16	6	0	2	1	3
a_60_OG1__a_58_O	0	0	1	1	0	0	0	0
a_60_OG1__a_58_OG1	2	2	4	0	0	0	0	0
a_60_OG1__a_59_O	0	0	0	0	0	0	0	0
a_60_OG1__a_60_O	0	0	0	0	0	0	0	0
a_60_OG1__a_61_O	0	0	0	0	0	0	0	0
**a_61_ND2__a_201_O4**	**0**	**0**	**0**	**0**	**0**	**2**	**0**	**1**
**a_61_ND2__a_201_O5**	**0**	**0**	**0**	**0**	**0**	**3**	**0**	**2**
**a_61_ND2__a_202_O3**	**0**	**0**	**0**	**0**	**15**	**0**	**0**	**0**
**a_61_ND2__a_202_O7**	**0**	**0**	**0**	**0**	**8**	**17**	**8**	**21**
a_61_ND2__a_49_O	39	13	22	5	1	2	1	0
a_61_ND2__a_51_OE1	2	2	3	31	0	1	4	3
a_61_ND2__a_59_O	0	0	1	1	1	0	2	0
a_61_ND2__a_59_SD	0	0	0	0	8	0	1	0
a_61_ND2__a_60_O	0	0	0	0	0	0	1	0
a_61_ND2__a_61_O	0	0	0	0	0	0	0	0
a_61_ND2__a_62_O	1	3	3	2	0	1	5	0
a_61_ND2__a_69_OG	0	1	0	3	0	0	0	0
a_61_ND2__a_70_O	17	58	37	30	0	0	0	7
a_61_ND2__a_70_OD1	0	0	0	0	0	0	0	0
a_61_ND2__a_72_O	0	0	0	0	22	13	2	7
a_61_ND2__a_72_OE1	11	3	5	12	1	10	4	1
a_61_ND2__a_73_O	44	9	98	67	5	0	0	0
a_61_ND2__a_74_O	0	0	0	0	0	0	0	0
a_61_ND2__a_74_SD	0	0	0	0	43	0	0	7
a_61_N__a_49_O	205	218	211	215	208	190	201	202
a_61_N__a_59_O	0	0	0	0	0	0	0	0
a_61_N__a_60_OG1	0	0	0	0	0	0	0	0
a_61_N__a_61_OD1	0	0	0	0	0	0	0	0
a_62_NZ__a_43_O	0	5	0	12	0	0	0	0
a_62_NZ__a_43_OD1	31	54	20	46	7	3	3	9
a_62_NZ__a_43_OD2	41	44	30	47	3	7	1	14
a_62_NZ__a_46_OD1	16	11	19	14	8	12	18	19
a_62_NZ__a_46_OD2	8	4	25	23	7	11	22	16
a_62_NZ__a_47_O	0	0	0	1	0	0	0	0
a_62_NZ__a_48_OG1	0	7	28	3	15	6	10	17
a_62_NZ__a_60_OG1	0	0	0	0	8	8	0	8
a_62_NZ__a_63_O	0	0	0	0	0	0	0	0
a_62_NZ__a_64_OG1	0	0	0	0	0	0	0	2
a_62_NZ__a_70_OD1	0	0	0	0	0	0	0	1
a_62_NZ__a_72_OE1	0	0	0	0	1	0	0	0
a_62_N__a_60_O	0	0	0	0	0	0	0	0
a_62_N__a_61_OD1	3	0	0	1	6	13	12	8
a_62_N__a_70_O	0	0	0	3	0	0	0	0
a_62_N__a_72_O	0	0	0	0	0	0	0	3
a_62_N__a_72_OE1	0	0	0	1	26	22	25	19
a_63_N__a_47_O	234	226	218	227	200	200	206	199

a_63_N__a_61_O	0	0	0	0	0	0	0	0
a_64_N__a_46_OD1	0	0	0	0	0	0	0	0
a_64_N__a_46_OD2	0	0	0	0	0	0	0	0
a_64_N__a_69_OG	181	191	175	190	0	0	0	0
a_64_N__a_70_O	0	0	0	0	6	0	6	7
a_64_N__a_70_OD1	0	0	0	0	173	216	208	207
a_64_OG1__a_45_O	3	1	14	4	7	2	6	16
a_64_OG1__a_46_OD1	93	94	80	78	100	91	100	80
a_64_OG1__a_46_OD2	90	71	67	87	70	97	84	81
a_64_OG1__a_63_O	0	0	0	0	0	0	1	0
a_64_OG1__a_64_O	0	0	0	0	0	0	0	0
a_64_OG1__a_67_O	0	5	1	4	4	0	0	0
a_64_OG1__a_68_O	2	0	0	0	0	0	0	0
a_64_OG1__a_69_OG	2	3	1	5	0	0	0	0
a_64_OG1__a_70_OD1	0	0	0	0	9	5	5	7
a_65_N__a_45_O	116	102	153	95	210	213	184	181
a_65_N__a_63_O	0	0	0	0	0	0	0	0
a_65_N__a_64_OG1	0	0	0	0	0	0	0	0
a_66_N__a_64_O	0	0	0	0	0	0	0	0
a_66_N__a_82_OG1	0	0	0	0	2	0	0	0
a_66_N__a_83_O	225	197	198	158	171	168	172	127
a_67_NZ__a_45_OE1	0	0	0	0	2	0	0	1
a_67_NZ__a_64_OG1	15	5	1	9	16	14	0	3
a_67_NZ__a_65_O	0	0	0	0	1	1	0	0
a_67_NZ__a_67_O	0	0	0	0	0	0	0	0
a_67_NZ__a_68_O	15	5	8	6	1	0	0	1
a_67_NZ__a_68_OE1	21	8	6	12	3	3	1	0
a_67_NZ__a_68_OE2	17	6	2	7	2	0	1	2
a_67_N__a_64_O	10	13	17	7	11	7	7	6
a_67_N__a_65_O	0	0	0	0	0	0	0	0
a_67_N__a_83_O	4	1	1	4	0	0	0	0
a_68_N__a_68_OE1	13	2	7	12	2	5	2	18
a_68_N__a_68_OE2	10	3	6	5	5	5	5	15
a_68_N__a_81_O	0	0	0	0	0	0	0	0
a_69_N__a_67_O	0	0	0	0	0	0	0	0
a_69_N__a_68_OE1	0	0	0	0	0	0	0	1
a_69_N__a_68_OE2	0	0	0	0	0	0	0	0
a_69_N__a_79_O	0	0	0	0	8	11	5	28
a_69_N__a_80_O	0	0	0	0	0	0	0	0
a_69_N__a_81_O	232	220	226	236	109	72	137	26
a_69_N__a_82_OG1	0	0	0	0	0	0	0	1
a_69_OG__a_61_OD1	0	0	0	2	0	0	0	0
a_69_OG__a_62_O	5	1	2	1	0	0	0	0
a_69_OG__a_64_O	3	0	3	0	0	0	0	0
a_69_OG__a_64_OG1	0	1	1	0	0	0	0	0
a_69_OG__a_67_O	18	41	29	19	0	0	0	0
a_69_OG__a_68_O	3	1	0	0	1	3	5	13
a_69_OG__a_68_OE1	0	0	0	0	19	7	23	23
a_69_OG__a_68_OE2	0	0	0	0	41	11	10	37
a_69_OG__a_69_O	0	0	0	0	0	0	0	0

a_69_OG__a_70_O	0	4	0	0	0	0	0	0
a_69_OG__a_78_OG1	0	0	0	0	2	6	21	4
a_69_OG__a_79_O	0	0	0	0	12	8	5	0
a_69_OG__a_81_O	6	0	1	0	0	0	0	0
a_6_N__a_38_O	247	247	247	244	211	228	232	241
a_6_N__a_38_OG1	0	0	0	0	1	0	0	0
a_6_N__a_4_O	0	0	0	0	0	0	0	0
a_70_ND2__a_61_OD1	0	7	1	0	0	0	0	0
a_70_ND2__a_62_O	0	0	0	0	3	0	0	0
a_70_ND2__a_64_OG1	0	0	0	0	10	0	1	1
a_70_ND2__a_67_O	0	0	0	0	0	0	0	3
a_70_ND2__a_68_O	0	0	0	0	10	10	14	16
a_70_ND2__a_68_OE1	0	0	0	1	0	0	0	0
a_70_ND2__a_69_O	0	0	0	0	0	0	0	0
a_70_ND2__a_70_O	0	0	0	0	1	0	0	0
a_70_ND2__a_72_OE1	0	0	10	0	0	0	0	0
a_70_ND2__a_78_OG1	4	12	17	11	0	0	0	0
a_70_N__a_62_O	0	0	0	3	0	0	0	0
a_70_N__a_67_O	0	0	0	0	3	0	1	0
a_70_N__a_68_O	0	0	0	0	0	0	0	0
a_70_N__a_69_OG	0	0	0	0	0	0	0	0
a_70_N__a_70_OD1	0	0	0	0	0	0	0	0
a_70_N__a_79_O	0	0	0	0	0	0	0	0
a_70_N__a_81_O	0	0	0	0	14	0	8	14
a_71_N__a_67_O	0	0	0	0	0	0	0	0
a_71_N__a_69_O	0	0	0	0	0	0	0	0
a_71_N__a_70_OD1	0	0	0	0	0	0	0	0
a_71_N__a_79_O	230	228	226	220	0	0	0	0
a_71_N__a_81_O	0	0	0	0	0	0	1	2
a_72_NE2__a_51_OE1	0	1	1	0	0	0	0	0
a_72_NE2__a_59_SD	0	0	0	0	0	0	0	0
a_72_NE2__a_60_O	4	12	14	10	8	8	8	8
a_72_NE2__a_61_OD1	7	9	4	9	9	6	0	7
a_72_NE2__a_62_O	0	0	0	0	4	2	2	2
a_72_NE2__a_70_O	0	0	0	0	1	3	3	2
a_72_NE2__a_72_O	0	0	0	0	0	1	0	0
a_72_NE2__a_73_O	0	0	6	0	1	1	1	2
a_72_NE2__a_74_O	0	0	0	1	0	0	0	0
a_72_NE2__a_75_O	63	5	4	38	0	0	0	0
a_72_NE2__a_76_O	6	2	6	2	24	16	19	9
a_72_NE2__a_77_O	1	0	1	0	0	0	1	1
a_72_NE2__a_78_OG1	10	1	9	2	1	0	0	1
a_72_N__a_61_OD1	121	145	142	115	0	0	0	1
a_72_N__a_62_O	0	0	0	0	5	0	0	3
a_72_N__a_70_O	0	0	0	0	0	0	0	0
a_72_N__a_72_OE1	9	2	6	5	0	1	0	0
a_73_N__a_59_SD	0	0	0	10	0	0	0	0
a_73_N__a_61_OD1	134	40	155	139	0	0	0	0
a_73_N__a_71_O	0	5	19	1	0	0	0	0
a_73_N__a_72_OE1	0	0	3	0	0	0	0	2

a_73_N__a_73_O	0	0	0	0	0	0	0	0
a_73_N__a_73_OG1	0	0	0	2	60	48	51	64
a_73_N__a_76_O	0	0	0	0	0	0	0	0
a_73_N__a_77_O	0	0	0	0	248	247	248	247
**a_73_OG1__a_202_N25**	**0**	**0**	**0**	**0**	**1**	**0**	**0**	**0**
**a_73_OG1__a_202_OT1**	**0**	**0**	**0**	**0**	**23**	**24**	**35**	**29**
**a_73_OG1__a_202_OT2**	**0**	**0**	**0**	**0**	**40**	**27**	**26**	**24**
a_73_OG1__a_71_O	0	0	0	0	0	0	0	0
a_73_OG1__a_72_O	247	237	228	243	0	0	0	0
a_73_OG1__a_72_OE1	0	0	0	0	0	0	0	0
a_73_OG1__a_73_O	0	0	0	1	1	0	0	3
a_73_OG1__a_74_O	1	0	1	0	0	0	0	1
a_73_OG1__a_75_O	0	0	0	7	0	0	0	0
a_73_OG1__a_76_O	0	0	0	0	0	0	0	0
a_73_OG1__a_77_O	25	171	154	40	162	193	187	171
a_73_OG1__a_78_O	0	0	0	0	1	0	0	0
a_73_OG1__a_97_OH	0	0	0	0	0	0	0	0
**a_74_N__a_202_N25**	**0**	**0**	**0**	**0**	**0**	**1**	**2**	**1**
**a_74_N__a_202_O24**	**0**	**0**	**0**	**0**	**64**	**114**	**118**	**117**
**a_74_N__a_202_OT1**	**0**	**0**	**0**	**0**	**12**	**18**	**6**	**13**
**a_74_N__a_202_OT2**	**0**	**0**	**0**	**0**	**23**	**15**	**5**	**16**
a_74_N__a_72_O	0	0	0	2	0	0	0	0
a_74_N__a_73_OG1	0	0	0	0	0	0	0	0
a_74_N__a_74_SD	0	0	0	2	2	17	6	8
a_74_N__a_75_O	0	0	0	4	0	0	0	0
a_74_N__a_97_OH	0	1	0	8	0	0	0	0
**a_75_N__a_202_O24**	**0**	**0**	**0**	**0**	**1**	**2**	**0**	**0**
**a_75_N__a_202_OT1**	**0**	**0**	**0**	**0**	**11**	**3**	**4**	**9**
**a_75_N__a_202_OT2**	**0**	**0**	**0**	**0**	**3**	**3**	**4**	**2**
a_75_N__a_53_OH	1	1	32	0	0	0	0	0
a_75_N__a_59_SD	1	0	3	0	0	0	0	0
a_75_N__a_72_O	0	0	0	101	0	0	0	0
a_75_N__a_72_OE1	0	0	0	4	0	0	0	0
a_75_N__a_73_O	0	0	0	0	0	0	0	0
a_75_N__a_73_OG1	0	0	0	2	3	2	1	2
a_75_N__a_74_SD	1	0	1	1	0	1	0	0
a_75_N__a_75_O	0	0	0	2	0	0	0	0
a_76_N__a_72_O	0	0	9	6	0	0	0	0
a_76_N__a_72_OE1	0	0	0	4	0	0	0	0
a_76_N__a_73_O	0	0	0	0	94	102	98	86
a_76_N__a_73_OG1	0	0	0	0	22	14	13	30
a_76_N__a_74_O	0	0	0	0	0	0	0	0
**a_77_NZ__a_202_N25**	**0**	**0**	**0**	**0**	**0**	**0**	**0**	**0**
**a_77_NZ__a_202_O24**	**0**	**0**	**0**	**0**	**2**	**0**	**0**	**1**
**a_77_NZ__a_202_OT1**	**0**	**0**	**0**	**0**	**0**	**1**	**5**	**2**
**a_77_NZ__a_202_OT2**	**0**	**0**	**0**	**0**	**1**	**1**	**5**	**5**
a_77_NZ__a_21_O	0	5	37	7	0	0	0	0
a_77_NZ__a_22_O	5	1	3	1	0	0	0	0
a_77_NZ__a_26_OD2	0	0	0	0	0	0	0	0
a_77_NZ__a_73_OG1	0	0	0	0	0	1	0	0

a_77_NZ__a_75_O	0	0	0	0	5	1	2	5
a_77_NZ__a_78_O	6	3	0	1	0	1	0	2
**a_77_N__a_202_OT1**	**0**	**0**	**0**	**0**	**1**	**0**	**0**	**1**
a_77_N__a_72_O	0	9	37	9	0	0	0	0
a_77_N__a_73_OG1	0	0	0	0	60	32	48	66
a_77_N__a_75_O	1	11	2	22	39	54	45	38
a_78_N__a_72_OE1	0	0	0	0	0	0	0	0
a_78_N__a_76_O	0	0	0	0	0	0	0	0
a_78_OG1__a_69_O	0	0	0	0	0	1	0	4
a_78_OG1__a_69_OG	0	0	0	0	0	0	0	2
a_78_OG1__a_70_OD1	4	1	0	4	0	0	0	0
a_78_OG1__a_71_O	0	0	4	0	0	0	9	0
a_78_OG1__a_72_OE1	7	2	8	16	1	3	5	5
a_78_OG1__a_76_O	1	3	3	1	0	0	0	0
a_78_OG1__a_77_O	0	0	0	17	0	0	0	0
a_78_OG1__a_78_O	0	0	0	0	0	0	0	0
a_79_N__a_70_OD1	0	0	1	0	0	0	0	0
a_79_N__a_71_O	239	214	217	238	111	146	125	130
a_79_N__a_77_O	0	0	0	0	0	0	0	0
a_79_N__a_78_OG1	0	0	0	0	0	0	0	0
a_7_N__a_123_OG	0	0	0	0	0	0	0	0
a_7_N__a_124_O	243	239	244	237	190	209	205	183
a_80_NZ__a_68_O	0	0	0	0	0	0	0	0
a_80_NZ__a_68_OE1	22	41	47	19	50	28	38	29
a_80_NZ__a_68_OE2	15	26	27	13	42	27	41	41
a_80_NZ__a_69_OG	0	0	0	0	23	37	39	54
a_80_NZ__a_70_OD1	0	2	8	7	0	0	0	0
a_80_NZ__a_78_O	0	0	0	0	0	0	0	0
a_80_NZ__a_78_OG1	0	0	0	1	3	9	6	10
**a_80_N__a_202_OT1**	**0**	**0**	**0**	**0**	**1**	**0**	**0**	**0**
a_80_N__a_78_O	0	0	0	0	0	0	0	0
a_81_N__a_68_OE1	0	0	0	0	0	1	0	0
a_81_N__a_68_OE2	0	0	0	0	0	0	0	0
a_81_N__a_69_O	247	247	245	244	0	0	0	0
a_81_N__a_69_OG	0	0	0	0	0	0	0	0
a_81_N__a_79_O	4	2	18	37	215	186	230	221
a_81_N__a_81_O	42	52	39	23	60	62	42	61
a_82_N__a_93_O	59	29	49	63	40	9	35	4
a_82_N__a_93_OD1	0	0	19	14	0	0	0	0
a_82_OG1__a_66_O	0	0	0	0	0	0	0	0
a_82_OG1__a_67_O	2	1	0	0	6	2	3	9
a_82_OG1__a_68_OE1	0	0	0	0	2	8	6	2
a_82_OG1__a_68_OE2	0	0	0	0	4	3	6	0
a_82_OG1__a_80_O	0	0	0	0	0	0	0	0
a_82_OG1__a_81_O	0	0	0	0	0	0	0	0
a_82_OG1__a_82_O	0	0	0	0	0	0	0	0
a_82_OG1__a_83_O	0	0	0	0	1	0	0	0
a_82_OG1__a_84_OE1	4	4	12	6	9	23	48	44
a_82_OG1__a_93_O	4	0	3	3	3	2	3	3
a_82_OG1__a_93_OD1	11	3	13	19	0	0	1	4

a_83_N__a_66_O	0	0	0	1	0	0	0	0
a_83_N__a_67_O	71	55	55	50	4	1	11	2
a_83_N__a_81_O	0	0	0	0	0	0	0	0
a_83_N__a_82_OG1	0	0	0	0	0	0	0	0
a_84_NE2__a_66_O	0	0	0	0	0	0	0	0
a_84_NE2__a_82_O	0	1	0	1	0	8	0	0
a_84_NE2__a_82_OG1	0	0	0	0	0	0	1	5
a_84_NE2__a_83_O	0	0	0	1	0	0	0	0
a_84_NE2__a_84_O	0	0	0	0	0	0	0	0
a_84_NE2__a_85_O	0	1	1	2	1	0	4	1
a_84_NE2__a_86_OE1	4	3	4	1	4	1	0	6
a_84_NE2__a_86_OE2	2	6	1	3	2	2	1	3
a_84_NE2__a_91_O	18	15	33	22	22	48	47	33
a_84_N__a_82_O	0	0	0	0	0	0	0	0
a_84_N__a_84_OE1	0	0	0	0	0	1	0	0
a_84_N__a_91_O	243	230	232	237	210	172	159	188
a_85_N__a_83_O	0	0	0	0	0	0	0	0
a_85_N__a_84_OE1	2	6	4	4	6	2	6	5
a_86_N__a_84_O	0	0	0	0	0	0	0	0
a_86_N__a_85_SD	1	0	0	2	2	0	0	0
a_86_N__a_86_O	1	0	2	0	0	9	0	0
a_86_N__a_86_OE1	0	0	0	0	0	0	0	0
a_86_N__a_89_O	231	204	221	225	235	230	233	230
a_87_N__a_85_O	0	0	0	0	0	0	0	0
a_87_N__a_85_SD	10	29	23	23	1	1	2	3
a_87_N__a_86_OE1	2	5	1	0	0	0	0	0
a_87_N__a_86_OE2	6	0	1	2	0	0	0	0
a_87_N__a_89_O	0	8	2	5	0	0	0	0
a_88_N__a_85_O	0	0	1	0	0	0	0	2
a_88_N__a_85_SD	6	1	6	0	9	6	1	6
a_88_N__a_86_O	41	24	40	32	3	26	23	16
a_88_N__a_86_OE1	0	17	0	2	0	0	0	0
a_88_N__a_86_OE2	0	0	0	2	0	0	0	0
a_88_N__a_89_O	0	0	0	0	0	0	0	0
a_89_NZ__a_100_OG1	0	0	0	0	0	0	0	1
a_89_NZ__a_101_O	0	0	0	0	11	11	49	12
a_89_NZ__a_102_OE1	16	21	17	22	40	65	34	68
a_89_NZ__a_102_OE2	10	28	15	17	43	60	51	59
a_89_NZ__a_86_O	14	0	7	2	18	15	15	13
a_89_NZ__a_86_OE1	0	1	5	5	1	10	4	1
a_89_NZ__a_86_OE2	5	10	19	12	4	2	2	1
a_89_N__a_85_SD	0	1	0	1	0	0	0	0
a_89_N__a_86_O	41	18	41	27	17	29	51	27
a_89_N__a_86_OE1	0	10	0	0	0	0	0	0
a_89_N__a_86_OE2	0	0	0	1	0	0	1	0
a_89_N__a_87_O	0	0	0	0	0	0	0	0
a_8_N__a_36_O	73	159	178	167	104	77	111	84
a_8_N__a_7_OE1	8	0	0	5	11	3	0	0
a_8_N__a_7_OE2	3	0	0	1	6	4	0	0
a_8_N__a_8_SD	1	0	0	4	0	0	0	0

a_90_N__a_101_O	234	239	237	233	50	19	39	20
a_90_N__a_101_OG	0	0	0	2	29	88	17	67
**a_90_N__a_201_O**	**0**	**0**	**0**	**0**	**31**	**14**	**53**	**29**
a_91_N__a_84_O	238	231	230	234	234	224	212	220
a_92_N__a_99_O	222	194	217	199	168	165	141	144
a_92_N__a_99_OE1	0	0	1	0	0	1	26	14
a_93_ND2__a_82_O	0	0	0	0	0	0	0	0
a_93_ND2__a_82_OG1	2	0	3	4	1	1	1	0
a_93_ND2__a_84_OE1	0	0	0	0	11	11	11	14
a_93_ND2__a_92_O	0	0	0	0	0	0	0	0
a_93_ND2__a_93_O	0	1	0	0	0	0	0	0
a_93_ND2__a_94_O	0	0	0	0	0	0	0	0
a_93_ND2__a_97_O	0	0	0	0	1	0	0	0
a_93_ND2__a_98_ND1	28	48	30	13	0	1	0	0
a_93_ND2__a_98_NE2	0	0	0	0	0	0	0	0
a_93_N__a_82_O	103	26	52	97	66	18	45	21
a_93_N__a_82_OG1	0	0	0	0	0	0	0	0
a_93_N__a_91_O	0	0	0	0	0	0	0	0
a_93_N__a_93_OD1	0	0	0	0	0	0	0	0
a_94_N__a_92_O	0	0	0	0	0	0	0	0
a_94_N__a_93_OD1	0	0	0	0	0	0	1	0
a_94_N__a_97_O	221	188	192	205	148	218	161	188
a_96_ND2__a_114_O	0	0	0	0	0	0	3	4
**a_96_ND2__a_202_N25**	**0**	**0**	**0**	**0**	**0**	**0**	**0**	**0**
**a_96_ND2__a_202_O24**	**0**	**0**	**0**	**0**	**0**	**1**	**0**	**0**
a_96_ND2__a_20_O	12	0	19	4	0	3	0	0
a_96_ND2__a_21_O	92	7	8	16	0	0	0	0
a_96_ND2__a_73_OG1	12	10	1	1	0	0	0	0
a_96_ND2__a_74_O	0	8	7	0	0	0	0	0
a_96_ND2__a_74_SD	0	0	0	5	0	0	0	0
a_96_ND2__a_95_O	0	0	0	0	0	0	1	0
a_96_ND2__a_96_O	0	0	1	0	8	10	6	2
a_96_N__a_94_O	0	0	0	0	0	0	0	0
a_96_N__a_96_OD1	0	0	0	0	0	0	0	0
a_96_N__a_98_NE2	0	0	0	0	0	0	5	0
a_97_N__a_94_O	17	16	25	17	114	97	116	89
a_97_N__a_95_O	0	0	0	0	0	0	0	0
a_97_N__a_96_OD1	5	4	1	1	0	0	0	0
a_97_OH__a_112_OG	0	0	7	0	0	2	6	3
a_97_OH__a_119_OH	0	0	0	0	0	0	0	10
**a_97_OH__a_201_O2**	**0**	**0**	**0**	**0**	**0**	**1**	**0**	**0**
**a_97_OH__a_202_O7**	**0**	**0**	**0**	**0**	**12**	**3**	**0**	**6**
a_97_OH__a_21_O	0	0	0	0	0	0	0	0
a_97_OH__a_73_O	0	0	0	0	0	0	0	0
a_97_OH__a_73_OG1	2	0	0	4	0	0	0	0
a_97_OH__a_74_SD	0	0	2	2	0	0	0	0
a_97_OH__a_99_OE1	11	11	12	17	174	135	124	111
a_98_ND1__a_113_O	0	0	0	0	0	0	1	1
a_98_ND1__a_113_OG1	0	0	0	0	4	0	2	0
a_98_ND1__a_92_O	0	0	0	0	112	171	129	137

a_98_ND1__a_93_OD1	0	0	0	0	44	15	13	15
a_98_ND1__a_94_O	0	0	0	0	0	1	0	2
a_98_ND1__a_96_O	0	0	0	0	0	0	0	0
a_98_ND1__a_97_O	0	0	0	0	0	0	0	0
a_98_ND1__a_98_O	0	0	0	0	0	0	0	0
a_98_ND1__a_99_O	0	0	0	0	0	1	1	1
a_98_NE2__a_100_OG1	1	1	1	2	0	0	0	0
a_98_NE2__a_93_OD1	0	0	0	0	0	0	0	0
a_98_N__a_113_O	227	214	206	221	163	170	188	180
a_98_N__a_113_OG1	0	0	0	0	0	0	0	1
a_98_N__a_96_O	0	0	0	0	0	0	0	0
a_99_NE2__a_100_O	1	0	0	0	0	0	0	0
a_99_NE2__a_101_OG	0	1	3	5	11	5	11	2
a_99_NE2__a_110_OE1	12	23	13	13	38	51	116	46
a_99_NE2__a_110_OE2	14	6	18	8	69	36	37	54
a_99_NE2__a_111_O	0	1	0	0	0	0	0	0
a_99_NE2__a_112_OG	43	41	39	44	0	0	0	1
**a_99_NE2__a_201_O**	**0**	**0**	**0**	**0**	**0**	**0**	**1**	**7**
**a_99_NE2__a_201_O1**	**0**	**0**	**0**	**0**	**0**	**0**	**0**	**0**
**a_99_NE2__a_201_O3**	**0**	**0**	**0**	**0**	**0**	**0**	**0**	**0**
**a_99_NE2__a_202_O3**	**0**	**0**	**0**	**0**	**1**	**0**	**0**	**1**
**a_99_NE2__a_202_O7**	**0**	**0**	**0**	**0**	**0**	**0**	**0**	**0**
a_99_NE2__a_90_O	0	0	0	0	0	16	0	2
a_99_NE2__a_97_OH	0	0	0	1	0	2	0	3
a_99_NE2__a_98_O	0	0	0	0	0	0	0	0
a_99_NE2__a_99_O	0	0	0	0	0	0	0	0
a_99_N__a_92_O	236	221	214	218	108	142	107	126
a_99_N__a_98_ND1	0	0	0	0	0	0	0	0
a_99_N__a_99_OE1	0	0	0	0	0	0	1	0
a_9_N__a_10_O	0	0	0	0	0	0	0	0
a_9_N__a_11_OE1	0	0	0	0	0	0	0	0
a_9_N__a_11_OE2	0	0	0	0	0	0	0	0
a_9_N__a_122_O	242	248	232	221	247	240	220	223
a_9_N__a_123_OG	0	0	0	0	0	0	0	0
a_9_N__a_7_O	0	0	1	0	0	0	0	0
a_9_N__a_8_SD	1	1	2	2	1	1	2	2
a_9_N__a_9_O	0	0	0	0	1	5	15	8
a_9_N__a_9_OE2	0	0	0	1	0	0	0	0

Table S1 B

	3.9							
	Apo 283	*Apo 291*	*Apo 298*	*Apo 313*	*Holo 283*	*Holo 291*	*Holo 298*	*Holo 313*
a_100_N__a_110_OE1	0	0	0	0	0	0	3	0
a_100_N__a_111_O	248	244	242	242	245	242	230	241
a_100_N__a_99_OE1	4	2	8	8	1	0	0	0
a_100_OG1__a_100_O	0	0	3	0	0	0	2	3
a_100_OG1__a_101_O	0	0	0	0	0	3	0	7
a_100_OG1__a_111_O	21	40	29	39	13	33	11	30
a_100_OG1__a_90_O	7	10	16	6	32	9	26	17
a_100_OG1__a_98_ND1	0	0	0	0	0	0	0	0
a_100_OG1__a_98_NE2	0	1	0	0	2	0	2	0
a_100_OG1__a_99_O	14	21	21	11	5	3	7	2
a_101_N__a_100_OG1	0	0	1	0	0	0	0	0
a_101_N__a_90_O	245	234	244	239	112	90	122	116
a_101_N__a_99_OE1	0	0	2	0	0	0	0	0
a_101_OG__a_100_O	0	0	2	1	0	0	0	0
a_101_OG__a_101_O	0	0	0	0	0	0	0	0
a_101_OG__a_109_O	51	109	70	40	31	1	0	5
a_101_OG__a_110_OE1	1	2	3	3	9	9	14	10
a_101_OG__a_110_OE2	4	2	3	3	73	24	107	18
**a_101_OG__a_201_O**	**0**	**0**	**0**	**0**	**17**	**67**	**0**	**18**
**a_101_OG__a_201_O1**	**0**	**0**	**0**	**0**	**33**	**8**	**33**	**38**
**a_101_OG__a_201_O3**	**0**	**0**	**0**	**0**	**0**	**0**	**0**	**1**
a_101_OG__a_90_O	16	11	10	28	27	64	22	63
a_101_OG__a_99_OE1	57	52	48	47	1	2	1	8
a_102_N__a_100_O	0	0	0	0	0	0	0	1
a_102_N__a_101_OG	0	0	0	0	0	0	0	0
a_102_N__a_109_O	241	241	237	238	235	232	188	228
**a_102_N__a_201_O**	**0**	**0**	**0**	**0**	**0**	**1**	**0**	**0**
**a_102_N__a_201_O1**	**0**	**0**	**0**	**0**	**0**	**0**	**0**	**1**
a_103_N__a_102_OE1	3	0	1	1	58	51	42	50
a_103_N__a_102_OE2	3	0	1	2	59	69	45	64
a_103_N__a_88_O	41	165	117	140	0	1	0	1
a_104_N__a_102_O	1	0	0	1	0	0	0	0
a_104_N__a_107_O	237	237	234	240	242	240	241	235
a_105_N__a_103_O	0	0	0	0	0	0	0	0
a_105_N__a_107_O	9	44	45	34	0	1	2	10
a_106_N__a_104_O	0	1	0	1	0	0	1	2
a_106_N__a_106_OD1	0	0	0	0	0	0	1	0
a_106_N__a_106_OD2	0	0	2	3	0	1	0	0
a_107_NZ__a_106_OD1	2	6	2	6	1	5	8	1
a_107_NZ__a_106_OD2	0	4	1	0	2	7	6	6
a_107_NZ__a_10_OG	0	0	0	0	0	0	2	0
a_107_NZ__a_120_OE2	0	0	0	0	0	0	0	1
a_107_NZ__a_9_OE1	10	7	5	15	10	33	28	26
a_107_NZ__a_9_OE2	4	8	1	6	18	29	25	27
a_107_N__a_104_O	0	0	0	3	8	0	1	1
a_107_N__a_105_O	0	0	0	0	0	0	0	0
a_107_N__a_106_OD1	0	0	0	0	0	1	0	0

Table S1 B

a_107_N__a_106_OD2	0	0	0	0	0	0	0	2
a_108_N__a_123_O	248	248	247	248	243	243	235	233
a_108_N__a_123_OG	0	0	0	0	0	0	0	1
a_109_N__a_102_O	247	245	245	245	234	239	238	232
a_10_N__a_11_OE1	0	0	0	0	1	6	11	10
a_10_N__a_11_OE2	0	0	0	0	3	11	24	16
a_10_N__a_122_O	154	179	120	101	1	126	0	85
a_10_N__a_8_SD	0	0	0	1	2	0	0	0
a_10_N__a_9_OE1	0	0	1	0	0	1	14	0
a_10_N__a_9_OE2	0	0	0	0	0	1	13	0
a_10_OG__a_10_O	0	0	0	0	0	1	0	0
a_10_OG__a_11_O	0	0	0	1	0	0	1	0
a_10_OG__a_11_OE1	0	0	0	0	2	0	0	1
a_10_OG__a_11_OE2	0	0	0	0	1	0	0	1
a_10_OG__a_120_O	0	0	0	0	0	0	0	1
a_10_OG__a_122_O	0	0	1	1	0	0	0	0
a_10_OG__a_9_O	0	1	2	0	63	1	9	13
a_10_OG__a_9_OE1	116	100	75	81	2	52	60	53
a_10_OG__a_9_OE2	104	84	101	80	1	58	65	43
a_110_N__a_121_O	247	242	241	245	245	243	185	243
a_111_N__a_100_O	244	247	240	244	237	242	232	235
a_111_N__a_110_OE2	0	0	0	0	0	0	1	0
a_111_N__a_99_OE1	0	0	0	0	0	0	0	0
a_112_N__a_119_O	248	240	242	231	237	244	239	226
a_112_OG__a_110_O	1	0	0	0	0	1	1	3
a_112_OG__a_110_OE1	1	5	1	0	1	1	10	4
a_112_OG__a_110_OE2	0	6	0	0	3	0	0	5
a_112_OG__a_111_O	0	2	1	0	0	0	0	1
a_112_OG__a_112_O	0	0	0	0	0	0	0	0
a_112_OG__a_119_O	6	2	15	7	20	19	65	14
a_112_OG__a_119_OH	0	0	0	3	0	0	0	0
**a_112_OG__a_201_O2**	**0**	**0**	**0**	**0**	**0**	**1**	**0**	**0**
a_112_OG__a_97_OH	0	0	0	0	14	7	13	6
a_112_OG__a_98_O	2	21	7	16	1	38	9	10
a_112_OG__a_99_OE1	41	31	31	33	9	31	10	10
a_113_N__a_112_OG	0	0	0	0	0	0	0	0
a_113_N__a_98_O	248	242	243	244	244	225	237	240
a_113_OG1__a_111_O	0	0	0	0	0	0	0	0
a_113_OG1__a_112_O	0	0	0	1	3	1	0	2
a_113_OG1__a_113_O	0	0	0	0	1	0	1	0
a_113_OG1__a_114_O	0	1	2	0	0	0	0	0
a_113_OG1__a_115_O	0	0	0	0	0	0	0	1
a_113_OG1__a_117_O	0	2	10	13	0	11	0	15
a_113_OG1__a_118_OG1	24	32	84	32	79	50	56	14
a_113_OG1__a_98_ND1	1	0	0	3	0	0	0	0
a_113_OG1__a_98_O	14	9	6	17	14	8	23	29
a_114_N__a_113_OG1	0	2	1	0	0	0	0	0
a_114_N__a_117_O	196	153	173	200	234	239	230	228
a_114_N__a_118_OG1	1	0	0	0	0	0	0	0

Table S1 B

a_115_N__a_113_O	0	0	0	0	0	0	0	1
a_115_N__a_113_OG1	0	0	0	19	0	0	0	0
a_115_N__a_117_O	0	0	0	2	2	0	0	0
a_115_N__a_96_O	0	0	0	0	46	12	40	25
a_115_N__a_96_OD1	0	0	0	0	6	4	8	14
a_116_N__a_113_OG1	0	0	0	3	0	3	0	3
a_116_N__a_114_O	42	23	22	1	7	1	4	10
a_116_N__a_117_O	0	0	0	0	0	0	0	0
a_117_N__a_114_O	26	1	3	19	169	66	123	127
a_117_N__a_115_O	0	1	0	0	0	0	0	0
a_118_N__a_116_O	0	0	0	0	0	0	0	0
a_118_OG1__a_112_O	10	2	1	17	14	0	0	3
a_118_OG1__a_113_OG1	1	14	12	11	15	5	20	7
a_118_OG1__a_114_O	0	0	0	0	0	0	0	0
a_118_OG1__a_116_O	0	0	0	25	30	56	52	11
a_118_OG1__a_117_O	13	6	14	2	4	0	1	1
a_118_OG1__a_118_O	0	0	0	0	0	0	0	0
a_118_OG1__a_119_O	0	0	0	0	0	0	0	0
a_118_OG1__a_13_OD1	0	0	0	0	0	0	0	0
a_119_N__a_112_O	246	246	242	226	242	247	241	217
a_119_N__a_112_OG	0	0	0	0	0	0	0	6
a_119_N__a_118_OG1	0	0	0	0	0	0	0	0
a_119_OH__a_110_OE1	0	1	1	14	0	0	0	7
a_119_OH__a_110_OE2	0	0	0	1	0	0	0	3
a_119_OH__a_112_OG	0	0	0	0	0	1	0	1
a_119_OH__a_11_OE1	22	14	52	54	35	14	0	35
a_119_OH__a_11_OE2	95	102	111	104	6	35	0	4
a_119_OH__a_12_O	0	0	0	10	0	12	25	3
a_119_OH__a_13_O	0	0	0	0	0	5	0	0
a_119_OH__a_14_OH	0	0	0	0	1	0	0	0
**a_119_OH__a_201_N**	**0**	**0**	**0**	**0**	**0**	**0**	**0**	**0**
**a_119_OH__a_201_O2**	**0**	**0**	**0**	**0**	**0**	**6**	**0**	**20**
**a_119_OH__a_201_O4**	**0**	**0**	**0**	**0**	**9**	**7**	**73**	**23**
**a_119_OH__a_201_O5**	**0**	**0**	**0**	**0**	**22**	**10**	**67**	**15**
a_119_OH__a_74_SD	0	1	0	0	0	0	0	0
a_119_OH__a_8_SD	0	0	0	0	0	0	1	0
a_119_OH__a_97_OH	0	0	0	1	0	0	0	3
a_119_OH__a_99_OE1	0	0	0	0	0	0	0	2
a_11_N__a_10_OG	0	0	0	0	0	0	0	0
a_11_N__a_11_OE1	18	15	6	1	12	28	106	36
a_11_N__a_11_OE2	7	11	6	3	17	33	78	66
a_120_N__a_120_OE1	0	0	0	1	0	0	3	0
a_120_N__a_120_OE2	0	0	0	1	0	0	0	0
a_120_N__a_12_O	29	20	0	52	196	165	129	143
a_120_N__a_13_OD1	0	0	0	1	0	0	0	4
a_121_NE__a_110_OE1	0	1	32	30	0	0	0	0
a_121_NE__a_110_OE2	0	1	40	49	0	0	0	16
a_121_NE__a_112_OG	0	0	0	0	0	1	1	0
a_121_NE__a_119_O	0	0	0	0	0	1	0	0

a_121_NE__a_119_OH	24	79	48	33	88	85	11	54
a_121_NE__a_11_OE1	11	0	1	12	0	0	0	1
a_121_NE__a_11_OE2	20	15	2	0	0	9	0	5
a_121_NE__a_123_OG	1	1	0	0	0	0	0	0
a_121_NE__a_14_OH	0	0	0	1	0	0	0	0
a_121_NE__a_16_OE1	0	0	0	0	0	0	0	4
a_121_NE__a_16_OE2	0	0	0	0	0	0	0	2
**a_121_NE__a_201_N**	**0**	**0**	**0**	**0**	**4**	**0**	**10**	**0**
**a_121_NE__a_201_O1**	**0**	**0**	**0**	**0**	**0**	**0**	**0**	**3**
**a_121_NE__a_201_O2**	**0**	**0**	**0**	**0**	**2**	**7**	**7**	**20**
**a_121_NE__a_201_O4**	**0**	**0**	**0**	**0**	**2**	**4**	**54**	**6**
**a_121_NE__a_201_O5**	**0**	**0**	**0**	**0**	**16**	**3**	**24**	**6**
a_121_NE__a_74_SD	0	0	3	0	0	0	0	0
a_121_NE__a_8_SD	16	9	2	5	11	6	0	7
a_121_NH1__a_110_OE1	39	63	72	55	156	148	134	131
a_121_NH1__a_110_OE2	123	136	82	65	52	57	35	50
a_121_NH1__a_112_OG	0	0	0	0	5	0	18	2
a_121_NH1__a_119_OH	1	13	5	7	0	0	0	0
a_121_NH1__a_11_OE1	0	0	0	7	0	0	0	0
a_121_NH1__a_11_OE2	0	0	0	6	0	0	0	0
a_121_NH1__a_121_O	0	0	0	1	0	0	0	0
a_121_NH1__a_123_OG	11	8	12	8	0	0	0	0
a_121_NH1__a_14_OH	0	0	9	0	0	0	0	0
**a_121_NH1__a_201_N**	**0**	**0**	**0**	**0**	**1**	**0**	**0**	**7**
**a_121_NH1__a_201_O1**	**0**	**0**	**0**	**0**	**2**	**0**	**4**	**0**
**a_121_NH1__a_201_O2**	**0**	**0**	**0**	**0**	**8**	**14**	**0**	**26**
**a_121_NH1__a_201_O3**	**0**	**0**	**0**	**0**	**14**	**57**	**37**	**20**
**a_121_NH1__a_201_O4**	**0**	**0**	**0**	**0**	**1**	**0**	**8**	**9**
**a_121_NH1__a_201_O5**	**0**	**0**	**0**	**0**	**0**	**1**	**0**	**9**
**a_121_NH1__a_202_O3**	**0**	**0**	**0**	**0**	**2**	**28**	**39**	**15**
**a_121_NH1__a_202_O7**	**0**	**0**	**0**	**0**	**7**	**12**	**33**	**10**
a_121_NH1__a_51_OE1	1	9	6	1	0	0	0	0
a_121_NH1__a_53_OH	0	0	1	6	0	0	0	0
a_121_NH1__a_73_O	0	0	1	0	0	0	0	0
a_121_NH1__a_74_SD	0	2	7	3	0	0	0	0
a_121_NH1__a_8_SD	0	0	1	0	0	0	0	0
a_121_NH1__a_97_OH	0	0	0	0	0	0	2	2
a_121_NH1__a_99_OE1	0	0	0	0	2	7	2	1
a_121_NH2__a_110_OE1	7	40	58	68	2	0	0	11
a_121_NH2__a_110_OE2	3	5	60	50	0	0	0	8
a_121_NH2__a_112_OG	0	0	0	0	0	5	3	1
a_121_NH2__a_119_O	0	0	0	0	0	1	0	0
a_121_NH2__a_119_OH	6	21	17	10	8	26	8	7
a_121_NH2__a_11_OE1	3	0	0	3	0	0	0	0
a_121_NH2__a_11_OE2	11	3	0	0	0	0	0	3
a_121_NH2__a_13_OD1	0	0	0	0	0	0	0	1
a_121_NH2__a_14_OH	15	5	23	5	0	0	0	1
a_121_NH2__a_16_OE2	0	0	0	0	0	0	0	2
**a_121_NH2__a_201_N**	**0**	**0**	**0**	**0**	**3**	**8**	**5**	**14**

Table S1 B

**a_121_NH2__a_201_O2**	**0**	**0**	**0**	**0**	**18**	**50**	**55**	**39**
**a_121_NH2__a_201_O3**	**0**	**0**	**0**	**0**	**2**	**0**	**9**	**9**
**a_121_NH2__a_201_O4**	**0**	**0**	**0**	**0**	**38**	**26**	**37**	**25**
**a_121_NH2__a_201_O5**	**0**	**0**	**0**	**0**	**56**	**36**	**27**	**30**
**a_121_NH2__a_202_O7**	**0**	**0**	**0**	**0**	**1**	**4**	**57**	**11**
a_121_NH2__a_31_O	0	0	0	1	0	0	0	0
a_121_NH2__a_51_OE1	8	5	9	1	0	0	0	0
a_121_NH2__a_53_OH	0	0	1	3	0	0	0	0
a_121_NH2__a_74_SD	0	9	14	5	0	0	1	0
a_121_NH2__a_8_SD	12	3	0	2	2	4	0	2
a_121_NH2__a_97_OH	0	0	0	0	3	0	0	6
a_121_NH2__a_99_OE1	0	0	0	2	0	0	0	0
a_121_N__a_110_O	245	247	246	246	247	247	247	247
a_121_N__a_119_O	0	0	0	0	0	0	0	0
a_122_N__a_10_O	186	216	136	111	224	163	10	115
a_122_N__a_10_OG	9	0	3	5	0	1	0	1
a_122_N__a_120_O	0	0	0	0	0	0	1	0
a_122_N__a_8_SD	0	0	0	0	1	1	0	7
a_122_N__a_9_O	0	0	0	0	0	4	2	2
a_123_N__a_108_O	246	248	244	244	247	247	242	237
**a_123_N__a_201_O**	**0**	**0**	**0**	**0**	**0**	**0**	**0**	**0**
a_123_OG__a_108_O	16	19	47	15	9	10	20	31
a_123_OG__a_121_O	0	0	0	0	0	0	2	0
a_123_OG__a_122_O	1	0	0	2	2	1	1	1
a_123_OG__a_123_O	0	0	0	0	0	0	0	0
a_123_OG__a_124_O	0	0	0	0	5	2	3	3
**a_123_OG__a_201_O**	**0**	**0**	**0**	**0**	**0**	**0**	**0**	**2**
**a_123_OG__a_201_O1**	**0**	**0**	**0**	**0**	**0**	**6**	**0**	**7**
a_123_OG__a_7_O	13	3	3	7	194	141	157	124
a_123_OG__a_8_SD	0	2	1	5	0	0	0	1
a_124_NZ__a_106_OD1	3	2	21	13	7	10	13	10
a_124_NZ__a_106_OD2	2	9	12	3	3	15	21	10
a_124_NZ__a_125_O	3	4	18	8	28	21	25	12
a_124_NZ__a_7_OE1	3	3	5	8	31	15	21	15
a_124_NZ__a_7_OE2	4	3	3	8	35	27	12	10
a_124_NZ__a_8_O	1	0	0	0	5	0	0	0
a_124_NZ__a_9_OE1	0	0	0	0	23	2	3	5
a_124_NZ__a_9_OE2	0	0	0	3	21	3	2	3
a_124_N__a_123_OG	0	0	0	0	0	1	0	0
a_124_N__a_7_O	248	240	248	246	236	232	245	208
a_125_NE__a_105_O	2	4	1	9	3	0	1	5
a_125_NE__a_106_O	0	0	0	0	23	1	13	11
a_125_NE__a_106_OD1	10	14	11	5	31	7	9	26
a_125_NE__a_106_OD2	18	6	11	11	43	17	14	26
a_125_NE__a_125_O	0	0	0	0	1	0	0	0
a_125_NE__a_126_O	5	5	10	2	1	0	4	2
a_125_NE__a_3_O	0	0	0	1	0	1	0	2
a_125_NE__a_5_O	0	0	0	0	0	0	1	0
a_125_NH1__a_105_O	9	10	34	26	4	3	12	12

Table S1 B

a_125_NH1__a_106_O	0	0	0	0	0	0	0	3
a_125_NH1__a_106_OD1	14	11	0	2	8	7	7	4
a_125_NH1__a_106_OD2	5	4	2	0	1	7	5	11
a_125_NH1__a_125_O	5	1	1	1	0	0	1	2
a_125_NH1__a_126_O	0	0	0	4	0	0	0	3
a_125_NH1__a_3_O	0	0	1	0	2	0	0	4
a_125_NH1__a_5_O	0	0	0	1	0	0	0	0
a_125_NH2__a_105_O	0	0	0	0	2	0	0	1
a_125_NH2__a_106_O	0	0	0	0	1	0	0	0
a_125_NH2__a_106_OD1	6	17	12	5	46	13	21	48
a_125_NH2__a_106_OD2	22	10	14	14	75	15	7	26
a_125_NH2__a_126_O	0	0	0	0	0	0	0	0
a_125_NH2__a_3_O	0	1	2	4	0	0	0	3
a_125_N__a_106_O	245	245	234	247	94	145	187	131
a_125_N__a_106_OD1	0	0	0	0	0	0	0	0
a_125_N__a_106_OD2	0	0	0	0	0	0	0	0
a_126_N__a_124_O	8	0	0	8	0	0	0	0
a_126_N__a_5_O	224	230	227	222	213	240	222	207
a_127_N__a_125_O	0	0	2	0	0	0	0	0
a_127_N__a_5_O	0	1	7	23	11	30	17	11
a_12_NZ__a_10_O	2	1	0	0	0	20	95	38
a_12_NZ__a_10_OG	41	30	27	29	19	37	40	30
a_12_NZ__a_118_O	0	0	0	12	0	0	0	0
a_12_NZ__a_118_OG1	0	0	0	0	0	0	0	0
a_12_NZ__a_11_O	1	0	0	1	0	0	1	0
a_12_NZ__a_120_O	4	5	14	9	0	0	0	2
a_12_NZ__a_120_OE1	67	54	51	58	58	49	63	54
a_12_NZ__a_120_OE2	50	70	74	44	56	72	46	60
a_12_NZ__a_13_OD1	0	0	0	4	1	1	0	1
a_12_NZ__a_16_OE1	0	0	0	2	0	0	0	2
a_12_NZ__a_16_OE2	0	0	0	2	0	0	0	0
a_12_NZ__a_9_OE1	1	0	1	1	0	0	0	1
a_12_NZ__a_9_OE2	0	0	1	0	0	0	0	0
a_12_N__a_10_O	0	0	0	0	0	0	0	0
a_12_N__a_119_OH	48	22	55	30	0	0	0	0
a_12_N__a_11_OE1	1	0	5	30	0	0	0	3
a_12_N__a_11_OE2	2	5	40	39	0	0	0	4
a_12_N__a_120_O	172	195	95	78	229	196	141	169
**a_12_N__a_201_O4**	**0**	**0**	**0**	**0**	**0**	**0**	**13**	**0**
**a_12_N__a_201_O5**	**0**	**0**	**0**	**0**	**0**	**0**	**6**	**0**
a_13_ND2__a_118_O	86	28	25	80	173	136	133	142
a_13_ND2__a_118_OG1	0	0	0	0	0	1	0	0
a_13_ND2__a_119_OH	0	0	0	0	0	0	1	1
a_13_ND2__a_120_O	0	0	0	0	0	0	0	0
a_13_ND2__a_12_O	0	0	1	0	0	0	0	2
a_13_ND2__a_13_O	7	1	11	11	19	16	25	15
a_13_ND2__a_15_OD1	1	2	16	14	0	0	0	0
a_13_ND2__a_15_OD2	1	3	5	15	0	0	0	0
a_13_ND2__a_16_O	1	0	0	0	0	0	0	0

a_13_ND2__a_16_OE1	14	17	19	21	1	7	0	12
a_13_ND2__a_16_OE2	10	10	16	11	3	9	8	7
a_13_ND2__a_8_SD	0	0	0	0	0	0	0	0
a_13_N__a_119_OH	3	0	8	50	0	0	0	0
a_13_N__a_11_O	0	0	0	0	0	0	0	2
a_13_N__a_11_OE1	0	0	5	7	1	4	0	1
a_13_N__a_11_OE2	0	0	12	7	0	8	0	0
a_13_N__a_13_OD1	0	0	1	0	0	0	0	0
a_14_N__a_119_OH	0	0	0	0	0	0	0	0
a_14_N__a_11_O	0	0	0	0	0	0	0	0
a_14_N__a_11_OE1	0	0	21	53	1	0	0	1
a_14_N__a_11_OE2	0	0	7	41	0	0	0	2
a_14_N__a_12_O	121	62	52	17	1	0	0	0
a_14_N__a_13_OD1	0	0	0	0	0	1	0	0
a_14_N__a_15_OD1	1	16	2	0	0	0	0	0
a_14_N__a_15_OD2	0	4	0	0	0	0	0	0
**a_14_N__a_201_O4**	**0**	**0**	**0**	**0**	**0**	**0**	**1**	**0**
a_14_OH__a_112_OG	0	0	1	1	0	0	0	0
a_14_OH__a_119_OH	0	0	0	1	0	0	0	0
a_14_OH__a_11_OE1	0	1	0	0	15	3	4	7
a_14_OH__a_11_OE2	0	0	1	0	0	4	9	3
a_14_OH__a_15_OD2	0	0	0	0	0	8	0	0
a_14_OH__a_18_SD	0	1	0	0	0	0	0	0
**a_14_OH__a_201_N**	**0**	**0**	**0**	**0**	**0**	**0**	**0**	**0**
**a_14_OH__a_201_O4**	**0**	**0**	**0**	**0**	**19**	**8**	**0**	**1**
**a_14_OH__a_201_O5**	**0**	**0**	**0**	**0**	**24**	**14**	**0**	**4**
a_14_OH__a_24_O	0	0	0	0	1	0	0	0
a_14_OH__a_27_O	0	2	2	0	0	0	0	0
a_14_OH__a_28_O	131	11	32	23	2	1	28	24
a_14_OH__a_29_OE1	0	0	0	4	2	2	0	1
a_14_OH__a_29_OE2	0	1	0	3	1	2	0	0
a_14_OH__a_53_OH	0	0	0	0	0	0	0	0
a_14_OH__a_73_OG1	0	0	1	0	0	0	0	0
a_14_OH__a_74_O	0	1	2	1	0	0	0	0
a_14_OH__a_74_SD	1	42	26	23	0	0	0	0
a_14_OH__a_97_OH	0	1	0	0	0	0	0	0
a_15_N__a_12_O	0	0	0	0	0	0	0	4
a_15_N__a_13_O	0	0	0	0	0	0	0	2
a_15_N__a_13_OD1	4	19	8	8	1	3	0	0
a_15_N__a_15_OD1	9	0	8	2	0	1	0	0
a_15_N__a_15_OD2	1	2	1	2	0	3	1	2
a_15_N__a_16_OE1	0	1	0	0	0	0	0	0
a_16_N__a_13_O	12	0	13	76	11	17	43	41
a_16_N__a_13_OD1	22	73	58	18	3	19	0	0
a_16_N__a_14_O	0	0	0	0	0	0	0	0
a_16_N__a_15_OD1	6	3	6	2	0	1	3	1
a_16_N__a_15_OD2	1	2	0	2	0	1	2	2
a_16_N__a_16_O	0	0	0	0	0	0	0	1
a_16_N__a_16_OE1	0	10	1	1	0	7	6	0

Table S1 B

a_16_N__a_16_OE2	0	0	2	1	1	1	0	1
a_17_N__a_13_O	16	1	3	40	32	46	74	78
a_17_N__a_13_OD1	0	0	1	0	0	0	0	0
a_17_N__a_14_O	164	114	164	161	92	88	81	76
a_17_N__a_15_O	0	0	0	0	0	0	0	0
a_18_N__a_14_O	246	231	229	236	214	203	223	177
a_18_N__a_15_O	4	5	15	8	0	14	11	3
a_18_N__a_16_O	1	1	0	0	3	7	2	6
a_18_N__a_18_SD	0	0	0	0	0	0	0	0
a_19_NZ__a_15_O	10	5	0	0	7	1	8	6
a_19_NZ__a_15_OD1	1	4	4	7	4	9	17	22
a_19_NZ__a_15_OD2	1	16	7	2	3	12	31	24
a_19_NZ__a_16_O	0	0	1	0	0	1	0	2
a_19_NZ__a_16_OE1	87	78	76	62	96	80	73	57
a_19_NZ__a_16_OE2	78	81	91	82	95	98	84	76
a_19_NZ__a_24_O	0	0	0	0	0	0	3	1
a_19_N__a_15_O	190	195	204	203	83	122	139	123
a_19_N__a_16_O	27	107	69	30	129	141	125	111
a_19_N__a_17_O	114	44	48	68	72	58	59	42
a_19_N__a_18_SD	0	0	0	0	0	0	0	0
a_19_N__a_23_O	0	0	0	0	0	0	0	0
**a_201_O__a_101_O**	**0**	**0**	**0**	**0**	**6**	**0**	**0**	**4**
**a_201_O__a_101_OG**	**0**	**0**	**0**	**0**	**28**	**25**	**48**	**21**
**a_201_O__a_102_O**	**0**	**0**	**0**	**0**	**0**	**1**	**0**	**0**
**a_201_O__a_108_O**	**0**	**0**	**0**	**0**	**0**	**0**	**0**	**1**
**a_201_O__a_109_O**	**0**	**0**	**0**	**0**	**0**	**1**	**0**	**2**
**a_201_O__a_110_OE1**	**0**	**0**	**0**	**0**	**18**	**17**	**0**	**36**
**a_201_O__a_110_OE2**	**0**	**0**	**0**	**0**	**22**	**101**	**24**	**30**
**a_201_O__a_201_O1**	**0**	**0**	**0**	**0**	**0**	**2**	**7**	**0**
**a_201_O__a_202_O3**	**0**	**0**	**0**	**0**	**42**	**15**	**21**	**23**
**a_201_O__a_2_O**	**0**	**0**	**0**	**0**	**1**	**0**	**9**	**0**
**a_201_O__a_85_SD**	**0**	**0**	**0**	**0**	**1**	**0**	**0**	**0**
**a_201_O__a_88_O**	**0**	**0**	**0**	**0**	**10**	**5**	**1**	**26**
**a_201_O__a_90_O**	**0**	**0**	**0**	**0**	**1**	**0**	**0**	**0**
**a_201_O__a_99_OE1**	**0**	**0**	**0**	**0**	**13**	**0**	**2**	**0**
a_20_N__a_16_O	14	109	52	17	45	64	45	66
a_20_N__a_17_O	126	115	138	158	185	164	175	173
a_20_N__a_18_O	0	0	0	0	0	0	0	0
a_21_N__a_17_O	22	77	45	41	22	40	20	42
a_21_N__a_18_O	44	78	104	101	148	142	157	126
a_21_N__a_19_O	0	0	0	0	0	0	0	0
a_22_N__a_18_O	39	170	129	116	147	150	165	184
a_22_N__a_19_O	7	63	74	55	59	54	58	36
a_22_N__a_20_O	0	0	0	0	0	0	0	0
a_23_N__a_18_O	43	187	132	118	134	112	203	157
a_23_N__a_19_O	0	0	2	0	0	0	0	0
**a_23_N__a_202_OT2**	**0**	**0**	**0**	**0**	**0**	**1**	**0**	**0**
a_23_N__a_21_O	0	0	0	0	0	0	0	0
a_24_N__a_22_O	0	0	0	0	2	1	1	1

a_24_N__a_74_SD	0	0	0	0	0	0	0	2
a_24_OG__a_15_O	0	0	0	0	0	0	1	0
a_24_OG__a_22_O	0	0	0	0	11	16	3	9
a_24_OG__a_23_O	0	0	2	0	1	0	0	5
a_24_OG__a_24_O	0	0	0	0	0	0	0	0
a_24_OG__a_26_OD1	15	15	33	11	0	0	0	1
a_24_OG__a_26_OD2	5	10	10	12	0	0	0	0
a_24_OG__a_75_O	0	4	0	0	0	0	0	0
a_25_N__a_15_OD2	0	0	0	0	0	0	1	0
**a_25_N__a_201_O4**	**0**	**0**	**0**	**0**	**0**	**0**	**0**	**1**
a_25_N__a_23_O	0	2	0	0	0	0	0	0
a_25_N__a_24_OG	0	0	0	0	0	0	0	0
a_25_N__a_74_SD	0	0	0	0	0	0	0	1
a_25_OG__a_14_OH	0	0	0	2	0	0	0	2
a_25_OG__a_15_OD1	0	0	4	7	0	0	0	0
a_25_OG__a_15_OD2	0	1	4	4	0	0	2	0
a_25_OG__a_18_SD	0	0	0	0	0	1	1	0
**a_25_OG__a_201_O4**	**0**	**0**	**0**	**0**	**0**	**0**	**0**	**5**
**a_25_OG__a_201_O5**	**0**	**0**	**0**	**0**	**0**	**0**	**0**	**3**
a_25_OG__a_23_O	0	2	0	0	0	2	0	0
a_25_OG__a_24_O	0	0	0	0	1	23	0	2
a_25_OG__a_24_OG	0	0	0	0	3	4	7	1
a_25_OG__a_25_O	0	0	0	0	0	0	0	1
a_25_OG__a_26_OD1	0	0	0	0	0	0	0	2
a_25_OG__a_29_OE1	0	0	9	1	3	7	1	11
a_25_OG__a_29_OE2	0	0	4	0	3	7	4	5
a_25_OG__a_74_O	0	0	0	0	0	0	0	1
a_25_OG__a_74_SD	0	0	0	0	0	0	0	2
a_26_N__a_24_O	0	0	9	8	0	0	2	0
a_26_N__a_24_OG	8	19	6	14	1	2	5	1
a_26_N__a_25_OG	0	0	0	0	0	0	0	1
a_26_N__a_26_OD1	1	0	1	0	0	0	0	0
a_26_N__a_26_OD2	0	0	0	1	0	0	0	0
a_26_N__a_29_OE1	0	0	7	0	0	0	0	1
a_26_N__a_29_OE2	0	0	14	0	0	0	0	2
a_27_N__a_24_O	187	130	123	155	0	0	7	11
a_27_N__a_24_OG	0	0	0	1	0	0	0	0
a_27_N__a_25_O	0	0	7	0	0	0	10	29
a_27_N__a_25_OG	0	0	0	0	0	1	0	9
a_27_N__a_26_OD1	0	0	1	0	0	4	0	1
a_27_N__a_26_OD2	0	0	1	0	1	0	0	1
a_28_N__a_24_O	50	19	35	41	0	0	0	3
a_28_N__a_25_O	29	96	70	69	138	102	51	53
a_28_N__a_25_OG	0	0	0	0	0	0	0	3
a_28_N__a_26_O	0	0	0	0	0	0	0	0
a_28_N__a_29_OE2	0	0	0	0	0	0	0	0
a_29_N__a_25_O	185	195	175	197	205	219	173	66
a_29_N__a_26_O	15	13	15	16	11	20	40	119
a_29_N__a_27_O	0	0	0	0	0	0	0	0

Table S1 B

a_29_N__a_29_OE1	0	0	4	0	3	0	0	0
a_29_N__a_29_OE2	0	0	14	0	5	0	0	0
a_2_N__a_41_OE1	0	0	0	0	0	0	1	0
a_2_N__a_42_O	0	0	0	1	35	45	34	34
a_2_N__a_42_OE1	0	0	0	24	115	175	127	122
a_2_N__a_85_SD	0	0	0	1	0	0	0	0
a_2_N__a_87_O	0	0	0	0	0	0	0	0
a_2_N__a_88_O	0	0	0	0	2	0	2	8
**a_30_NZ__a_201_O4**	**0**	**0**	**0**	**0**	**0**	**0**	**0**	**0**
**a_30_NZ__a_201_O5**	**0**	**0**	**0**	**0**	**4**	**0**	**0**	**0**
a_30_NZ__a_25_O	0	0	0	1	1	0	0	0
a_30_NZ__a_26_O	29	34	27	36	32	33	31	13
a_30_NZ__a_26_OD1	85	70	90	63	49	62	69	93
a_30_NZ__a_26_OD2	93	108	91	69	70	62	37	80
a_30_NZ__a_27_O	0	0	0	1	0	0	0	0
a_30_NZ__a_29_OE1	0	0	0	1	9	13	8	9
a_30_NZ__a_29_OE2	0	0	0	1	1	2	0	0
a_30_NZ__a_33_OD1	1	0	0	1	0	0	0	0
a_30_NZ__a_53_OH	0	0	0	0	12	0	2	20
a_30_NZ__a_54_O	0	0	0	0	0	0	2	2
a_30_NZ__a_54_OG	0	0	0	0	0	0	3	0
a_30_NZ__a_56_O	0	0	0	1	0	0	0	0
a_30_NZ__a_57_ND1	0	1	0	0	0	0	0	0
a_30_NZ__a_75_O	0	0	3	0	0	0	0	0
a_30_N__a_26_O	120	94	124	68	69	64	34	158
a_30_N__a_27_O	34	26	43	62	43	75	93	47
a_30_N__a_28_O	0	0	0	0	0	0	0	0
a_31_N__a_27_O	182	131	129	110	191	212	199	175
a_31_N__a_28_O	33	103	83	105	23	19	18	14
a_31_N__a_29_O	0	0	0	0	0	0	0	0
a_32_NE__a_11_OE1	31	42	86	66	16	22	7	12
a_32_NE__a_11_OE2	23	40	43	54	35	11	18	3
a_32_NE__a_12_O	0	0	0	1	0	0	0	0
a_32_NE__a_13_OD1	0	0	0	1	0	0	0	0
a_32_NE__a_14_OH	1	0	0	2	3	3	14	7
a_32_NE__a_15_OD1	4	0	0	0	0	0	0	0
a_32_NE__a_15_OD2	2	0	0	8	0	0	0	0
a_32_NE__a_28_O	0	0	0	0	1	0	0	7
a_32_NE__a_29_OE1	0	3	0	5	26	22	32	30
a_32_NE__a_29_OE2	0	4	0	18	28	42	29	33
a_32_NE__a_33_OD1	0	0	0	0	0	0	3	0
a_32_NH1__a_11_O	0	1	0	0	0	0	0	0
a_32_NH1__a_11_OE1	0	11	1	0	34	40	6	10
a_32_NH1__a_11_OE2	0	4	2	0	32	12	8	29
a_32_NH1__a_12_O	0	4	2	0	0	0	0	0
a_32_NH1__a_13_OD1	0	0	1	4	0	0	0	0
a_32_NH1__a_14_OH	0	0	0	0	10	3	21	20
a_32_NH1__a_15_OD1	20	52	9	14	18	36	12	24
a_32_NH1__a_15_OD2	26	49	42	41	29	4	12	23

Table S1 B

a_32_NH1__a_16_OE2	0	0	0	1	0	0	0	0
a_32_NH1__a_18_SD	0	0	0	0	0	2	0	0
**a_32_NH1__a_201_O4**	**0**	**0**	**0**	**0**	**1**	**0**	**0**	**0**
**a_32_NH1__a_201_O5**	**0**	**0**	**0**	**0**	**5**	**0**	**0**	**0**
a_32_NH1__a_28_O	0	0	0	1	3	0	1	1
a_32_NH1__a_29_OE1	12	22	9	22	6	6	10	14
a_32_NH1__a_29_OE2	27	14	12	25	5	6	33	27
a_32_NH1__a_32_O	0	0	1	0	0	0	0	0
a_32_NH1__a_33_OD1	0	0	0	0	2	1	5	0
a_32_NH2__a_119_OH	0	0	3	0	0	0	0	0
a_32_NH2__a_11_O	1	0	0	1	2	0	11	6
a_32_NH2__a_11_OE1	64	56	110	79	40	19	9	7
a_32_NH2__a_11_OE2	42	32	42	38	30	33	7	11
a_32_NH2__a_12_O	3	22	55	25	0	0	0	0
a_32_NH2__a_13_OD1	0	0	0	10	0	0	0	0
a_32_NH2__a_14_O	0	0	0	0	0	0	0	0
a_32_NH2__a_14_OH	0	0	0	1	2	2	1	2
a_32_NH2__a_15_OD1	26	52	64	42	26	5	19	13
a_32_NH2__a_15_OD2	25	53	11	22	2	26	2	28
a_32_NH2__a_18_SD	0	0	0	0	0	1	0	0
a_32_NH2__a_25_OG	0	0	0	0	0	2	0	0
a_32_NH2__a_28_O	0	0	0	0	0	0	0	9
a_32_NH2__a_29_OE1	8	4	5	25	53	55	88	47
a_32_NH2__a_29_OE2	2	8	3	10	51	43	53	36
a_32_NH2__a_33_OD1	0	0	0	0	0	0	0	1
a_32_N__a_14_OH	0	0	0	0	1	0	0	0
a_32_N__a_28_O	50	108	85	88	166	187	118	60
a_32_N__a_29_O	130	78	109	68	25	25	50	108
a_32_N__a_30_O	0	0	0	0	0	0	0	0
a_33_ND2__a_11_OE1	0	0	0	1	0	0	0	0
a_33_ND2__a_29_O	3	3	2	13	47	50	29	28
a_33_ND2__a_29_OE1	1	0	1	2	10	6	8	5
a_33_ND2__a_29_OE2	0	0	1	1	9	9	5	4
a_33_ND2__a_30_O	2	8	0	1	0	2	0	0
a_33_ND2__a_32_O	0	0	0	0	0	0	0	0
a_33_ND2__a_33_O	7	5	1	3	1	2	1	1
a_33_ND2__a_53_O	0	10	1	0	0	0	2	0
a_33_ND2__a_53_OH	0	0	0	0	0	0	0	0
a_33_ND2__a_54_O	0	10	2	13	0	0	4	0
a_33_ND2__a_54_OG	4	6	4	10	0	1	6	24
a_33_N__a_11_OE1	0	0	0	1	0	0	0	0
a_33_N__a_29_O	24	11	32	36	160	92	142	82
a_33_N__a_30_O	144	80	66	54	54	68	40	108
a_33_N__a_31_O	0	0	0	3	0	0	1	0
a_33_N__a_33_OD1	0	0	0	0	0	0	1	0
a_33_N__a_34_O	0	0	0	0	0	0	0	0
a_33_N__a_53_O	0	0	0	0	0	0	0	0
a_33_N__a_54_OG	0	0	0	5	0	0	0	0
a_34_N__a_29_O	0	0	0	0	6	0	0	0

Table S1 B

a_34_N__a_30_O	57	26	40	62	197	168	192	189
a_34_N__a_31_O	16	21	14	16	8	39	16	6
a_34_N__a_32_O	0	0	0	2	0	0	0	0
a_34_N__a_33_OD1	0	1	0	3	0	0	3	0
a_34_N__a_53_O	0	3	9	0	0	0	0	0
a_34_N__a_54_OG	0	0	0	7	0	0	0	0
a_35_NZ__a_11_OE1	0	0	0	0	2	7	47	20
a_35_NZ__a_11_OE2	0	0	0	0	20	22	59	25
**a_35_NZ__a_201_O4**	**0**	**0**	**0**	**0**	**1**	**0**	**0**	**0**
**a_35_NZ__a_201_O5**	**0**	**0**	**0**	**0**	**2**	**0**	**0**	**0**
a_35_NZ__a_31_O	0	0	0	0	4	0	11	2
a_35_NZ__a_32_O	0	0	0	0	9	0	17	1
a_35_NZ__a_33_O	0	0	0	0	1	0	0	0
a_35_NZ__a_36_O	3	0	0	0	0	2	0	0
a_35_NZ__a_54_OG	1	0	0	0	0	0	0	0
a_35_NZ__a_7_OE1	64	34	66	55	73	60	24	31
a_35_NZ__a_7_OE2	67	52	75	97	75	60	16	34
a_35_NZ__a_8_O	22	32	22	14	57	64	43	82
a_35_NZ__a_8_SD	0	0	0	0	2	0	0	0
a_35_NZ__a_9_O	14	51	21	12	0	6	4	29
a_35_NZ__a_9_OE1	0	2	0	0	41	3	12	3
a_35_NZ__a_9_OE2	8	10	10	13	39	2	7	8
a_35_N__a_30_O	0	0	0	0	114	58	125	91
a_35_N__a_31_O	0	0	0	0	97	122	67	28
a_35_N__a_32_O	0	0	0	0	2	12	4	0
a_35_N__a_33_O	0	0	0	2	0	0	0	0
a_35_N__a_33_OD1	0	0	0	7	0	0	0	0
a_35_N__a_53_O	0	0	0	8	0	0	0	0
a_35_N__a_54_OG	0	0	0	13	0	0	0	0
a_35_N__a_8_SD	0	0	0	0	0	0	0	0
**a_36_N__a_201_O4**	**0**	**0**	**0**	**0**	**1**	**2**	**0**	**2**
**a_36_N__a_201_O5**	**0**	**0**	**0**	**0**	**0**	**2**	**0**	**0**
a_36_N__a_31_O	0	0	0	0	2	0	0	0
a_36_N__a_34_O	0	8	10	3	0	0	0	0
a_36_N__a_8_SD	59	71	65	88	20	23	50	38
a_37_N__a_35_O	0	0	1	0	0	0	0	0
a_37_N__a_51_OE1	0	0	0	0	0	0	0	0
a_37_N__a_52_O	237	223	196	190	204	199	231	192
a_38_N__a_6_O	247	246	247	245	184	211	234	223
a_38_OG1__a_123_OG	0	0	1	0	0	0	0	0
**a_38_OG1__a_201_O4**	**0**	**0**	**0**	**0**	**4**	**0**	**0**	**4**
a_38_OG1__a_36_O	0	0	2	0	0	0	0	0
a_38_OG1__a_37_O	0	0	0	4	5	3	17	9
a_38_OG1__a_38_O	0	0	0	0	0	0	0	0
a_38_OG1__a_39_O	0	0	0	0	0	5	0	1
a_38_OG1__a_50_O	0	27	38	35	133	156	176	107
a_38_OG1__a_51_OE1	138	23	60	10	12	7	2	2
a_38_OG1__a_6_O	3	18	21	21	7	25	2	24
a_38_OG1__a_8_SD	0	0	1	0	0	0	0	0

Table S1 B

a_39_N__a_37_O	0	0	0	0	0	0	0	0
a_39_N__a_38_OG1	0	0	0	0	0	0	0	1
a_39_N__a_39_OE1	0	0	0	0	0	0	0	8
a_39_N__a_39_OE2	0	0	0	0	0	0	0	0
a_39_N__a_50_O	248	237	234	242	226	223	202	224
a_39_N__a_50_OG	0	2	0	1	2	4	3	1
a_3_N__a_1_O	11	0	0	13	13	27	13	21
a_3_OG1__a_1_O	0	0	0	3	1	14	0	11
a_3_OG1__a_2_O	9	2	0	3	3	0	0	0
a_3_OG1__a_3_O	0	0	0	1	1	0	0	0
a_3_OG1__a_40_O	20	8	41	17	0	4	19	3
a_3_OG1__a_41_OE1	10	18	11	4	15	13	8	1
a_3_OG1__a_42_O	0	0	0	0	40	19	6	10
a_3_OG1__a_42_OE1	1	1	4	0	0	0	1	1
a_40_N__a_4_O	247	244	239	237	246	235	238	244
a_41_NE2__a_39_OE1	2	2	2	14	1	3	5	2
a_41_NE2__a_39_OE2	5	5	2	9	2	1	5	3
a_41_NE2__a_3_OG1	0	0	0	0	5	0	0	0
a_41_NE2__a_40_O	5	3	7	9	7	17	3	17
a_41_NE2__a_41_O	0	0	1	1	0	1	0	1
a_41_NE2__a_42_O	6	8	10	6	4	0	3	0
a_41_NE2__a_42_OE1	1	0	0	0	0	0	0	0
a_41_NE2__a_43_OD1	1	0	2	2	0	1	0	2
a_41_NE2__a_43_OD2	1	0	1	0	0	1	0	1
a_41_NE2__a_48_O	7	7	8	4	1	6	7	1
a_41_NE2__a_48_OG1	13	26	7	20	2	2	4	3
a_41_NE2__a_50_OG	0	0	0	1	0	0	0	0
a_41_N__a_41_OE1	0	0	0	0	0	0	0	0
a_41_N__a_48_O	238	241	244	227	246	245	239	244
a_41_N__a_48_OG1	1	0	0	0	0	0	0	0
a_42_NE2__a_1_O	0	0	8	0	0	0	0	0
a_42_NE2__a_2_O	7	0	8	0	0	0	0	0
a_42_NE2__a_3_OG1	0	0	0	0	0	0	0	1
a_42_NE2__a_41_O	0	0	1	0	0	0	0	0
a_42_NE2__a_42_O	8	8	8	11	0	0	0	1
a_42_NE2__a_43_O	0	0	0	0	0	0	0	0
a_42_NE2__a_44_O	78	96	92	105	134	138	121	141
a_42_NE2__a_45_O	2	3	0	8	2	1	0	0
a_42_NE2__a_46_O	16	20	2	13	22	16	19	8
a_42_NE2__a_85_SD	0	0	1	0	1	7	1	3
a_42_N__a_2_O	1	1	31	17	24	58	36	43
a_42_N__a_3_OG1	1	9	6	3	63	32	8	26
a_42_N__a_40_O	0	0	0	0	0	0	0	0
a_42_N__a_41_OE1	44	32	33	18	40	21	29	24
a_42_N__a_42_OE1	6	5	6	1	0	0	0	0
a_43_N__a_41_O	0	0	0	0	0	0	0	0
a_43_N__a_44_O	0	0	0	0	0	0	0	0
a_43_N__a_46_O	231	239	234	207	113	92	112	127
a_44_N__a_42_O	2	0	1	0	0	0	0	0

a_44_N__a_42_OE1	4	5	6	1	0	0	0	2
a_44_N__a_43_OD1	0	0	0	0	1	0	0	2
a_44_N__a_43_OD2	0	0	0	0	0	0	1	0
a_44_N__a_46_O	0	0	0	13	0	0	0	5
a_45_NE2__a_43_OD2	0	0	0	0	0	0	0	3
a_45_NE2__a_44_O	0	1	4	2	1	1	1	4
a_45_NE2__a_45_O	1	4	3	4	2	1	0	1
a_45_NE2__a_46_OD1	0	0	2	0	0	0	0	1
a_45_NE2__a_46_OD2	0	0	1	0	0	0	2	0
a_45_NE2__a_64_OG1	0	0	0	0	0	0	0	1
a_45_NE2__a_65_O	57	50	75	36	121	116	95	69
a_45_NE2__a_85_SD	0	0	0	0	0	1	2	2
a_45_N__a_43_O	0	0	1	0	0	0	1	0
a_45_N__a_43_OD1	0	0	0	1	0	0	0	1
a_45_N__a_43_OD2	0	0	0	0	0	0	0	4
a_45_N__a_45_OE1	0	0	0	4	1	3	1	1
a_46_N__a_43_O	1	8	7	4	0	1	10	3
a_46_N__a_43_OD1	0	0	0	1	0	0	0	1
a_46_N__a_43_OD2	0	0	0	0	0	0	0	4
a_46_N__a_44_O	0	0	0	0	0	0	0	0
a_46_N__a_46_OD1	0	0	0	0	0	0	1	0
a_46_N__a_46_OD2	1	0	0	0	0	0	0	0
a_47_N__a_45_O	0	1	0	0	0	0	0	0
a_47_N__a_46_OD2	0	0	0	0	0	0	0	0
a_47_N__a_63_O	248	248	248	246	248	247	248	246
a_48_N__a_41_O	247	244	236	213	243	240	236	217
a_48_N__a_41_OE1	0	0	0	1	0	0	0	0
a_48_N__a_43_OD2	0	0	0	0	0	0	0	0
a_48_N__a_46_O	0	0	0	0	0	0	0	0
a_48_OG1__a_41_O	19	56	26	33	7	23	29	28
a_48_OG1__a_41_OE1	1	0	1	3	0	1	0	0
a_48_OG1__a_43_OD2	0	0	1	1	0	0	0	0
a_48_OG1__a_46_O	0	0	1	0	0	0	7	6
a_48_OG1__a_46_OD1	0	0	1	0	4	0	0	2
a_48_OG1__a_46_OD2	0	0	0	0	0	0	0	3
a_48_OG1__a_47_O	0	0	1	4	3	10	5	12
a_48_OG1__a_48_O	1	1	0	0	0	0	0	0
a_48_OG1__a_49_O	0	0	0	0	0	0	0	0
a_48_OG1__a_50_OG	0	0	0	0	0	0	0	0
a_48_OG1__a_60_OG1	0	0	0	0	0	0	0	0
a_48_OG1__a_61_O	0	0	12	11	28	52	19	44
a_49_NE1__a_110_OE1	0	0	1	0	0	0	0	0
a_49_NE1__a_110_OE2	0	1	0	0	0	0	0	0
**a_49_NE1__a_201_O**	**0**	**0**	**0**	**0**	**0**	**0**	**1**	**0**
**a_49_NE1__a_201_O2**	**0**	**0**	**0**	**0**	**0**	**15**	**29**	**22**
**a_49_NE1__a_201_O4**	**0**	**0**	**0**	**0**	**6**	**5**	**0**	**0**
**a_49_NE1__a_201_O5**	**0**	**0**	**0**	**0**	**4**	**3**	**0**	**0**
**a_49_NE1__a_202_O7**	**0**	**0**	**0**	**0**	**0**	**0**	**0**	**1**
a_49_NE1__a_38_OG1	4	14	25	29	10	4	38	18

Table S1 B

a_49_NE1__a_51_OE1	18	85	19	55	56	20	8	27
a_49_NE1__a_59_SD	0	0	1	0	0	0	0	0
a_49_NE1__a_61_OD1	0	0	0	0	17	26	29	16
a_49_NE1__a_73_O	0	0	0	0	0	0	0	0
a_49_NE1__a_74_SD	0	0	0	0	8	1	0	1
a_49_N__a_48_OG1	0	0	0	0	0	0	0	1
a_49_N__a_60_OG1	0	0	0	1	0	0	0	0
a_49_N__a_61_O	240	240	230	235	243	232	239	243
a_49_N__a_61_OD1	0	0	0	0	0	2	0	0
a_4_N__a_3_OG1	1	0	0	0	0	0	0	0
a_4_N__a_40_O	227	228	192	212	231	170	191	197
a_4_N__a_41_OE1	1	0	0	2	0	1	1	1
a_50_N__a_38_OG1	0	0	0	0	0	3	2	0
a_50_N__a_39_O	247	245	239	239	232	231	217	240
a_50_N__a_41_OE1	0	1	0	0	0	0	0	0
a_50_OG__a_39_O	22	24	60	56	40	30	29	31
a_50_OG__a_39_OE1	0	0	0	1	1	0	0	0
a_50_OG__a_39_OE2	0	0	0	1	0	0	0	0
a_50_OG__a_41_OE1	3	0	2	4	0	0	1	9
a_50_OG__a_48_OG1	0	0	2	0	0	0	0	0
a_50_OG__a_49_O	0	10	0	0	1	0	0	8
a_50_OG__a_50_O	0	0	0	0	0	0	0	0
a_50_OG__a_52_NE2	0	0	0	0	2	2	2	9
a_50_OG__a_58_O	0	0	0	0	0	0	0	0
a_50_OG__a_58_OG1	0	4	2	0	3	4	5	1
a_50_OG__a_59_O	3	11	4	8	23	57	31	19
a_50_OG__a_60_OG1	125	113	55	32	21	14	1	1
a_51_NE2__a_110_OE1	0	0	0	0	0	0	0	0
**a_51_NE2__a_201_N**	**0**	**0**	**0**	**0**	**2**	**1**	**4**	**3**
**a_51_NE2__a_201_O2**	**0**	**0**	**0**	**0**	**59**	**35**	**115**	**59**
**a_51_NE2__a_201_O4**	**0**	**0**	**0**	**0**	**5**	**0**	**0**	**2**
**a_51_NE2__a_201_O5**	**0**	**0**	**0**	**0**	**5**	**1**	**0**	**3**
a_51_NE2__a_37_O	2	19	7	3	0	0	0	0
a_51_NE2__a_38_OG1	3	13	46	51	13	8	1	16
a_51_NE2__a_50_O	5	4	0	0	0	0	0	0
a_51_NE2__a_51_O	0	0	0	0	2	1	1	2
a_51_NE2__a_52_O	0	0	0	0	0	0	0	3
a_51_NE2__a_53_OH	11	59	4	37	0	0	0	11
a_51_NE2__a_59_O	2	8	2	4	0	1	0	0
a_51_NE2__a_59_SD	0	5	0	2	1	4	0	1
a_51_NE2__a_72_OE1	0	2	0	0	0	0	0	0
a_51_NE2__a_73_O	3	0	6	0	0	0	0	0
a_51_NE2__a_74_SD	0	0	1	0	1	3	0	0
a_51_N__a_50_OG	0	0	0	0	0	0	0	0
a_51_N__a_51_OE1	0	0	0	3	0	12	18	19
a_51_N__a_58_OG1	0	1	4	0	0	0	0	0
a_51_N__a_59_O	245	244	245	241	240	210	220	231
a_52_ND1__a_37_O	0	0	0	0	2	8	5	7
a_52_ND1__a_39_OE1	0	0	0	0	8	8	8	0

a_52_ND1__a_50_O	0	0	0	0	3	3	8	1
a_52_ND1__a_50_OG	0	0	0	0	25	6	29	4
a_52_ND1__a_51_O	0	0	0	0	0	0	0	0
a_52_ND1__a_52_O	0	0	0	0	3	0	0	2
a_52_ND1__a_53_O	0	0	0	0	0	0	0	3
a_52_ND1__a_54_OG	0	0	0	0	0	0	2	0
a_52_ND1__a_57_O	0	0	0	0	0	4	0	25
a_52_ND1__a_58_OG1	0	0	0	0	23	22	6	47
a_52_NE2__a_39_OE1	60	12	35	10	0	0	0	0
a_52_NE2__a_39_OE2	44	8	22	5	0	0	0	0
a_52_NE2__a_50_OG	2	0	0	0	0	0	0	0
a_52_NE2__a_53_O	0	0	0	0	0	0	0	0
a_52_NE2__a_54_O	0	0	1	2	0	0	0	0
a_52_NE2__a_55_O	4	1	2	6	0	0	0	0
a_52_NE2__a_56_O	0	0	0	0	0	0	0	0
a_52_NE2__a_58_OG1	0	1	0	0	0	0	0	0
a_52_N__a_37_O	248	244	238	239	208	216	236	227
a_52_N__a_50_O	0	0	0	0	0	0	0	0
a_52_N__a_51_OE1	0	0	0	0	0	0	0	1
a_52_N__a_52_ND1	0	0	0	0	0	0	0	0
a_53_N__a_51_O	0	1	1	3	0	0	0	0
a_53_N__a_52_ND1	0	1	1	0	0	0	0	0
a_53_N__a_54_O	0	0	0	0	0	0	0	0
a_53_N__a_57_ND1	0	0	0	0	0	0	0	0
a_53_N__a_57_O	112	44	121	179	152	101	102	103
a_53_N__a_58_OG1	1	1	0	2	0	0	1	0
a_53_OH__a_14_OH	0	0	1	10	0	0	0	0
**a_53_OH__a_201_O2**	**0**	**0**	**0**	**0**	**0**	**0**	**0**	**3**
**a_53_OH__a_201_O4**	**0**	**0**	**0**	**0**	**1**	**0**	**0**	**7**
**a_53_OH__a_201_O5**	**0**	**0**	**0**	**0**	**0**	**0**	**0**	**6**
a_53_OH__a_26_OD1	0	0	0	0	0	0	3	6
a_53_OH__a_26_OD2	0	0	0	0	0	0	2	7
a_53_OH__a_27_O	0	0	3	2	0	1	0	1
a_53_OH__a_30_O	0	3	0	0	0	0	0	0
a_53_OH__a_31_O	0	0	1	0	0	0	0	0
a_53_OH__a_51_OE1	32	27	91	55	7	0	0	9
a_53_OH__a_57_ND1	4	11	11	0	1	0	2	1
a_53_OH__a_57_NE2	0	0	0	0	0	0	0	0
a_53_OH__a_58_O	0	0	2	0	0	0	0	0
a_53_OH__a_59_SD	3	5	7	1	0	0	0	0
a_53_OH__a_73_O	2	0	0	25	0	0	0	0
a_53_OH__a_74_O	0	0	0	3	0	0	0	0
a_53_OH__a_74_SD	8	0	4	4	0	0	1	0
a_53_OH__a_75_O	1	0	2	0	0	0	0	0
a_54_N__a_30_O	0	0	0	1	0	0	0	0
a_54_N__a_33_OD1	0	0	0	6	0	0	0	0
a_54_N__a_34_O	0	0	0	0	5	1	1	0
a_54_N__a_35_O	0	0	0	22	0	1	0	0
a_54_N__a_52_ND1	3	6	20	2	0	0	0	0

Table S1 B

a_54_N__a_52_O	0	0	0	2	0	0	0	0
a_54_N__a_57_O	75	20	41	32	0	0	0	0
a_54_OG__a_30_O	0	2	0	45	0	7	0	4
a_54_OG__a_31_O	0	1	0	1	0	0	0	0
a_54_OG__a_33_OD1	0	5	1	10	0	1	0	2
a_54_OG__a_34_O	0	0	0	9	18	10	2	0
a_54_OG__a_35_O	0	1	9	10	0	0	0	0
a_54_OG__a_53_O	16	5	7	4	0	0	3	0
a_54_OG__a_54_O	0	0	0	2	0	0	0	1
a_54_OG__a_55_O	0	0	0	1	0	0	0	0
a_54_OG__a_57_ND1	0	0	0	0	0	0	0	0
a_54_OG__a_57_O	2	6	7	0	0	0	0	0
a_55_N__a_52_ND1	0	1	2	2	0	0	0	0
a_55_N__a_53_O	0	0	0	4	116	103	49	18
a_55_N__a_54_OG	0	0	0	0	0	0	0	0
a_55_N__a_57_O	32	3	23	11	0	0	0	0
a_55_N__a_58_OG1	2	0	1	0	0	0	0	0
a_56_N__a_53_O	0	0	0	64	78	88	49	83
a_56_N__a_54_O	1	0	0	2	0	0	0	0
a_56_N__a_54_OG	130	71	102	22	0	0	0	0
a_56_N__a_57_O	0	1	0	0	0	0	0	0
a_57_NE2__a_14_OH	0	0	0	1	0	0	0	0
**a_57_NE2__a_201_O5**	**0**	**0**	**0**	**0**	**0**	**1**	**0**	**0**
a_57_NE2__a_27_O	0	0	1	0	0	0	0	0
a_57_NE2__a_30_O	1	0	1	0	0	0	0	0
a_57_NE2__a_51_OE1	0	0	0	0	22	23	39	18
a_57_NE2__a_53_OH	0	0	1	0	0	0	0	0
a_57_NE2__a_59_SD	0	0	0	0	3	3	2	2
a_57_NE2__a_73_O	0	0	0	11	0	0	0	0
a_57_NE2__a_74_O	0	0	0	39	0	0	0	1
a_57_NE2__a_74_SD	0	0	3	5	1	1	0	0
a_57_NE2__a_75_O	0	8	12	1	0	0	0	0
a_57_N__a_30_O	0	0	0	3	0	0	0	0
a_57_N__a_53_O	0	0	0	33	49	28	30	29
a_57_N__a_54_O	14	6	12	31	0	0	0	0
a_57_N__a_54_OG	118	57	94	15	0	0	0	0
a_57_N__a_55_O	0	0	0	0	0	0	1	0
a_58_N__a_56_O	3	1	3	2	0	0	0	1
a_58_N__a_57_ND1	5	2	0	3	3	4	5	1
a_58_N__a_58_OG1	23	11	54	68	12	16	3	13
a_58_OG1__a_50_OG	0	0	2	0	0	0	0	0
a_58_OG1__a_51_O	2	59	26	18	7	14	7	23
a_58_OG1__a_52_ND1	63	23	47	34	0	0	0	0
a_58_OG1__a_52_NE2	0	0	0	0	8	9	8	0
a_58_OG1__a_53_O	0	0	0	0	0	0	1	0
a_58_OG1__a_54_O	0	3	0	0	0	0	0	0
a_58_OG1__a_56_O	7	7	0	5	0	2	2	9
a_58_OG1__a_57_O	4	3	2	3	26	25	36	16
a_58_OG1__a_58_O	0	0	1	2	0	0	0	0

a_58_OG1__a_59_O	0	1	1	0	0	0	0	0
a_58_OG1__a_60_OG1	0	0	0	0	0	0	0	0
a_59_N__a_51_O	224	182	214	236	234	240	240	236
a_59_N__a_57_O	0	0	0	0	0	0	0	0
a_59_N__a_58_OG1	3	72	40	31	25	29	22	28
a_5_NZ__a_126_O	0	0	1	0	0	0	0	0
a_5_NZ__a_38_O	0	0	0	0	0	1	0	0
a_5_NZ__a_39_OE1	11	19	24	20	19	31	18	12
a_5_NZ__a_39_OE2	29	20	32	39	21	12	14	6
a_5_NZ__a_7_OE1	0	0	0	0	2	0	2	9
a_5_NZ__a_7_OE2	0	0	0	0	8	1	14	14
a_5_N__a_126_O	3	2	3	3	1	13	10	14
a_60_N__a_58_O	6	2	9	2	5	1	4	2
a_60_N__a_59_SD	3	3	4	8	8	3	9	12
a_60_N__a_60_O	154	191	165	210	134	155	147	167
a_60_N__a_60_OG1	25	18	20	20	75	61	103	61
a_60_N__a_72_OE1	10	24	13	27	0	0	0	0
a_60_N__a_74_O	0	0	0	0	0	3	7	2
a_60_OG1__a_48_OG1	0	0	0	1	0	0	0	0
a_60_OG1__a_49_O	9	9	21	33	0	4	0	25
a_60_OG1__a_50_OG	26	10	18	9	0	2	1	4
a_60_OG1__a_58_O	2	0	3	1	0	0	0	0
a_60_OG1__a_58_OG1	3	5	4	0	0	0	0	0
a_60_OG1__a_59_O	1	7	2	1	0	1	1	0
a_60_OG1__a_60_O	2	0	0	1	0	0	0	0
a_60_OG1__a_61_O	0	0	0	0	0	0	0	0
**a_61_ND2__a_201_O4**	**0**	**0**	**0**	**0**	**0**	**3**	**0**	**1**
**a_61_ND2__a_201_O5**	**0**	**0**	**0**	**0**	**0**	**3**	**0**	**4**
**a_61_ND2__a_202_O3**	**0**	**0**	**0**	**0**	**18**	**0**	**0**	**0**
**a_61_ND2__a_202_O7**	**0**	**0**	**0**	**0**	**18**	**26**	**20**	**28**
a_61_ND2__a_49_O	45	17	30	7	3	4	4	0
a_61_ND2__a_51_OE1	7	3	9	46	0	6	14	6
a_61_ND2__a_59_O	0	0	1	1	1	0	3	0
a_61_ND2__a_59_SD	0	2	1	4	13	0	3	0
a_61_ND2__a_60_O	0	0	0	0	0	0	2	0
a_61_ND2__a_61_O	5	0	0	1	0	3	0	0
a_61_ND2__a_62_O	2	4	5	3	0	1	12	0
a_61_ND2__a_69_OG	0	1	0	6	0	0	0	0
a_61_ND2__a_70_O	18	63	39	33	0	0	0	7
a_61_ND2__a_70_OD1	0	0	0	0	0	0	0	0
a_61_ND2__a_72_O	0	0	0	0	28	14	5	8
a_61_ND2__a_72_OE1	11	3	5	14	2	16	6	2
a_61_ND2__a_73_O	62	15	117	77	13	1	0	1
a_61_ND2__a_74_O	0	0	0	0	0	0	0	0
a_61_ND2__a_74_SD	0	0	0	0	51	0	0	7
a_61_N__a_49_O	225	238	227	239	228	224	226	229
a_61_N__a_59_O	0	0	0	0	0	0	0	0
a_61_N__a_60_OG1	0	0	0	0	0	0	0	0
a_61_N__a_61_OD1	0	0	0	0	0	0	0	0

Table S1 B

a_62_NZ__a_43_O	1	12	0	20	0	0	0	0
a_62_NZ__a_43_OD1	31	54	21	51	7	3	3	11
a_62_NZ__a_43_OD2	41	44	31	50	3	7	2	14
a_62_NZ__a_46_OD1	16	15	22	16	11	13	22	23
a_62_NZ__a_46_OD2	8	8	29	23	9	14	25	18
a_62_NZ__a_47_O	0	0	1	1	0	0	0	0
a_62_NZ__a_48_OG1	0	7	30	4	19	7	10	18
a_62_NZ__a_60_OG1	0	0	0	0	8	8	0	8
a_62_NZ__a_63_O	0	0	0	0	0	0	1	0
a_62_NZ__a_64_OG1	0	0	0	0	0	1	0	3
a_62_NZ__a_70_OD1	0	0	0	0	0	0	0	1
a_62_NZ__a_72_OE1	0	0	0	0	1	0	0	0
a_62_N__a_60_O	0	0	1	0	0	0	0	0
a_62_N__a_61_OD1	6	0	1	6	39	44	43	60
a_62_N__a_70_O	0	0	0	3	0	0	0	0
a_62_N__a_72_O	0	0	0	0	0	0	0	3
a_62_N__a_72_OE1	0	0	0	1	31	24	31	25
a_63_N__a_47_O	247	237	242	243	226	236	237	240
a_63_N__a_61_O	0	0	0	0	0	0	0	0
a_64_N__a_46_OD1	0	0	0	0	0	0	0	0
a_64_N__a_46_OD2	0	0	0	0	0	0	0	0
a_64_N__a_69_OG	199	215	208	214	0	0	0	0
a_64_N__a_70_O	0	0	0	0	9	0	9	7
a_64_N__a_70_OD1	0	0	0	0	179	226	215	220
a_64_OG1__a_45_O	3	1	17	4	11	5	10	18
a_64_OG1__a_46_OD1	98	109	82	84	107	95	104	87
a_64_OG1__a_46_OD2	96	83	70	97	73	103	89	87
a_64_OG1__a_63_O	0	1	1	1	0	0	1	0
a_64_OG1__a_64_O	0	0	1	1	0	0	0	1
a_64_OG1__a_67_O	0	8	3	4	4	0	0	0
a_64_OG1__a_68_O	4	1	0	1	0	0	0	0
a_64_OG1__a_69_OG	6	5	1	7	0	0	0	0
a_64_OG1__a_70_OD1	0	0	0	0	19	10	10	13
a_65_N__a_45_O	139	122	185	109	228	238	203	193
a_65_N__a_63_O	0	0	0	0	0	0	0	0
a_65_N__a_64_OG1	0	0	0	0	0	0	0	0
a_66_N__a_64_O	0	0	3	1	0	0	1	0
a_66_N__a_82_OG1	0	0	0	0	5	0	0	0
a_66_N__a_83_O	231	207	208	167	191	188	201	159
a_67_NZ__a_45_OE1	0	0	0	0	2	0	0	1
a_67_NZ__a_64_OG1	15	7	2	10	18	16	8	3
a_67_NZ__a_65_O	0	0	0	0	2	1	0	0
a_67_NZ__a_67_O	0	0	0	0	0	0	0	0
a_67_NZ__a_68_O	19	10	11	7	2	0	0	1
a_67_NZ__a_68_OE1	23	8	7	13	4	3	1	0
a_67_NZ__a_68_OE2	17	6	2	7	3	0	1	2
a_67_N__a_64_O	33	31	45	17	18	35	28	22
a_67_N__a_65_O	0	0	0	0	0	0	0	0
a_67_N__a_83_O	9	4	6	11	0	0	0	0

a_68_N__a_68_OE1	52	23	33	56	11	11	16	41
a_68_N__a_68_OE2	56	27	32	51	10	11	13	41
a_68_N__a_81_O	0	0	0	1	0	0	0	0
a_69_N__a_67_O	1	1	0	1	0	0	0	0
a_69_N__a_68_OE1	0	0	0	0	0	0	1	1
a_69_N__a_68_OE2	0	0	0	0	0	0	1	0
a_69_N__a_79_O	0	0	0	0	13	25	11	54
a_69_N__a_80_O	0	0	0	0	0	0	0	0
a_69_N__a_81_O	246	239	240	242	131	86	160	33
a_69_N__a_82_OG1	0	0	0	0	0	0	1	1
a_69_OG__a_61_OD1	0	0	0	4	0	0	0	0
a_69_OG__a_62_O	6	1	3	1	0	0	0	0
a_69_OG__a_64_O	6	6	6	15	0	0	0	0
a_69_OG__a_64_OG1	0	3	2	1	0	0	0	0
a_69_OG__a_67_O	37	65	49	30	0	0	0	0
a_69_OG__a_68_O	37	36	26	20	1	6	10	26
a_69_OG__a_68_OE1	0	0	0	0	23	9	25	27
a_69_OG__a_68_OE2	0	0	0	0	47	11	15	41
a_69_OG__a_69_O	0	0	0	0	17	10	11	11
a_69_OG__a_70_O	0	5	0	1	0	0	0	0
a_69_OG__a_78_OG1	0	0	0	0	4	8	27	5
a_69_OG__a_79_O	0	0	0	0	17	12	5	0
a_69_OG__a_81_O	8	0	2	0	0	0	0	0
a_6_N__a_38_O	248	248	247	247	223	236	239	244
a_6_N__a_38_OG1	0	0	0	0	1	0	0	0
a_6_N__a_4_O	0	0	0	0	0	1	0	0
a_70_ND2__a_61_OD1	0	7	2	0	0	0	0	0
a_70_ND2__a_62_O	0	0	0	0	4	0	0	0
a_70_ND2__a_64_OG1	0	0	0	0	17	3	2	8
a_70_ND2__a_67_O	0	0	0	0	0	2	0	3
a_70_ND2__a_68_O	0	0	0	0	11	10	15	21
a_70_ND2__a_68_OE1	0	0	0	1	0	0	0	0
a_70_ND2__a_69_O	0	0	0	0	0	0	0	1
a_70_ND2__a_70_O	1	0	0	0	3	1	0	0
a_70_ND2__a_72_OE1	1	0	14	0	0	0	0	0
a_70_ND2__a_78_OG1	25	36	19	31	0	0	0	0
a_70_N__a_62_O	0	0	0	4	0	0	0	0
a_70_N__a_67_O	0	0	0	0	6	5	7	0
a_70_N__a_68_O	0	0	0	0	2	0	0	0
a_70_N__a_69_OG	0	0	0	0	0	0	0	0
a_70_N__a_70_OD1	0	0	0	0	0	0	0	0
a_70_N__a_79_O	0	0	0	0	0	0	0	0
a_70_N__a_81_O	0	0	0	0	15	0	9	18
a_71_N__a_67_O	0	0	0	0	0	0	0	0
a_71_N__a_69_O	0	1	0	3	1	0	0	0
a_71_N__a_70_OD1	0	0	0	0	0	0	0	0
a_71_N__a_79_O	244	243	241	236	2	1	0	0
a_71_N__a_81_O	0	0	0	0	1	0	1	3
a_72_NE2__a_51_OE1	0	2	1	0	0	0	0	0

Table S1 B

a_72_NE2__a_59_SD	1	1	3	0	0	0	0	0
a_72_NE2__a_60_O	5	17	18	14	8	8	8	8
a_72_NE2__a_61_OD1	12	12	8	14	11	7	0	10
a_72_NE2__a_62_O	0	0	0	0	5	2	2	2
a_72_NE2__a_70_O	0	0	0	0	2	5	3	3
a_72_NE2__a_72_O	0	2	0	2	0	1	0	0
a_72_NE2__a_73_O	0	0	7	0	1	1	2	4
a_72_NE2__a_74_O	0	0	0	1	0	0	0	0
a_72_NE2__a_75_O	68	5	4	41	0	0	0	1
a_72_NE2__a_76_O	7	2	11	2	33	25	28	16
a_72_NE2__a_77_O	8	0	1	3	0	0	2	2
a_72_NE2__a_78_OG1	13	1	11	2	1	0	1	3
a_72_N__a_61_OD1	153	160	166	136	1	0	0	3
a_72_N__a_62_O	0	0	0	0	7	0	0	4
a_72_N__a_70_O	0	0	0	0	0	0	0	0
a_72_N__a_72_OE1	38	10	44	21	2	8	1	0
a_73_N__a_59_SD	0	0	0	12	0	0	0	0
a_73_N__a_61_OD1	149	45	162	144	0	0	0	0
a_73_N__a_71_O	11	13	54	15	0	0	0	1
a_73_N__a_72_OE1	0	0	4	1	0	1	1	2
a_73_N__a_73_O	0	0	0	0	0	1	3	2
a_73_N__a_73_OG1	0	0	0	2	247	246	242	242
a_73_N__a_76_O	0	0	0	0	0	0	1	0
a_73_N__a_77_O	0	0	0	0	248	248	248	248
**a_73_OG1__a_202_N25**	**0**	**0**	**0**	**0**	**1**	**0**	**0**	**0**
**a_73_OG1__a_202_OT1**	**0**	**0**	**0**	**0**	**26**	**29**	**37**	**32**
**a_73_OG1__a_202_OT2**	**0**	**0**	**0**	**0**	**42**	**29**	**38**	**26**
a_73_OG1__a_71_O	0	1	0	3	0	0	0	0
a_73_OG1__a_72_O	248	248	239	244	0	0	0	0
a_73_OG1__a_72_OE1	0	0	0	0	0	0	0	1
a_73_OG1__a_73_O	0	0	0	1	4	8	4	8
a_73_OG1__a_74_O	6	2	1	0	0	0	0	1
a_73_OG1__a_75_O	0	0	0	16	0	0	0	0
a_73_OG1__a_76_O	0	0	0	0	0	0	0	0
a_73_OG1__a_77_O	31	193	174	55	162	194	187	171
a_73_OG1__a_78_O	0	0	0	0	1	0	0	0
a_73_OG1__a_97_OH	0	0	0	0	0	0	0	0
**a_74_N__a_202_N25**	**0**	**0**	**0**	**0**	**2**	**5**	**2**	**3**
**a_74_N__a_202_O24**	**0**	**0**	**0**	**0**	**66**	**123**	**127**	**121**
**a_74_N__a_202_OT1**	**0**	**0**	**0**	**0**	**16**	**26**	**11**	**20**
**a_74_N__a_202_OT2**	**0**	**0**	**0**	**0**	**32**	**17**	**7**	**20**
a_74_N__a_72_O	58	9	10	58	0	0	0	0
a_74_N__a_73_OG1	0	0	0	0	0	0	0	0
a_74_N__a_74_SD	0	0	0	2	3	17	7	16
a_74_N__a_75_O	0	0	0	9	0	0	0	0
a_74_N__a_97_OH	0	2	0	8	0	0	0	0
**a_75_N__a_202_O24**	**0**	**0**	**0**	**0**	**1**	**7**	**0**	**1**
**a_75_N__a_202_OT1**	**0**	**0**	**0**	**0**	**16**	**14**	**16**	**14**
**a_75_N__a_202_OT2**	**0**	**0**	**0**	**0**	**4**	**10**	**12**	**8**

Table S1 B

a_75_N__a_53_OH	1	1	41	0	0	0	0	0
a_75_N__a_59_SD	5	0	5	0	0	0	0	0
a_75_N__a_72_O	0	0	0	151	0	0	0	0
a_75_N__a_72_OE1	0	0	0	4	0	0	0	0
a_75_N__a_73_O	8	0	8	1	0	0	0	1
a_75_N__a_73_OG1	0	0	0	10	61	47	58	58
a_75_N__a_74_SD	1	2	3	1	0	1	0	0
a_75_N__a_75_O	26	9	5	44	0	0	0	3
a_76_N__a_72_O	0	2	22	7	0	0	0	0
a_76_N__a_72_OE1	0	0	0	4	0	0	0	0
a_76_N__a_73_O	0	0	0	0	163	163	181	145
a_76_N__a_73_OG1	0	0	0	1	47	44	43	58
a_76_N__a_74_O	0	0	0	0	0	0	0	0
**a_77_NZ__a_202_N25**	**0**	**0**	**0**	**0**	**0**	**0**	**0**	**0**
**a_77_NZ__a_202_O24**	**0**	**0**	**0**	**0**	**2**	**0**	**0**	**1**
**a_77_NZ__a_202_OT1**	**0**	**0**	**0**	**0**	**1**	**1**	**7**	**5**
**a_77_NZ__a_202_OT2**	**0**	**0**	**0**	**0**	**2**	**3**	**6**	**6**
a_77_NZ__a_21_O	0	5	44	7	0	0	0	0
a_77_NZ__a_22_O	7	1	5	1	0	0	0	0
a_77_NZ__a_26_OD2	0	0	0	0	0	0	0	0
a_77_NZ__a_73_OG1	0	0	0	0	0	1	0	0
a_77_NZ__a_75_O	0	0	0	1	6	1	2	5
a_77_NZ__a_78_O	6	3	0	1	0	1	0	2
**a_77_N__a_202_OT1**	**0**	**0**	**0**	**0**	**1**	**0**	**0**	**1**
a_77_N__a_72_O	0	11	41	10	0	0	0	0
a_77_N__a_73_OG1	0	0	0	0	116	75	97	110
a_77_N__a_75_O	3	23	7	38	62	94	75	69
a_78_N__a_72_OE1	0	0	0	1	0	0	0	0
a_78_N__a_76_O	1	1	1	1	0	0	0	0
a_78_OG1__a_69_O	0	0	0	0	0	1	4	5
a_78_OG1__a_69_OG	0	0	0	0	0	0	0	3
a_78_OG1__a_70_OD1	7	1	2	6	0	0	0	0
a_78_OG1__a_71_O	0	0	7	0	1	0	9	0
a_78_OG1__a_72_OE1	9	2	11	21	2	4	6	5
a_78_OG1__a_76_O	1	4	4	4	1	0	0	1
a_78_OG1__a_77_O	0	0	0	29	0	0	0	0
a_78_OG1__a_78_O	0	0	1	0	0	0	0	0
a_79_N__a_70_OD1	0	0	4	0	0	0	0	0
a_79_N__a_71_O	247	240	235	243	148	183	158	170
a_79_N__a_77_O	0	0	2	0	0	0	0	0
a_79_N__a_78_OG1	0	0	0	0	0	0	0	0
a_7_N__a_123_OG	0	0	0	0	0	0	0	3
a_7_N__a_124_O	248	246	248	246	220	232	228	203
a_80_NZ__a_68_O	0	0	0	0	0	0	0	0
a_80_NZ__a_68_OE1	25	45	48	20	58	31	42	40
a_80_NZ__a_68_OE2	18	29	30	16	52	32	45	44
a_80_NZ__a_69_OG	0	0	0	0	33	43	42	70
a_80_NZ__a_70_OD1	0	2	10	7	0	0	0	0
a_80_NZ__a_78_O	0	0	0	0	0	0	0	0

a_80_NZ__a_78_OG1	0	0	0	1	3	9	6	13
**a_80_N__a_202_OT1**	**0**	**0**	**0**	**0**	**1**	**0**	**0**	**0**
a_80_N__a_78_O	0	0	0	0	0	0	0	0
a_81_N__a_68_OE1	0	0	0	0	0	1	1	0
a_81_N__a_68_OE2	0	0	0	0	0	0	0	0
a_81_N__a_69_O	248	247	246	246	0	0	0	0
a_81_N__a_69_OG	0	0	0	0	1	0	0	0
a_81_N__a_79_O	24	4	32	72	233	205	237	229
a_81_N__a_81_O	211	228	198	159	180	146	151	164
a_82_N__a_93_O	80	31	61	87	52	16	45	12
a_82_N__a_93_OD1	1	0	20	15	0	0	0	0
a_82_OG1__a_66_O	0	0	0	0	0	0	0	0
a_82_OG1__a_67_O	2	2	0	0	15	10	4	14
a_82_OG1__a_68_OE1	0	0	0	0	2	10	6	2
a_82_OG1__a_68_OE2	0	0	0	0	4	4	6	0
a_82_OG1__a_80_O	0	0	0	0	0	1	0	0
a_82_OG1__a_81_O	0	0	0	1	2	0	0	0
a_82_OG1__a_82_O	0	3	3	4	0	0	0	1
a_82_OG1__a_83_O	0	0	0	0	2	0	0	0
a_82_OG1__a_84_OE1	12	5	15	7	12	27	51	55
a_82_OG1__a_93_O	6	1	7	5	5	3	7	3
a_82_OG1__a_93_OD1	35	6	40	54	0	0	1	9
a_83_N__a_66_O	0	0	0	1	0	0	0	0
a_83_N__a_67_O	99	77	81	78	7	4	25	3
a_83_N__a_81_O	0	1	0	2	0	1	0	2
a_83_N__a_82_OG1	0	0	0	0	0	0	0	0
a_84_NE2__a_66_O	0	1	0	0	0	0	0	0
a_84_NE2__a_82_O	0	1	0	1	8	8	8	0
a_84_NE2__a_82_OG1	0	0	1	0	5	1	4	9
a_84_NE2__a_83_O	0	0	0	2	0	0	0	0
a_84_NE2__a_84_O	0	0	0	0	0	0	0	0
a_84_NE2__a_85_O	2	1	2	3	3	0	4	1
a_84_NE2__a_86_OE1	5	4	4	2	4	1	0	6
a_84_NE2__a_86_OE2	2	7	3	4	2	3	1	3
a_84_NE2__a_91_O	32	20	50	39	27	56	55	52
a_84_N__a_82_O	0	0	0	0	0	0	0	0
a_84_N__a_84_OE1	0	0	0	0	1	2	1	0
a_84_N__a_91_O	246	243	243	247	229	218	184	220
a_85_N__a_83_O	0	1	0	0	0	0	0	0
a_85_N__a_84_OE1	12	9	8	10	14	2	6	9
a_86_N__a_84_O	0	0	0	0	0	0	0	0
a_86_N__a_85_SD	4	7	5	4	2	0	0	1
a_86_N__a_86_O	26	11	18	20	34	40	54	33
a_86_N__a_86_OE1	0	1	0	0	0	0	0	0
a_86_N__a_89_O	246	223	233	236	242	239	246	240
a_87_N__a_85_O	0	0	0	0	1	1	0	2
a_87_N__a_85_SD	11	36	24	25	1	1	2	3
a_87_N__a_86_OE1	3	7	2	3	0	1	0	0
a_87_N__a_86_OE2	6	1	1	2	0	0	0	0

Table S1 B

a_87_N__a_89_O	1	17	7	22	0	0	2	0
a_88_N__a_85_O	0	0	1	0	0	0	0	3
a_88_N__a_85_SD	10	1	6	0	12	7	2	10
a_88_N__a_86_O	76	46	66	65	13	45	47	29
a_88_N__a_86_OE1	0	17	0	2	0	0	0	0
a_88_N__a_86_OE2	0	0	0	2	0	0	1	0
a_88_N__a_89_O	0	0	0	0	0	0	0	1
a_89_NZ__a_100_OG1	0	0	0	0	0	1	0	2
a_89_NZ__a_101_O	0	0	0	0	14	12	58	13
a_89_NZ__a_102_OE1	19	24	20	24	51	78	41	83
a_89_NZ__a_102_OE2	13	30	19	21	51	72	65	73
a_89_NZ__a_86_O	14	0	8	2	20	16	15	14
a_89_NZ__a_86_OE1	0	1	5	6	1	10	4	1
a_89_NZ__a_86_OE2	6	10	19	12	4	2	2	1
a_89_N__a_85_SD	0	1	1	1	0	0	0	0
a_89_N__a_86_O	60	20	55	35	33	49	75	43
a_89_N__a_86_OE1	0	12	0	0	0	0	0	0
a_89_N__a_86_OE2	0	0	0	2	0	0	1	0
a_89_N__a_87_O	0	0	0	0	0	0	0	0
a_8_N__a_36_O	92	192	208	209	120	102	153	111
a_8_N__a_7_OE1	14	0	0	5	20	4	0	0
a_8_N__a_7_OE2	3	0	0	3	13	8	0	0
a_8_N__a_8_SD	1	1	1	7	0	0	1	0
a_90_N__a_101_O	245	244	244	238	64	20	59	27
a_90_N__a_101_OG	0	0	0	3	35	112	25	91
**a_90_N__a_201_O**	**0**	**0**	**0**	**0**	**35**	**19**	**55**	**31**
a_91_N__a_84_O	247	242	244	245	245	242	232	237
a_92_N__a_99_O	243	236	242	231	197	188	158	172
a_92_N__a_99_OE1	0	0	1	0	0	1	32	16
a_93_ND2__a_82_O	0	0	0	0	0	0	0	0
a_93_ND2__a_82_OG1	7	1	5	10	3	1	1	0
a_93_ND2__a_84_OE1	0	0	0	1	11	11	12	15
a_93_ND2__a_92_O	0	0	0	0	0	0	0	0
a_93_ND2__a_93_O	1	3	0	0	3	0	0	1
a_93_ND2__a_94_O	0	0	0	0	0	0	0	0
a_93_ND2__a_97_O	0	0	0	0	1	0	0	0
a_93_ND2__a_98_ND1	36	75	39	21	3	5	0	0
a_93_ND2__a_98_NE2	0	0	0	0	0	1	0	0
a_93_N__a_82_O	117	31	74	130	74	24	58	27
a_93_N__a_82_OG1	0	0	0	0	0	0	0	0
a_93_N__a_91_O	0	0	0	0	0	0	0	0
a_93_N__a_93_OD1	1	3	2	1	0	0	0	0
a_94_N__a_92_O	1	1	0	0	0	0	0	1
a_94_N__a_93_OD1	0	0	0	0	0	0	1	4
a_94_N__a_97_O	234	225	225	234	173	227	193	210
a_96_ND2__a_114_O	0	0	0	0	0	0	4	6
**a_96_ND2__a_202_N25**	**0**	**0**	**0**	**0**	**0**	**1**	**0**	**0**
**a_96_ND2__a_202_O24**	**0**	**0**	**0**	**0**	**0**	**1**	**0**	**1**
a_96_ND2__a_20_O	14	0	29	5	0	4	0	0

Table S1 B

a_96_ND2__a_21_O	102	8	9	19	0	0	0	0
a_96_ND2__a_73_OG1	22	24	5	4	0	0	0	0
a_96_ND2__a_74_O	0	10	7	0	0	0	0	0
a_96_ND2__a_74_SD	0	0	0	6	0	0	0	0
a_96_ND2__a_95_O	0	0	0	0	2	1	1	0
a_96_ND2__a_96_O	0	0	4	3	18	20	18	6
a_96_N__a_94_O	27	34	8	10	5	1	5	0
a_96_N__a_96_OD1	2	12	2	9	0	0	0	0
a_96_N__a_98_NE2	0	0	0	0	0	0	10	0
a_97_N__a_94_O	52	36	51	64	166	173	166	154
a_97_N__a_95_O	0	0	0	0	0	0	0	0
a_97_N__a_96_OD1	34	52	20	14	0	0	0	0
a_97_OH__a_112_OG	0	0	10	0	2	6	7	4
a_97_OH__a_119_OH	0	0	0	0	0	0	0	13
**a_97_OH__a_201_O2**	**0**	**0**	**0**	**0**	**0**	**4**	**0**	**0**
**a_97_OH__a_202_O7**	**0**	**0**	**0**	**0**	**13**	**3**	**0**	**6**
a_97_OH__a_21_O	0	0	0	1	0	0	0	0
a_97_OH__a_73_O	1	0	0	0	0	0	0	0
a_97_OH__a_73_OG1	6	2	1	7	0	0	0	0
a_97_OH__a_74_SD	0	0	2	2	0	0	0	0
a_97_OH__a_99_OE1	11	11	13	19	194	149	136	129
a_98_ND1__a_113_O	0	0	0	0	0	0	1	1
a_98_ND1__a_113_OG1	0	0	0	0	5	0	4	0
a_98_ND1__a_92_O	0	0	0	0	124	178	142	145
a_98_ND1__a_93_OD1	0	0	0	0	55	27	33	23
a_98_ND1__a_94_O	0	0	0	0	0	2	0	6
a_98_ND1__a_96_O	0	0	0	0	0	0	0	4
a_98_ND1__a_97_O	0	0	0	0	0	1	2	2
a_98_ND1__a_98_O	0	0	0	0	0	0	0	0
a_98_ND1__a_99_O	0	0	0	0	0	2	1	3
a_98_NE2__a_100_OG1	2	3	2	3	0	0	0	0
a_98_NE2__a_93_OD1	1	0	0	0	0	0	0	0
a_98_N__a_113_O	241	232	226	239	214	206	227	213
a_98_N__a_113_OG1	0	0	0	0	0	0	0	1
a_98_N__a_96_O	0	0	0	0	0	0	0	0
a_99_NE2__a_100_O	1	0	0	0	1	0	0	2
a_99_NE2__a_101_OG	1	1	4	6	12	15	11	11
a_99_NE2__a_110_OE1	18	30	22	14	78	74	135	75
a_99_NE2__a_110_OE2	23	9	23	12	115	63	66	87
a_99_NE2__a_111_O	1	5	0	0	0	0	0	0
a_99_NE2__a_112_OG	49	51	43	49	0	1	1	1
**a_99_NE2__a_201_O**	**0**	**0**	**0**	**0**	**0**	**1**	**3**	**7**
**a_99_NE2__a_201_O1**	**0**	**0**	**0**	**0**	**0**	**0**	**0**	**1**
**a_99_NE2__a_201_O3**	**0**	**0**	**0**	**0**	**0**	**0**	**0**	**0**
**a_99_NE2__a_202_O3**	**0**	**0**	**0**	**0**	**2**	**1**	**2**	**1**
**a_99_NE2__a_202_O7**	**0**	**0**	**0**	**0**	**0**	**0**	**0**	**0**
a_99_NE2__a_90_O	0	0	0	0	0	17	0	2
a_99_NE2__a_97_OH	0	0	2	1	0	2	2	6
a_99_NE2__a_98_O	0	0	0	0	0	0	0	0

Table S1 B

a_99_NE2__a_99_O	0	0	0	0	0	0	0	0
a_99_N__a_92_O	246	241	236	240	157	186	147	171
a_99_N__a_98_ND1	1	2	0	1	0	0	1	0
a_99_N__a_99_OE1	0	0	0	0	0	0	1	0
a_9_N__a_10_O	0	0	0	0	2	0	0	0
a_9_N__a_11_OE1	0	0	0	0	0	0	0	0
a_9_N__a_11_OE2	0	0	0	0	0	1	0	0
a_9_N__a_122_O	245	248	235	225	247	245	227	231
a_9_N__a_123_OG	0	0	0	0	0	0	0	0
a_9_N__a_7_O	1	3	12	0	0	1	0	1
a_9_N__a_8_SD	1	3	2	4	2	4	4	6
a_9_N__a_9_O	0	0	0	0	68	48	174	68
a_9_N__a_9_OE2	0	0	0	1	0	0	0	0

Table S1 B

Table S1 C

	4.1							
	Apo 283	*Apo 291*	*Apo 298*	*Apo 313*	*Holo 28*	*Holo 291*	*Holo 29*	*Holo 31*
a_100_N__a_110_OE1	0	0	0	0	0	0	3	0
a_100_N__a_111_O	248	248	245	244	248	245	245	246
a_100_N__a_99_OE1	6	9	14	12	1	0	0	1
a_100_OG1__a_100_O	49	52	36	52	62	47	70	68
a_100_OG1__a_101_O	0	0	0	0	0	4	0	8
a_100_OG1__a_111_O	27	55	47	51	18	42	15	44
a_100_OG1__a_90_O	7	10	17	6	32	9	27	18
a_100_OG1__a_98_ND1	0	1	1	0	0	0	0	0
a_100_OG1__a_98_NE2	3	2	1	2	2	1	2	1
a_100_OG1__a_99_O	53	50	58	42	12	4	13	5
a_101_N__a_100_OG1	3	5	6	0	28	8	9	10
a_101_N__a_90_O	247	247	246	244	139	114	141	143
a_101_N__a_99_OE1	1	1	4	0	0	0	0	0
a_101_OG__a_100_O	5	2	10	3	0	0	0	0
a_101_OG__a_101_O	1	1	5	9	0	0	0	0
a_101_OG__a_109_O	55	114	73	40	32	2	1	5
a_101_OG__a_110_OE1	1	2	3	3	15	12	33	10
a_101_OG__a_110_OE2	4	2	4	3	83	26	124	18
**a_101_OG__a_201_O**	**0**	**0**	**0**	**0**	**20**	**80**	**0**	**24**
**a_101_OG__a_201_O1**	**0**	**0**	**0**	**0**	**51**	**10**	**37**	**38**
**a_101_OG__a_201_O3**	**0**	**0**	**0**	**0**	**0**	**0**	**0**	**1**
a_101_OG__a_90_O	19	16	16	45	32	78	27	82
a_101_OG__a_99_OE1	63	62	53	51	1	4	1	9
a_102_N__a_100_O	0	0	0	1	0	0	0	1
a_102_N__a_101_OG	8	26	12	8	9	0	0	2
a_102_N__a_109_O	245	246	244	243	245	242	210	234
**a_102_N__a_201_O**	**0**	**0**	**0**	**0**	**0**	**1**	**0**	**0**
**a_102_N__a_201_O1**	**0**	**0**	**0**	**0**	**1**	**0**	**0**	**6**
a_103_N__a_102_OE1	3	0	1	1	63	59	45	58
a_103_N__a_102_OE2	4	0	1	2	70	73	46	71
a_103_N__a_88_O	47	186	134	157	0	1	0	1
a_104_N__a_102_O	3	5	8	5	0	0	0	0
a_104_N__a_107_O	247	245	243	247	248	245	247	244
a_105_N__a_103_O	3	0	0	1	6	1	0	1
a_105_N__a_107_O	25	77	85	55	0	2	6	20
a_106_N__a_104_O	2	2	5	3	0	0	2	4
a_106_N__a_106_OD1	9	2	10	22	8	16	32	6
a_106_N__a_106_OD2	7	6	15	33	7	24	29	15
a_107_NZ__a_106_OD1	2	6	2	7	1	5	9	1
a_107_NZ__a_106_OD2	0	4	1	1	2	7	8	6
a_107_NZ__a_10_OG	0	0	0	0	0	0	2	0
a_107_NZ__a_120_OE2	0	0	0	0	0	0	0	1
a_107_NZ__a_9_OE1	10	8	6	15	10	33	30	26
a_107_NZ__a_9_OE2	4	8	2	8	18	30	28	27
a_107_N__a_104_O	0	0	0	3	8	3	9	10
a_107_N__a_105_O	24	23	16	32	0	0	0	4
a_107_N__a_106_OD1	0	0	0	0	0	2	3	0

Table S1 C

a_107_N__a_106_OD2	0	0	0	0	0	1	2	5
a_108_N__a_123_O	248	248	248	248	244	246	246	237
a_108_N__a_123_OG	0	0	0	0	0	0	1	2
a_109_N__a_102_O	248	247	247	248	243	245	244	239
a_10_N__a_11_OE1	0	0	0	0	1	6	19	14
a_10_N__a_11_OE2	0	0	0	0	5	15	44	20
a_10_N__a_122_O	183	210	145	122	1	145	0	95
a_10_N__a_8_SD	0	2	0	2	3	0	0	0
a_10_N__a_9_OE1	0	0	2	1	0	1	15	0
a_10_N__a_9_OE2	1	0	1	0	0	2	15	1
a_10_OG__a_10_O	1	0	0	0	0	4	9	5
a_10_OG__a_11_O	0	0	0	2	0	0	1	0
a_10_OG__a_11_OE1	0	0	0	0	2	0	0	1
a_10_OG__a_11_OE2	0	0	0	0	2	0	0	2
a_10_OG__a_120_O	0	0	0	0	0	0	0	1
a_10_OG__a_122_O	0	0	1	1	0	0	0	0
a_10_OG__a_9_O	1	6	7	6	63	12	11	21
a_10_OG__a_9_OE1	116	103	82	83	2	55	64	55
a_10_OG__a_9_OE2	105	84	103	80	1	61	69	43
a_110_N__a_121_O	248	247	247	246	247	247	199	246
a_111_N__a_100_O	244	247	244	248	244	244	238	241
a_111_N__a_110_OE2	0	0	0	0	0	0	4	1
a_111_N__a_99_OE1	0	0	0	1	0	0	0	0
a_112_N__a_119_O	248	245	245	234	243	247	246	242
a_112_OG__a_110_O	1	0	0	0	0	2	1	3
a_112_OG__a_110_OE1	1	7	1	0	1	1	10	4
a_112_OG__a_110_OE2	0	9	0	0	3	0	0	6
a_112_OG__a_111_O	9	9	4	4	0	0	3	3
a_112_OG__a_112_O	3	1	5	2	7	2	15	1
a_112_OG__a_119_O	9	2	16	11	26	22	71	25
a_112_OG__a_119_OH	0	0	0	5	0	3	0	3
**a_112_OG__a_201_O2**	**0**	**0**	**0**	**0**	**0**	**1**	**0**	**0**
a_112_OG__a_97_OH	0	0	0	0	19	19	18	12
a_112_OG__a_98_O	4	22	7	16	1	39	9	11
a_112_OG__a_99_OE1	43	31	33	34	10	34	13	13
a_113_N__a_112_OG	0	2	1	1	1	10	1	6
a_113_N__a_98_O	248	243	245	248	247	232	246	245
a_113_OG1__a_111_O	2	0	0	0	0	0	0	0
a_113_OG1__a_112_O	7	3	1	7	10	10	15	21
a_113_OG1__a_113_O	13	12	15	16	40	41	32	40
a_113_OG1__a_114_O	0	2	2	0	0	0	0	0
a_113_OG1__a_115_O	0	0	0	0	0	0	0	2
a_113_OG1__a_117_O	0	2	15	13	0	14	0	15
a_113_OG1__a_118_OG1	25	35	89	34	82	58	59	15
a_113_OG1__a_98_ND1	1	0	1	3	0	0	1	0
a_113_OG1__a_98_O	18	18	11	20	21	13	29	38
a_114_N__a_113_OG1	0	9	34	12	0	2	0	3
a_114_N__a_117_O	233	196	213	209	243	246	237	236
a_114_N__a_118_OG1	2	0	0	0	0	0	0	0

Table S1 C

a_115_N__a_113_O	0	0	0	0	3	2	1	4
a_115_N__a_113_OG1	0	0	0	20	0	0	0	0
a_115_N__a_117_O	0	0	0	4	3	0	0	0
a_115_N__a_96_O	0	0	0	0	61	17	51	30
a_115_N__a_96_OD1	0	0	0	0	10	4	12	17
a_116_N__a_113_OG1	0	0	0	3	0	7	0	6
a_116_N__a_114_O	74	45	53	15	46	21	34	28
a_116_N__a_117_O	0	0	0	0	0	1	0	0
a_117_N__a_114_O	44	11	10	30	189	82	134	146
a_117_N__a_115_O	3	3	1	2	1	0	1	0
a_118_N__a_116_O	0	0	0	0	0	0	0	2
a_118_OG1__a_112_O	10	2	1	19	17	0	0	4
a_118_OG1__a_113_OG1	2	17	12	11	17	5	20	7
a_118_OG1__a_114_O	1	0	0	0	0	0	0	0
a_118_OG1__a_116_O	0	0	0	35	41	71	64	14
a_118_OG1__a_117_O	43	29	42	11	11	2	5	1
a_118_OG1__a_118_O	2	2	2	3	6	6	3	6
a_118_OG1__a_119_O	0	0	0	2	0	0	0	0
a_118_OG1__a_13_OD1	0	0	0	0	0	0	0	1
a_119_N__a_112_O	247	248	245	234	247	247	245	229
a_119_N__a_112_OG	0	0	0	0	1	0	0	8
a_119_N__a_118_OG1	5	13	2	11	14	0	0	1
a_119_OH__a_110_OE1	0	1	1	14	0	0	0	7
a_119_OH__a_110_OE2	0	0	0	1	0	0	0	3
a_119_OH__a_112_OG	0	0	0	0	0	1	0	2
a_119_OH__a_11_OE1	22	14	58	56	35	16	0	35
a_119_OH__a_11_OE2	95	102	114	105	6	35	0	4
a_119_OH__a_12_O	0	0	0	11	0	12	26	4
a_119_OH__a_13_O	0	0	0	1	0	7	0	1
a_119_OH__a_14_OH	0	0	0	0	8	0	0	0
**a_119_OH__a_201_N**	**0**	**0**	**0**	**0**	**0**	**0**	**0**	**1**
**a_119_OH__a_201_O2**	**0**	**0**	**0**	**0**	**0**	**6**	**0**	**20**
**a_119_OH__a_201_O4**	**0**	**0**	**0**	**0**	**10**	**7**	**93**	**28**
**a_119_OH__a_201_O5**	**0**	**0**	**0**	**0**	**23**	**10**	**82**	**23**
a_119_OH__a_74_SD	0	4	0	0	0	0	0	0
a_119_OH__a_8_SD	0	0	0	0	1	1	3	0
a_119_OH__a_97_OH	0	0	0	1	0	0	0	3
a_119_OH__a_99_OE1	0	0	0	0	0	0	0	2
a_11_N__a_10_OG	3	15	17	23	82	24	0	29
a_11_N__a_11_OE1	29	23	15	4	23	38	127	47
a_11_N__a_11_OE2	16	14	15	5	31	46	100	76
a_120_N__a_120_OE1	0	0	0	2	0	0	3	0
a_120_N__a_120_OE2	1	0	0	2	0	1	0	0
a_120_N__a_12_O	29	22	0	56	215	188	142	166
a_120_N__a_13_OD1	0	0	0	4	0	0	0	5
a_121_NE__a_110_OE1	0	1	32	30	0	0	0	0
a_121_NE__a_110_OE2	0	1	42	49	0	0	0	16
a_121_NE__a_112_OG	0	0	0	0	0	1	1	0
a_121_NE__a_119_O	0	0	0	0	0	1	0	0

Table S1 C

a_121_NE__a_119_OH	32	87	55	39	110	104	12	68
a_121_NE__a_11_OE1	13	0	1	14	0	0	0	1
a_121_NE__a_11_OE2	20	15	2	1	0	10	0	5
a_121_NE__a_123_OG	1	1	0	0	0	0	0	0
a_121_NE__a_14_OH	1	0	1	1	0	0	0	0
a_121_NE__a_16_OE1	0	0	0	0	0	0	0	4
a_121_NE__a_16_OE2	0	0	0	0	0	0	0	2
**a_121_NE__a_201_N**	**0**	**0**	**0**	**0**	**4**	**0**	**29**	**0**
**a_121_NE__a_201_O1**	**0**	**0**	**0**	**0**	**0**	**0**	**0**	**4**
**a_121_NE__a_201_O2**	**0**	**0**	**0**	**0**	**5**	**7**	**16**	**20**
**a_121_NE__a_201_O4**	**0**	**0**	**0**	**0**	**6**	**4**	**57**	**6**
**a_121_NE__a_201_O5**	**0**	**0**	**0**	**0**	**17**	**3**	**34**	**8**
a_121_NE__a_74_SD	0	2	7	0	0	0	0	0
a_121_NE__a_8_SD	29	12	4	9	13	16	0	10
a_121_NH1__a_110_OE1	45	72	78	61	159	150	139	134
a_121_NH1__a_110_OE2	125	150	84	70	57	59	38	60
a_121_NH1__a_112_OG	0	0	0	0	6	0	19	2
a_121_NH1__a_119_OH	2	13	8	8	0	0	0	1
a_121_NH1__a_11_OE1	0	0	0	7	0	0	0	0
a_121_NH1__a_11_OE2	0	0	0	6	0	0	0	0
a_121_NH1__a_121_O	0	0	0	1	0	0	0	0
a_121_NH1__a_123_OG	11	9	12	9	0	0	0	0
a_121_NH1__a_14_OH	0	0	11	2	0	0	0	0
**a_121_NH1__a_201_N**	**0**	**0**	**0**	**0**	**1**	**0**	**9**	**8**
**a_121_NH1__a_201_O1**	**0**	**0**	**0**	**0**	**3**	**0**	**7**	**0**
**a_121_NH1__a_201_O2**	**0**	**0**	**0**	**0**	**9**	**16**	**0**	**26**
**a_121_NH1__a_201_O3**	**0**	**0**	**0**	**0**	**25**	**72**	**50**	**30**
**a_121_NH1__a_201_O4**	**0**	**0**	**0**	**0**	**1**	**0**	**12**	**10**
**a_121_NH1__a_201_O5**	**0**	**0**	**0**	**0**	**1**	**1**	**1**	**9**
**a_121_NH1__a_202_O3**	**0**	**0**	**0**	**0**	**2**	**33**	**47**	**24**
**a_121_NH1__a_202_O7**	**0**	**0**	**0**	**0**	**9**	**14**	**38**	**11**
a_121_NH1__a_51_OE1	1	9	10	5	0	0	0	0
a_121_NH1__a_53_OH	0	0	2	10	0	0	0	0
a_121_NH1__a_73_O	0	0	2	0	0	0	0	0
a_121_NH1__a_74_SD	0	4	13	4	0	0	0	0
a_121_NH1__a_8_SD	2	0	1	0	0	0	0	0
a_121_NH1__a_97_OH	0	0	0	0	1	0	3	3
a_121_NH1__a_99_OE1	0	0	0	0	3	8	7	1
a_121_NH2__a_110_OE1	7	43	74	85	3	0	0	13
a_121_NH2__a_110_OE2	3	6	71	68	0	0	0	10
a_121_NH2__a_112_OG	0	0	0	0	0	7	3	1
a_121_NH2__a_119_O	0	0	0	0	0	1	0	0
a_121_NH2__a_119_OH	9	37	26	16	15	40	8	12
a_121_NH2__a_11_OE1	5	0	0	4	0	0	0	0
a_121_NH2__a_11_OE2	12	4	0	0	0	0	0	4
a_121_NH2__a_13_OD1	0	0	0	0	0	0	0	1
a_121_NH2__a_14_OH	25	11	42	9	1	0	0	2
a_121_NH2__a_16_OE2	0	0	0	0	0	0	0	2
**a_121_NH2__a_201_N**	**0**	**0**	**0**	**0**	**7**	**17**	**28**	**27**

**a_121_NH2__a_201_O2**	**0**	**0**	**0**	**0**	**24**	**58**	**85**	**49**
**a_121_NH2__a_201_O3**	**0**	**0**	**0**	**0**	**3**	**2**	**16**	**11**
**a_121_NH2__a_201_O4**	**0**	**0**	**0**	**0**	**42**	**29**	**47**	**28**
**a_121_NH2__a_201_O5**	**0**	**0**	**0**	**0**	**64**	**38**	**37**	**39**
**a_121_NH2__a_202_O7**	**0**	**0**	**0**	**0**	**1**	**4**	**60**	**13**
a_121_NH2__a_31_O	0	0	0	1	0	0	0	0
a_121_NH2__a_51_OE1	8	7	11	10	0	0	0	0
a_121_NH2__a_53_OH	0	0	1	4	0	0	0	0
a_121_NH2__a_74_SD	1	19	22	7	0	1	1	0
a_121_NH2__a_8_SD	26	8	0	3	2	6	0	3
a_121_NH2__a_97_OH	0	0	0	0	4	0	1	6
a_121_NH2__a_99_OE1	0	0	0	3	0	1	2	0
a_121_N__a_110_O	247	248	248	248	248	247	247	248
a_121_N__a_119_O	0	0	0	0	0	0	1	0
a_122_N__a_10_O	188	217	139	123	233	172	16	121
a_122_N__a_10_OG	10	0	4	5	0	1	0	1
a_122_N__a_120_O	0	0	0	0	0	0	13	0
a_122_N__a_8_SD	0	0	0	0	5	6	1	14
a_122_N__a_9_O	0	0	0	0	0	4	3	2
a_123_N__a_108_O	247	248	247	247	247	248	247	243
**a_123_N__a_201_O**	**0**	**0**	**0**	**0**	**0**	**0**	**0**	**1**
a_123_OG__a_108_O	22	27	59	21	12	13	29	35
a_123_OG__a_121_O	0	0	0	0	0	0	3	0
a_123_OG__a_122_O	2	2	1	6	6	2	2	3
a_123_OG__a_123_O	2	3	6	3	1	0	1	1
a_123_OG__a_124_O	0	1	0	0	9	4	8	5
**a_123_OG__a_201_O**	**0**	**0**	**0**	**0**	**0**	**0**	**0**	**2**
**a_123_OG__a_201_O1**	**0**	**0**	**0**	**0**	**0**	**6**	**0**	**9**
a_123_OG__a_7_O	14	5	4	10	199	145	169	136
a_123_OG__a_8_SD	2	4	1	5	0	0	0	1
a_124_NZ__a_106_OD1	3	3	26	14	8	13	17	10
a_124_NZ__a_106_OD2	2	10	22	5	3	17	25	12
a_124_NZ__a_125_O	3	5	19	8	29	24	25	12
a_124_NZ__a_7_OE1	3	3	6	8	33	15	21	16
a_124_NZ__a_7_OE2	4	3	3	8	35	27	13	12
a_124_NZ__a_8_O	2	0	0	0	5	0	0	0
a_124_NZ__a_9_OE1	0	0	0	0	23	3	3	5
a_124_NZ__a_9_OE2	0	0	0	3	21	4	2	4
a_124_N__a_123_OG	0	1	1	0	107	78	65	69
a_124_N__a_7_O	248	248	248	248	239	239	246	213
a_125_NE__a_105_O	2	5	1	9	3	0	1	5
a_125_NE__a_106_O	0	0	0	0	34	1	14	12
a_125_NE__a_106_OD1	10	14	11	5	36	8	12	31
a_125_NE__a_106_OD2	20	7	12	12	50	18	15	27
a_125_NE__a_125_O	0	0	0	0	2	0	1	2
a_125_NE__a_126_O	7	6	12	2	1	0	4	2
a_125_NE__a_3_O	0	0	8	9	0	1	0	2
a_125_NE__a_5_O	0	1	1	0	0	0	1	0
a_125_NH1__a_105_O	9	10	35	31	4	4	15	13

Table S1 C

a_125_NH1__a_106_O	0	0	0	0	0	0	0	3
a_125_NH1__a_106_OD1	14	11	0	2	8	7	15	4
a_125_NH1__a_106_OD2	5	4	2	0	1	7	6	11
a_125_NH1__a_125_O	5	1	1	1	0	0	1	3
a_125_NH1__a_126_O	0	0	0	4	0	0	0	3
a_125_NH1__a_3_O	1	1	1	0	2	0	0	4
a_125_NH1__a_5_O	0	0	0	1	0	0	0	0
a_125_NH2__a_105_O	0	1	0	2	2	2	0	1
a_125_NH2__a_106_O	0	0	0	0	1	0	0	0
a_125_NH2__a_106_OD1	6	18	12	5	46	14	21	50
a_125_NH2__a_106_OD2	22	11	14	14	79	17	9	28
a_125_NH2__a_126_O	0	0	0	1	1	0	1	0
a_125_NH2__a_3_O	0	2	2	6	1	0	0	5
a_125_N__a_106_O	248	247	246	248	103	149	194	139
a_125_N__a_106_OD1	0	0	0	0	1	0	0	1
a_125_N__a_106_OD2	0	0	0	0	0	0	1	2
a_126_N__a_124_O	8	8	9	8	0	0	0	0
a_126_N__a_5_O	230	233	231	227	221	242	231	209
a_127_N__a_125_O	0	0	5	3	0	0	1	2
a_127_N__a_5_O	0	2	13	31	24	67	34	14
a_12_NZ__a_10_O	3	1	2	0	0	21	103	41
a_12_NZ__a_10_OG	49	35	31	30	22	40	52	36
a_12_NZ__a_118_O	0	0	0	12	0	0	0	0
a_12_NZ__a_118_OG1	1	0	0	0	0	0	0	0
a_12_NZ__a_11_O	2	1	1	1	0	0	1	0
a_12_NZ__a_120_O	8	5	14	12	0	0	1	2
a_12_NZ__a_120_OE1	70	56	53	58	62	52	64	56
a_12_NZ__a_120_OE2	52	70	77	47	58	74	46	62
a_12_NZ__a_13_OD1	0	0	0	5	1	1	0	1
a_12_NZ__a_16_OE1	0	0	0	3	0	0	0	2
a_12_NZ__a_16_OE2	0	0	0	3	0	0	0	0
a_12_NZ__a_9_OE1	1	0	1	1	0	0	0	1
a_12_NZ__a_9_OE2	0	0	1	0	0	0	0	0
a_12_N__a_10_O	0	0	0	0	0	0	2	3
a_12_N__a_119_OH	50	25	58	37	0	0	1	0
a_12_N__a_11_OE1	2	0	13	51	0	0	0	5
a_12_N__a_11_OE2	2	7	46	50	0	0	0	6
a_12_N__a_120_O	178	202	106	82	232	201	149	182
**a_12_N__a_201_O4**	**0**	**0**	**0**	**0**	**0**	**0**	**15**	**0**
**a_12_N__a_201_O5**	**0**	**0**	**0**	**0**	**0**	**0**	**7**	**0**
a_13_ND2__a_118_O	90	28	27	80	179	147	135	144
a_13_ND2__a_118_OG1	0	0	0	0	0	1	0	0
a_13_ND2__a_119_OH	0	0	0	0	0	0	2	1
a_13_ND2__a_120_O	0	0	0	0	0	0	0	1
a_13_ND2__a_12_O	0	0	1	0	1	1	0	2
a_13_ND2__a_13_O	16	5	18	21	37	28	38	29
a_13_ND2__a_15_OD1	1	3	17	14	0	0	0	0
a_13_ND2__a_15_OD2	1	3	7	15	0	0	0	0
a_13_ND2__a_16_O	1	0	0	0	0	0	0	0

a_13_ND2__a_16_OE1	15	18	19	23	1	7	0	12
a_13_ND2__a_16_OE2	12	10	16	11	3	9	8	8
a_13_ND2__a_8_SD	0	0	0	0	0	0	0	2
a_13_N__a_119_OH	6	0	9	62	0	0	0	0
a_13_N__a_11_O	0	0	0	0	0	1	0	3
a_13_N__a_11_OE1	0	0	6	11	1	4	0	1
a_13_N__a_11_OE2	0	0	13	11	0	9	0	1
a_13_N__a_13_OD1	31	22	16	14	0	0	0	0
a_14_N__a_119_OH	0	0	0	1	0	0	0	0
a_14_N__a_11_O	0	0	0	0	0	0	1	1
a_14_N__a_11_OE1	0	0	26	65	1	1	0	1
a_14_N__a_11_OE2	0	0	16	49	0	0	0	2
a_14_N__a_12_O	158	85	89	33	1	1	5	4
a_14_N__a_13_OD1	0	1	0	0	0	3	0	0
a_14_N__a_15_OD1	1	21	4	0	0	0	0	0
a_14_N__a_15_OD2	0	4	0	0	0	0	0	0
**a_14_N__a_201_O4**	**0**	**0**	**0**	**0**	**0**	**0**	**1**	**0**
a_14_OH__a_112_OG	0	0	1	1	0	0	0	0
a_14_OH__a_119_OH	0	0	0	1	0	0	0	0
a_14_OH__a_11_OE1	0	1	0	0	15	4	4	7
a_14_OH__a_11_OE2	0	0	1	0	0	4	9	3
a_14_OH__a_15_OD2	0	0	0	0	0	8	0	0
a_14_OH__a_18_SD	0	4	1	0	0	0	0	0
**a_14_OH__a_201_N**	**0**	**0**	**0**	**0**	**1**	**0**	**0**	**0**
**a_14_OH__a_201_O4**	**0**	**0**	**0**	**0**	**23**	**10**	**0**	**2**
**a_14_OH__a_201_O5**	**0**	**0**	**0**	**0**	**26**	**16**	**0**	**4**
a_14_OH__a_24_O	0	0	0	0	1	0	0	0
a_14_OH__a_27_O	0	2	3	0	0	0	0	0
a_14_OH__a_28_O	135	12	34	23	2	3	28	25
a_14_OH__a_29_OE1	0	0	0	5	2	2	0	1
a_14_OH__a_29_OE2	0	1	0	4	1	2	0	0
a_14_OH__a_53_OH	0	0	0	1	0	0	0	0
a_14_OH__a_73_OG1	0	0	1	0	0	0	0	0
a_14_OH__a_74_O	0	1	2	1	0	0	0	0
a_14_OH__a_74_SD	1	45	28	23	0	0	0	0
a_14_OH__a_97_OH	0	1	0	0	0	0	0	0
a_15_N__a_12_O	0	0	0	0	0	0	0	4
a_15_N__a_13_O	0	0	0	2	0	0	0	4
a_15_N__a_13_OD1	8	26	16	14	2	3	0	0
a_15_N__a_15_OD1	34	46	57	48	24	19	27	14
a_15_N__a_15_OD2	34	45	10	23	5	25	17	24
a_15_N__a_16_OE1	0	2	0	0	0	0	0	0
a_16_N__a_13_O	22	0	25	110	31	31	67	59
a_16_N__a_13_OD1	37	81	71	20	3	19	0	0
a_16_N__a_14_O	0	0	0	0	1	0	0	0
a_16_N__a_15_OD1	12	9	10	15	1	7	13	4
a_16_N__a_15_OD2	10	7	4	7	1	5	7	10
a_16_N__a_16_O	0	0	0	0	0	0	0	6
a_16_N__a_16_OE1	0	14	1	6	0	8	6	0

Table S1 C

a_16_N__a_16_OE2	5	1	4	2	1	2	2	1
a_17_N__a_13_O	31	5	7	70	58	66	114	101
a_17_N__a_13_OD1	0	0	6	0	0	1	0	0
a_17_N__a_14_O	208	167	202	206	146	133	130	119
a_17_N__a_15_O	1	0	0	0	0	0	0	1
a_18_N__a_14_O	247	236	236	243	231	220	232	195
a_18_N__a_15_O	14	14	38	21	6	31	24	22
a_18_N__a_16_O	4	11	9	3	18	26	20	23
a_18_N__a_18_SD	0	0	0	0	1	0	0	0
a_19_NZ__a_15_O	10	5	1	0	9	1	11	6
a_19_NZ__a_15_OD1	1	7	4	7	4	11	22	25
a_19_NZ__a_15_OD2	1	17	7	2	3	15	35	26
a_19_NZ__a_16_O	0	0	1	0	0	2	0	2
a_19_NZ__a_16_OE1	98	89	80	70	107	87	90	66
a_19_NZ__a_16_OE2	83	89	96	89	104	108	91	81
a_19_NZ__a_24_O	0	0	0	0	0	0	4	1
a_19_N__a_15_O	207	212	220	219	107	155	170	141
a_19_N__a_16_O	44	131	88	53	150	166	154	133
a_19_N__a_17_O	208	128	145	163	187	176	162	159
a_19_N__a_18_SD	0	0	0	0	3	0	0	0
a_19_N__a_23_O	0	0	0	0	0	0	0	1
**a_201_O__a_101_O**	**0**	**0**	**0**	**0**	**7**	**0**	**0**	**4**
**a_201_O__a_101_OG**	**0**	**0**	**0**	**0**	**34**	**39**	**58**	**40**
**a_201_O__a_102_O**	**0**	**0**	**0**	**0**	**0**	**1**	**0**	**0**
**a_201_O__a_108_O**	**0**	**0**	**0**	**0**	**0**	**0**	**0**	**2**
**a_201_O__a_109_O**	**0**	**0**	**0**	**0**	**0**	**1**	**0**	**2**
**a_201_O__a_110_OE1**	**0**	**0**	**0**	**0**	**18**	**19**	**1**	**36**
**a_201_O__a_110_OE2**	**0**	**0**	**0**	**0**	**22**	**101**	**26**	**31**
**a_201_O__a_201_O1**	**0**	**0**	**0**	**0**	**0**	**2**	**7**	**0**
**a_201_O__a_202_O3**	**0**	**0**	**0**	**0**	**46**	**17**	**24**	**26**
**a_201_O__a_2_O**	**0**	**0**	**0**	**0**	**1**	**0**	**9**	**0**
**a_201_O__a_85_SD**	**0**	**0**	**0**	**0**	**1**	**0**	**0**	**0**
**a_201_O__a_88_O**	**0**	**0**	**0**	**0**	**10**	**6**	**1**	**26**
**a_201_O__a_90_O**	**0**	**0**	**0**	**0**	**1**	**0**	**0**	**0**
**a_201_O__a_99_OE1**	**0**	**0**	**0**	**0**	**17**	**0**	**2**	**0**
a_20_N__a_16_O	25	139	67	26	68	88	79	85
a_20_N__a_17_O	184	169	166	199	220	207	214	204
a_20_N__a_18_O	6	1	1	0	1	1	0	0
a_21_N__a_17_O	30	121	67	64	41	68	42	72
a_21_N__a_18_O	77	147	164	151	202	181	208	173
a_21_N__a_19_O	0	0	0	0	0	0	1	0
a_22_N__a_18_O	46	199	150	142	183	183	203	207
a_22_N__a_19_O	12	111	121	89	127	126	111	72
a_22_N__a_20_O	0	0	1	1	0	0	0	0
a_23_N__a_18_O	46	214	154	142	167	151	222	177
a_23_N__a_19_O	0	1	4	1	0	0	0	0
**a_23_N__a_202_OT2**	**0**	**0**	**0**	**0**	**0**	**1**	**0**	**0**
a_23_N__a_21_O	0	0	1	0	0	2	1	0
a_24_N__a_22_O	0	0	0	0	4	6	2	4

Table S1 C

a_24_N__a_74_SD	0	0	0	0	0	0	0	2
a_24_OG__a_15_O	0	0	0	0	0	0	1	0
a_24_OG__a_22_O	0	0	0	0	11	20	5	11
a_24_OG__a_23_O	0	0	2	0	2	0	1	10
a_24_OG__a_24_O	4	1	7	2	2	4	2	6
a_24_OG__a_26_OD1	15	15	33	11	0	0	0	1
a_24_OG__a_26_OD2	5	10	10	12	0	0	0	0
a_24_OG__a_75_O	0	4	0	0	0	0	0	0
a_25_N__a_15_OD2	0	0	0	0	0	0	1	0
**a_25_N__a_201_O4**	**0**	**0**	**0**	**0**	**0**	**0**	**0**	**1**
a_25_N__a_23_O	0	2	0	0	0	0	0	0
a_25_N__a_24_OG	2	29	3	21	3	5	15	4
a_25_N__a_74_SD	0	0	0	0	0	0	0	1
a_25_OG__a_14_OH	0	0	0	2	0	0	0	2
a_25_OG__a_15_OD1	0	0	5	7	0	0	0	0
a_25_OG__a_15_OD2	0	1	4	4	0	0	3	0
a_25_OG__a_18_SD	0	1	0	0	0	1	1	3
**a_25_OG__a_201_O4**	**0**	**0**	**0**	**0**	**0**	**0**	**0**	**5**
**a_25_OG__a_201_O5**	**0**	**0**	**0**	**0**	**0**	**0**	**0**	**3**
a_25_OG__a_23_O	0	2	0	0	0	3	0	0
a_25_OG__a_24_O	0	0	0	0	1	26	1	5
a_25_OG__a_24_OG	0	0	0	0	7	5	9	1
a_25_OG__a_25_O	1	0	8	0	1	4	3	3
a_25_OG__a_26_OD1	0	0	0	0	0	0	0	2
a_25_OG__a_29_OE1	0	0	11	1	3	8	2	12
a_25_OG__a_29_OE2	0	0	7	0	3	7	4	7
a_25_OG__a_74_O	0	0	0	0	0	0	0	1
a_25_OG__a_74_SD	0	0	0	0	0	0	0	3
a_26_N__a_24_O	17	26	27	26	0	0	22	4
a_26_N__a_24_OG	19	36	15	31	1	6	9	3
a_26_N__a_25_OG	9	11	14	12	45	45	15	45
a_26_N__a_26_OD1	5	5	15	11	1	0	0	14
a_26_N__a_26_OD2	2	8	5	10	1	4	1	9
a_26_N__a_29_OE1	0	0	11	0	0	0	0	2
a_26_N__a_29_OE2	0	0	19	0	0	0	0	3
a_27_N__a_24_O	215	163	158	186	0	0	9	14
a_27_N__a_24_OG	0	0	0	1	0	0	0	0
a_27_N__a_25_O	0	0	22	1	0	3	18	51
a_27_N__a_25_OG	0	0	0	0	0	1	0	11
a_27_N__a_26_OD1	1	0	4	2	2	6	1	12
a_27_N__a_26_OD2	0	1	3	2	2	6	2	14
a_28_N__a_24_O	118	47	64	91	0	0	1	9
a_28_N__a_25_O	75	147	112	108	182	143	86	82
a_28_N__a_25_OG	0	0	0	0	0	0	0	9
a_28_N__a_26_O	0	0	0	0	1	0	0	1
a_28_N__a_29_OE2	0	0	1	0	0	0	0	0
a_29_N__a_25_O	209	211	193	216	220	224	195	72
a_29_N__a_26_O	35	28	40	29	28	45	60	171
a_29_N__a_27_O	0	0	0	0	0	0	0	1

Table S1 C

a_29_N__a_29_OE1	0	0	7	0	4	0	0	0
a_29_N__a_29_OE2	0	0	16	0	5	0	0	1
a_2_N__a_41_OE1	0	0	0	0	0	0	1	0
a_2_N__a_42_O	0	0	0	1	43	52	34	39
a_2_N__a_42_OE1	0	0	0	24	119	185	127	131
a_2_N__a_85_SD	0	0	0	1	0	0	0	0
a_2_N__a_87_O	0	0	1	0	0	0	0	0
a_2_N__a_88_O	0	0	0	0	2	0	3	10
**a_30_NZ__a_201_O4**	**0**	**0**	**0**	**0**	**2**	**0**	**0**	**0**
**a_30_NZ__a_201_O5**	**0**	**0**	**0**	**0**	**4**	**0**	**0**	**0**
a_30_NZ__a_25_O	0	0	0	1	1	0	0	0
a_30_NZ__a_26_O	39	53	51	46	41	51	47	19
a_30_NZ__a_26_OD1	91	80	93	69	61	62	72	97
a_30_NZ__a_26_OD2	103	119	97	83	73	63	41	85
a_30_NZ__a_27_O	0	0	0	1	0	0	0	0
a_30_NZ__a_29_OE1	0	0	0	1	9	13	8	9
a_30_NZ__a_29_OE2	0	0	0	1	2	2	0	0
a_30_NZ__a_33_OD1	1	0	0	1	0	0	0	0
a_30_NZ__a_53_OH	0	0	0	0	14	0	2	27
a_30_NZ__a_54_O	0	0	0	1	0	0	2	2
a_30_NZ__a_54_OG	0	0	0	0	0	0	3	0
a_30_NZ__a_56_O	0	0	0	2	0	0	0	0
a_30_NZ__a_57_ND1	0	1	0	0	0	0	0	0
a_30_NZ__a_75_O	0	0	3	0	0	0	0	0
a_30_N__a_26_O	153	130	153	104	106	90	69	180
a_30_N__a_27_O	71	65	67	109	74	119	145	86
a_30_N__a_28_O	0	8	0	1	0	0	0	0
a_31_N__a_27_O	214	159	169	146	211	226	222	198
a_31_N__a_28_O	70	142	137	149	57	60	42	36
a_31_N__a_29_O	0	0	1	1	0	0	0	0
a_32_NE__a_11_OE1	33	45	94	68	16	22	8	12
a_32_NE__a_11_OE2	25	44	47	58	35	11	18	3
a_32_NE__a_12_O	0	0	0	1	0	0	0	0
a_32_NE__a_13_OD1	0	0	0	1	0	0	0	0
a_32_NE__a_14_OH	1	1	0	2	4	3	14	8
a_32_NE__a_15_OD1	4	0	0	1	0	0	0	0
a_32_NE__a_15_OD2	2	0	0	9	0	0	0	0
a_32_NE__a_28_O	0	0	0	0	2	0	1	10
a_32_NE__a_29_OE1	0	4	0	8	32	23	36	32
a_32_NE__a_29_OE2	0	6	0	18	30	46	30	36
a_32_NE__a_33_OD1	0	0	0	0	0	0	3	0
a_32_NH1__a_11_O	0	2	0	0	0	0	1	0
a_32_NH1__a_11_OE1	0	12	1	0	35	44	6	10
a_32_NH1__a_11_OE2	0	4	2	0	36	14	9	29
a_32_NH1__a_12_O	0	4	4	0	0	0	0	0
a_32_NH1__a_13_OD1	0	0	1	5	0	0	0	0
a_32_NH1__a_14_OH	0	0	0	0	10	6	23	22
a_32_NH1__a_15_OD1	21	56	12	18	20	37	12	27
a_32_NH1__a_15_OD2	27	52	44	44	29	4	12	25

Table S1 C

a_32_NH1__a_16_OE2	0	0	0	1	0	0	0	0
a_32_NH1__a_18_SD	0	0	0	0	0	3	0	0
**a_32_NH1__a_201_O4**	**0**	**0**	**0**	**0**	**1**	**0**	**0**	**0**
**a_32_NH1__a_201_O5**	**0**	**0**	**0**	**0**	**6**	**0**	**0**	**0**
a_32_NH1__a_28_O	0	0	0	1	4	0	2	2
a_32_NH1__a_29_OE1	13	22	11	23	6	7	11	17
a_32_NH1__a_29_OE2	29	14	13	26	5	6	34	29
a_32_NH1__a_32_O	0	0	1	0	0	0	0	0
a_32_NH1__a_33_OD1	0	0	0	0	2	1	5	0
a_32_NH2__a_119_OH	0	0	4	0	0	0	0	0
a_32_NH2__a_11_O	1	0	0	2	2	0	12	6
a_32_NH2__a_11_OE1	65	69	114	81	42	21	9	8
a_32_NH2__a_11_OE2	44	34	49	42	37	36	13	12
a_32_NH2__a_12_O	3	29	60	35	0	0	0	0
a_32_NH2__a_13_OD1	0	0	0	10	0	0	0	0
a_32_NH2__a_14_O	0	0	0	0	0	2	0	0
a_32_NH2__a_14_OH	0	0	0	1	4	6	4	7
a_32_NH2__a_15_OD1	27	52	64	42	29	5	21	15
a_32_NH2__a_15_OD2	26	53	11	23	3	27	2	28
a_32_NH2__a_18_SD	0	0	0	0	0	2	0	1
a_32_NH2__a_25_OG	0	0	0	0	0	2	0	0
a_32_NH2__a_28_O	0	0	0	0	1	0	0	9
a_32_NH2__a_29_OE1	9	4	5	28	56	57	97	56
a_32_NH2__a_29_OE2	2	8	3	13	55	46	64	45
a_32_NH2__a_33_OD1	0	0	0	0	0	0	1	1
a_32_N__a_14_OH	0	0	0	0	1	0	0	0
a_32_N__a_28_O	70	151	117	112	188	211	147	84
a_32_N__a_29_O	168	131	152	104	52	49	88	148
a_32_N__a_30_O	8	9	8	13	0	0	1	1
a_33_ND2__a_11_OE1	0	0	0	1	0	0	0	0
a_33_ND2__a_29_O	3	4	3	15	47	51	30	28
a_33_ND2__a_29_OE1	1	0	1	2	10	6	11	7
a_33_ND2__a_29_OE2	0	0	1	2	11	10	5	5
a_33_ND2__a_30_O	2	10	0	1	0	3	0	0
a_33_ND2__a_32_O	0	0	0	0	0	0	0	1
a_33_ND2__a_33_O	11	7	5	5	3	3	3	4
a_33_ND2__a_53_O	0	10	1	0	0	0	3	0
a_33_ND2__a_53_OH	0	1	0	0	0	0	0	0
a_33_ND2__a_54_O	0	11	4	16	0	0	4	0
a_33_ND2__a_54_OG	4	12	5	11	0	1	8	26
a_33_N__a_11_OE1	0	0	0	1	0	0	0	0
a_33_N__a_29_O	46	24	49	52	182	111	160	104
a_33_N__a_30_O	201	132	108	98	89	125	71	143
a_33_N__a_31_O	1	6	10	9	1	11	1	7
a_33_N__a_33_OD1	1	33	0	0	0	2	24	6
a_33_N__a_34_O	0	0	0	0	0	0	0	1
a_33_N__a_53_O	0	0	2	0	0	0	0	0
a_33_N__a_54_OG	0	0	0	6	0	0	0	0
a_34_N__a_29_O	0	0	0	0	8	0	0	0

Table S1 C

a_34_N__a_30_O	71	31	47	69	219	184	205	210
a_34_N__a_31_O	24	41	14	22	16	55	20	7
a_34_N__a_32_O	0	0	2	10	0	0	0	0
a_34_N__a_33_OD1	0	2	0	6	0	0	10	0
a_34_N__a_53_O	0	5	12	0	0	0	0	0
a_34_N__a_54_OG	0	0	0	15	0	0	0	0
a_35_NZ__a_11_OE1	0	0	0	0	3	20	73	34
a_35_NZ__a_11_OE2	0	0	0	0	23	25	79	32
**a_35_NZ__a_201_O4**	**0**	**0**	**0**	**0**	**2**	**0**	**0**	**0**
**a_35_NZ__a_201_O5**	**0**	**0**	**0**	**0**	**2**	**0**	**0**	**0**
a_35_NZ__a_31_O	0	0	0	0	4	0	11	3
a_35_NZ__a_32_O	0	0	0	0	9	0	17	1
a_35_NZ__a_33_O	0	0	0	0	1	0	0	0
a_35_NZ__a_36_O	3	0	0	1	0	2	0	0
a_35_NZ__a_54_OG	1	0	0	0	0	0	0	0
a_35_NZ__a_7_OE1	68	37	67	60	76	63	26	33
a_35_NZ__a_7_OE2	76	54	76	107	77	63	16	36
a_35_NZ__a_8_O	24	32	26	20	61	74	45	87
a_35_NZ__a_8_SD	0	0	0	0	2	1	0	0
a_35_NZ__a_9_O	23	65	28	15	0	8	4	40
a_35_NZ__a_9_OE1	0	2	0	0	43	3	12	4
a_35_NZ__a_9_OE2	8	10	10	13	40	2	7	8
a_35_N__a_30_O	0	0	0	0	126	76	157	105
a_35_N__a_31_O	0	0	0	0	125	154	92	34
a_35_N__a_32_O	0	0	0	0	11	19	9	0
a_35_N__a_33_O	0	0	0	2	0	0	0	0
a_35_N__a_33_OD1	0	0	0	8	0	0	0	0
a_35_N__a_53_O	0	0	0	9	0	0	0	0
a_35_N__a_54_OG	0	0	0	16	0	0	0	0
a_35_N__a_8_SD	0	0	0	1	0	0	0	0
**a_36_N__a_201_O4**	**0**	**0**	**0**	**0**	**1**	**2**	**0**	**4**
**a_36_N__a_201_O5**	**0**	**0**	**0**	**0**	**0**	**2**	**0**	**2**
a_36_N__a_31_O	0	0	0	0	4	0	0	0
a_36_N__a_34_O	9	11	28	17	0	0	0	0
a_36_N__a_8_SD	70	92	87	124	26	32	63	50
a_37_N__a_35_O	0	0	2	0	0	4	0	1
a_37_N__a_51_OE1	0	0	0	0	0	0	0	1
a_37_N__a_52_O	242	232	218	211	212	215	236	218
a_38_N__a_6_O	248	247	248	247	206	229	237	229
a_38_OG1__a_123_OG	0	1	1	1	0	0	0	0
**a_38_OG1__a_201_O4**	**0**	**0**	**0**	**0**	**4**	**0**	**0**	**4**
a_38_OG1__a_36_O	1	0	3	0	0	0	0	1
a_38_OG1__a_37_O	1	5	7	28	13	7	51	33
a_38_OG1__a_38_O	2	7	13	8	2	3	0	3
a_38_OG1__a_39_O	0	0	0	0	2	6	0	2
a_38_OG1__a_50_O	0	27	39	35	139	165	179	112
a_38_OG1__a_51_OE1	138	23	60	11	13	7	2	4
a_38_OG1__a_6_O	3	20	26	24	8	27	2	29
a_38_OG1__a_8_SD	0	0	1	0	0	0	0	0

Table S1 C

a_39_N__a_37_O	0	0	1	0	0	0	0	0
a_39_N__a_38_OG1	0	8	18	6	68	66	83	50
a_39_N__a_39_OE1	0	0	0	0	0	1	0	8
a_39_N__a_39_OE2	0	0	0	0	0	1	0	0
a_39_N__a_50_O	248	239	242	245	236	232	217	238
a_39_N__a_50_OG	0	2	0	2	2	4	4	1
a_3_N__a_1_O	30	0	0	45	83	82	70	91
a_3_OG1__a_1_O	2	0	0	3	2	14	0	16
a_3_OG1__a_2_O	12	13	9	11	6	0	0	0
a_3_OG1__a_3_O	17	15	23	20	34	42	27	33
a_3_OG1__a_40_O	24	9	43	18	0	4	19	3
a_3_OG1__a_41_OE1	12	18	15	6	16	13	8	3
a_3_OG1__a_42_O	0	0	0	0	49	23	7	11
a_3_OG1__a_42_OE1	1	1	4	1	0	0	1	2
a_40_N__a_4_O	248	247	245	242	248	246	242	247
a_41_NE2__a_39_OE1	2	2	5	14	1	3	5	3
a_41_NE2__a_39_OE2	5	6	2	11	3	1	5	3
a_41_NE2__a_3_OG1	0	0	0	0	6	0	0	0
a_41_NE2__a_40_O	5	4	12	12	8	19	5	18
a_41_NE2__a_41_O	0	0	1	2	1	3	0	1
a_41_NE2__a_42_O	8	13	11	6	5	0	3	1
a_41_NE2__a_42_OE1	2	0	0	0	0	0	0	0
a_41_NE2__a_43_OD1	1	0	2	2	0	1	0	2
a_41_NE2__a_43_OD2	1	0	1	0	0	1	0	1
a_41_NE2__a_48_O	9	9	10	6	1	7	10	2
a_41_NE2__a_48_OG1	15	28	7	21	3	2	4	4
a_41_NE2__a_50_OG	0	0	0	1	0	0	0	0
a_41_N__a_41_OE1	0	0	0	2	0	0	0	0
a_41_N__a_48_O	247	245	247	238	248	247	245	245
a_41_N__a_48_OG1	3	3	0	0	0	0	0	0
a_42_NE2__a_1_O	0	2	10	0	0	0	0	0
a_42_NE2__a_2_O	8	1	8	0	0	0	0	0
a_42_NE2__a_3_OG1	0	0	0	0	0	0	0	1
a_42_NE2__a_41_O	5	0	8	1	0	0	0	0
a_42_NE2__a_42_O	8	12	10	11	0	0	0	1
a_42_NE2__a_43_O	0	0	0	0	0	0	1	0
a_42_NE2__a_44_O	84	101	96	111	141	143	133	145
a_42_NE2__a_45_O	2	4	0	11	2	1	0	1
a_42_NE2__a_46_O	18	25	3	15	29	18	27	9
a_42_NE2__a_85_SD	0	0	1	0	4	16	1	5
a_42_N__a_2_O	2	2	34	20	28	82	40	54
a_42_N__a_3_OG1	9	9	15	11	75	35	17	38
a_42_N__a_40_O	0	0	0	2	0	0	0	0
a_42_N__a_41_OE1	54	43	49	23	54	30	37	29
a_42_N__a_42_OE1	7	7	11	1	0	1	0	0
a_43_N__a_41_O	0	0	0	1	0	0	0	0
a_43_N__a_44_O	0	0	0	0	0	0	1	1
a_43_N__a_46_O	237	244	242	213	128	105	132	135
a_44_N__a_42_O	5	0	1	1	0	0	1	1

a_44_N__a_42_OE1	6	6	7	1	0	0	1	2
a_44_N__a_43_OD1	0	0	0	0	2	1	1	2
a_44_N__a_43_OD2	0	0	0	0	1	0	2	0
a_44_N__a_46_O	0	0	0	16	0	0	0	7
a_45_NE2__a_43_OD2	0	0	0	0	0	0	0	3
a_45_NE2__a_44_O	1	1	4	2	1	1	1	5
a_45_NE2__a_45_O	1	5	3	4	3	1	2	1
a_45_NE2__a_46_OD1	0	0	2	0	0	0	0	1
a_45_NE2__a_46_OD2	0	0	1	0	0	0	2	0
a_45_NE2__a_64_OG1	0	0	0	0	0	0	0	1
a_45_NE2__a_65_O	74	56	81	40	128	127	111	76
a_45_NE2__a_85_SD	0	0	0	0	0	2	2	3
a_45_N__a_43_O	1	2	1	0	0	0	1	1
a_45_N__a_43_OD1	0	0	0	1	0	0	0	1
a_45_N__a_43_OD2	0	0	0	0	0	0	0	4
a_45_N__a_45_OE1	0	1	4	4	2	4	3	1
a_46_N__a_43_O	14	13	9	7	6	8	13	8
a_46_N__a_43_OD1	0	0	0	1	0	0	0	1
a_46_N__a_43_OD2	0	0	0	0	0	0	0	4
a_46_N__a_44_O	0	0	0	2	0	1	1	0
a_46_N__a_46_OD1	3	3	0	3	3	0	2	0
a_46_N__a_46_OD2	3	3	0	2	0	0	2	1
a_47_N__a_45_O	0	2	1	0	0	0	1	2
a_47_N__a_46_OD2	0	0	1	0	0	0	0	0
a_47_N__a_63_O	248	248	248	246	248	248	248	248
a_48_N__a_41_O	248	247	241	220	248	246	241	226
a_48_N__a_41_OE1	0	0	0	1	0	0	0	0
a_48_N__a_43_OD2	0	0	0	1	0	0	0	0
a_48_N__a_46_O	0	0	0	0	0	0	0	4
a_48_OG1__a_41_O	26	78	35	38	9	28	31	32
a_48_OG1__a_41_OE1	1	0	1	5	2	1	0	0
a_48_OG1__a_43_OD2	0	0	1	1	0	0	0	0
a_48_OG1__a_46_O	0	0	1	0	0	0	9	6
a_48_OG1__a_46_OD1	0	0	1	0	5	0	0	2
a_48_OG1__a_46_OD2	0	0	0	0	1	0	0	3
a_48_OG1__a_47_O	1	2	9	9	21	24	25	21
a_48_OG1__a_48_O	34	51	36	32	7	6	25	10
a_48_OG1__a_49_O	0	0	0	1	0	0	0	0
a_48_OG1__a_50_OG	0	0	1	0	0	0	0	0
a_48_OG1__a_60_OG1	0	0	0	3	0	0	0	0
a_48_OG1__a_61_O	0	0	12	12	29	52	23	45
a_49_NE1__a_110_OE1	0	0	1	1	0	0	0	0
a_49_NE1__a_110_OE2	0	1	0	0	0	0	0	0
**a_49_NE1__a_201_O**	**0**	**0**	**0**	**0**	**0**	**0**	**2**	**0**
**a_49_NE1__a_201_O2**	**0**	**0**	**0**	**0**	**0**	**15**	**31**	**23**
**a_49_NE1__a_201_O4**	**0**	**0**	**0**	**0**	**8**	**6**	**0**	**0**
**a_49_NE1__a_201_O5**	**0**	**0**	**0**	**0**	**7**	**3**	**0**	**0**
**a_49_NE1__a_202_O7**	**0**	**0**	**0**	**0**	**0**	**0**	**0**	**3**
a_49_NE1__a_38_OG1	5	19	30	35	17	5	40	20

Table S1 C

a_49_NE1__a_51_OE1	21	92	20	61	70	20	19	34
a_49_NE1__a_59_SD	0	0	1	0	1	0	0	0
a_49_NE1__a_61_OD1	0	0	0	0	19	30	33	20
a_49_NE1__a_73_O	0	0	1	0	0	0	0	0
a_49_NE1__a_74_SD	0	0	0	0	11	1	0	1
a_49_N__a_48_OG1	0	0	8	7	19	25	7	17
a_49_N__a_60_OG1	0	0	0	1	0	0	0	0
a_49_N__a_61_O	248	248	237	245	245	245	246	247
a_49_N__a_61_OD1	0	0	0	0	0	2	0	0
a_4_N__a_3_OG1	14	7	24	10	0	2	10	3
a_4_N__a_40_O	241	233	216	234	240	201	215	220
a_4_N__a_41_OE1	2	1	1	5	0	1	1	1
a_50_N__a_38_OG1	0	0	0	0	1	6	6	2
a_50_N__a_39_O	247	246	243	246	236	237	230	243
a_50_N__a_41_OE1	0	1	0	0	0	0	0	0
a_50_OG__a_39_O	26	26	64	67	40	33	39	44
a_50_OG__a_39_OE1	0	0	0	1	1	0	0	0
a_50_OG__a_39_OE2	0	0	0	1	0	0	0	0
a_50_OG__a_41_OE1	4	0	3	6	0	0	1	10
a_50_OG__a_48_OG1	0	0	2	0	0	0	0	0
a_50_OG__a_49_O	0	10	1	0	1	10	0	11
a_50_OG__a_50_O	6	2	12	1	1	3	3	4
a_50_OG__a_52_NE2	0	0	1	0	4	3	3	9
a_50_OG__a_58_O	0	0	0	0	1	0	0	0
a_50_OG__a_58_OG1	0	7	2	0	6	5	6	1
a_50_OG__a_59_O	3	14	7	9	26	61	35	21
a_50_OG__a_60_OG1	126	119	57	32	22	14	2	1
a_51_NE2__a_110_OE1	0	0	2	0	0	0	0	0
**a_51_NE2__a_201_N**	**0**	**0**	**0**	**0**	**7**	**4**	**9**	**10**
**a_51_NE2__a_201_O2**	**0**	**0**	**0**	**0**	**74**	**37**	**131**	**65**
**a_51_NE2__a_201_O4**	**0**	**0**	**0**	**0**	**6**	**0**	**0**	**2**
**a_51_NE2__a_201_O5**	**0**	**0**	**0**	**0**	**5**	**1**	**0**	**3**
a_51_NE2__a_37_O	7	25	8	3	0	0	1	0
a_51_NE2__a_38_OG1	3	14	48	54	16	9	1	18
a_51_NE2__a_50_O	6	8	0	0	0	1	0	0
a_51_NE2__a_51_O	0	0	0	0	6	5	1	9
a_51_NE2__a_52_O	0	0	0	0	0	1	0	3
a_51_NE2__a_53_OH	12	68	5	48	1	0	0	11
a_51_NE2__a_59_O	3	8	2	4	0	2	0	0
a_51_NE2__a_59_SD	0	8	1	4	1	9	0	1
a_51_NE2__a_72_OE1	0	2	0	0	0	0	0	0
a_51_NE2__a_73_O	5	0	6	0	0	0	0	0
a_51_NE2__a_74_SD	0	0	2	0	1	4	0	1
a_51_N__a_50_OG	0	9	1	1	5	1	0	0
a_51_N__a_51_OE1	1	0	1	6	0	21	29	28
a_51_N__a_58_OG1	1	4	7	2	0	1	0	1
a_51_N__a_59_O	247	247	248	246	245	224	233	240
a_52_ND1__a_37_O	0	0	0	0	4	9	8	10
a_52_ND1__a_39_OE1	0	0	0	0	8	8	8	8

Table S1 C

a_52_ND1__a_50_O	0	0	0	0	5	4	12	3
a_52_ND1__a_50_OG	0	0	0	0	32	8	34	6
a_52_ND1__a_51_O	0	0	0	0	0	2	0	0
a_52_ND1__a_52_O	0	0	0	0	8	6	4	6
a_52_ND1__a_53_O	0	0	0	0	0	0	0	3
a_52_ND1__a_54_OG	0	0	0	0	0	0	2	0
a_52_ND1__a_57_O	0	0	0	0	0	4	1	27
a_52_ND1__a_58_OG1	0	0	0	0	24	23	9	50
a_52_NE2__a_39_OE1	62	12	36	10	0	0	0	0
a_52_NE2__a_39_OE2	44	8	24	5	0	0	0	0
a_52_NE2__a_50_OG	2	0	0	0	0	0	0	0
a_52_NE2__a_53_O	0	0	0	1	0	0	0	0
a_52_NE2__a_54_O	0	0	1	2	0	0	0	0
a_52_NE2__a_55_O	4	1	2	7	0	0	0	0
a_52_NE2__a_56_O	0	0	0	2	0	0	0	0
a_52_NE2__a_58_OG1	0	1	0	0	0	0	0	0
a_52_N__a_37_O	248	247	245	244	216	223	241	240
a_52_N__a_50_O	0	0	0	0	0	0	3	0
a_52_N__a_51_OE1	0	0	0	0	0	0	0	1
a_52_N__a_52_ND1	0	0	0	0	1	0	2	0
a_53_N__a_51_O	8	2	3	11	0	0	0	0
a_53_N__a_52_ND1	1	7	7	2	0	0	0	4
a_53_N__a_54_O	0	0	0	1	0	0	0	0
a_53_N__a_57_ND1	0	0	0	0	1	0	0	0
a_53_N__a_57_O	136	68	133	192	195	155	146	138
a_53_N__a_58_OG1	2	2	1	2	0	0	1	1
a_53_OH__a_14_OH	0	0	1	11	0	0	0	0
**a_53_OH__a_201_O2**	**0**	**0**	**0**	**0**	**0**	**0**	**0**	**3**
**a_53_OH__a_201_O4**	**0**	**0**	**0**	**0**	**1**	**0**	**0**	**8**
**a_53_OH__a_201_O5**	**0**	**0**	**0**	**0**	**0**	**0**	**0**	**8**
a_53_OH__a_26_OD1	0	0	0	0	0	0	3	7
a_53_OH__a_26_OD2	0	0	0	0	0	0	2	10
a_53_OH__a_27_O	0	0	3	2	0	1	0	1
a_53_OH__a_30_O	0	3	0	0	0	0	0	0
a_53_OH__a_31_O	0	0	1	0	0	0	0	0
a_53_OH__a_51_OE1	34	29	96	63	7	0	0	9
a_53_OH__a_57_ND1	4	11	11	0	9	8	10	2
a_53_OH__a_57_NE2	0	1	1	0	0	0	0	0
a_53_OH__a_58_O	0	0	2	0	0	0	0	0
a_53_OH__a_59_SD	4	7	12	3	0	0	0	0
a_53_OH__a_73_O	2	0	0	27	0	0	0	0
a_53_OH__a_74_O	0	0	0	3	0	0	0	0
a_53_OH__a_74_SD	9	0	4	4	0	0	1	0
a_53_OH__a_75_O	1	0	2	1	0	0	0	0
a_54_N__a_30_O	0	0	0	2	0	0	0	0
a_54_N__a_33_OD1	0	0	0	9	0	0	0	0
a_54_N__a_34_O	0	0	0	0	8	2	1	0
a_54_N__a_35_O	0	0	0	24	0	1	0	0
a_54_N__a_52_ND1	3	8	27	4	0	0	0	0

a_54_N__a_52_O	0	0	1	2	0	0	0	0
a_54_N__a_57_O	98	24	62	49	0	0	0	0
a_54_OG__a_30_O	0	2	0	49	1	7	0	4
a_54_OG__a_31_O	0	1	0	1	0	0	0	0
a_54_OG__a_33_OD1	0	5	1	14	0	1	0	2
a_54_OG__a_34_O	0	0	0	9	21	15	6	0
a_54_OG__a_35_O	0	2	10	13	0	1	0	0
a_54_OG__a_53_O	16	6	8	5	0	0	3	0
a_54_OG__a_54_O	5	8	15	43	4	6	11	11
a_54_OG__a_55_O	0	0	0	1	0	0	0	0
a_54_OG__a_57_ND1	0	0	0	1	0	0	0	0
a_54_OG__a_57_O	4	7	11	1	0	0	0	0
a_55_N__a_52_ND1	1	2	5	2	0	0	0	0
a_55_N__a_53_O	0	1	0	8	234	233	118	21
a_55_N__a_54_OG	39	24	51	17	0	0	2	2
a_55_N__a_57_O	46	9	37	13	0	0	0	0
a_55_N__a_58_OG1	4	0	3	0	0	0	0	0
a_56_N__a_53_O	0	0	0	82	137	133	95	126
a_56_N__a_54_O	2	2	3	9	0	0	0	0
a_56_N__a_54_OG	142	80	117	28	0	0	0	0
a_56_N__a_57_O	0	1	0	0	0	0	0	0
a_57_NE2__a_14_OH	0	0	0	1	0	0	0	0
**a_57_NE2__a_201_O5**	**0**	**0**	**0**	**0**	**0**	**1**	**0**	**0**
a_57_NE2__a_27_O	0	0	1	1	0	0	0	0
a_57_NE2__a_30_O	1	0	1	0	0	0	0	0
a_57_NE2__a_51_OE1	0	0	0	0	25	25	41	20
a_57_NE2__a_53_OH	0	0	1	0	0	0	0	0
a_57_NE2__a_59_SD	2	1	0	2	27	18	21	15
a_57_NE2__a_73_O	0	0	0	11	0	0	0	0
a_57_NE2__a_74_O	0	0	0	41	0	0	0	1
a_57_NE2__a_74_SD	0	0	4	5	2	1	0	0
a_57_NE2__a_75_O	0	8	13	2	0	0	0	0
a_57_N__a_30_O	0	0	0	3	0	0	0	0
a_57_N__a_53_O	0	0	0	56	94	50	57	64
a_57_N__a_54_O	14	6	12	35	0	0	0	0
a_57_N__a_54_OG	131	68	106	16	0	0	0	0
a_57_N__a_55_O	0	0	1	0	1	0	2	4
a_58_N__a_56_O	16	7	15	18	0	0	0	2
a_58_N__a_57_ND1	20	28	19	30	24	34	36	21
a_58_N__a_58_OG1	98	53	110	133	62	52	59	56
a_58_OG1__a_50_OG	0	1	2	0	0	0	0	0
a_58_OG1__a_51_O	2	64	27	19	8	14	8	25
a_58_OG1__a_52_ND1	63	28	53	37	0	0	0	0
a_58_OG1__a_52_NE2	0	0	0	0	8	9	8	1
a_58_OG1__a_53_O	0	0	0	0	0	0	1	0
a_58_OG1__a_54_O	0	3	0	1	0	0	0	0
a_58_OG1__a_56_O	8	8	1	6	0	2	5	9
a_58_OG1__a_57_O	8	10	7	11	49	49	68	46
a_58_OG1__a_58_O	13	6	18	39	20	10	15	15

Table S1 C

a_58_OG1__a_59_O	0	1	3	0	0	0	0	0
a_58_OG1__a_60_OG1	0	0	1	0	0	0	0	0
a_59_N__a_51_O	239	212	227	239	242	246	244	243
a_59_N__a_57_O	1	0	0	3	0	0	0	1
a_59_N__a_58_OG1	3	85	43	32	35	46	34	36
a_5_NZ__a_126_O	0	0	1	0	0	0	0	0
a_5_NZ__a_38_O	0	0	0	0	0	1	0	0
a_5_NZ__a_39_OE1	11	20	24	21	19	33	18	12
a_5_NZ__a_39_OE2	30	20	33	40	22	14	14	6
a_5_NZ__a_7_OE1	0	0	0	0	2	0	2	10
a_5_NZ__a_7_OE2	0	0	0	0	8	1	14	14
a_5_N__a_126_O	4	4	3	5	1	15	11	16
a_60_N__a_58_O	16	8	19	6	14	2	8	3
a_60_N__a_59_SD	11	9	4	16	17	5	14	14
a_60_N__a_60_O	243	247	240	247	235	239	216	237
a_60_N__a_60_OG1	45	52	67	60	190	191	215	166
a_60_N__a_72_OE1	10	24	14	28	0	0	0	0
a_60_N__a_74_O	0	0	0	0	0	5	9	2
a_60_OG1__a_48_OG1	0	0	0	2	0	0	0	0
a_60_OG1__a_49_O	9	9	23	38	0	4	0	26
a_60_OG1__a_50_OG	28	14	19	12	0	3	2	5
a_60_OG1__a_58_O	12	0	3	1	0	0	0	0
a_60_OG1__a_58_OG1	3	7	6	0	0	0	0	0
a_60_OG1__a_59_O	12	38	20	9	1	2	1	1
a_60_OG1__a_60_O	18	20	23	25	17	16	34	15
a_60_OG1__a_61_O	0	0	0	1	0	0	0	0
**a_61_ND2__a_201_O4**	**0**	**0**	**0**	**0**	**0**	**3**	**0**	**2**
**a_61_ND2__a_201_O5**	**0**	**0**	**0**	**0**	**0**	**3**	**0**	**4**
**a_61_ND2__a_202_O3**	**0**	**0**	**0**	**0**	**18**	**0**	**0**	**0**
**a_61_ND2__a_202_O7**	**0**	**0**	**0**	**0**	**18**	**29**	**22**	**32**
a_61_ND2__a_49_O	55	19	36	7	4	5	6	0
a_61_ND2__a_51_OE1	12	8	11	52	0	7	23	6
a_61_ND2__a_59_O	2	0	1	1	1	0	3	0
a_61_ND2__a_59_SD	3	8	3	18	22	2	3	0
a_61_ND2__a_60_O	0	0	0	1	0	1	3	2
a_61_ND2__a_61_O	8	0	0	1	2	4	5	0
a_61_ND2__a_62_O	2	6	7	3	0	1	12	1
a_61_ND2__a_69_OG	0	2	0	7	0	0	0	0
a_61_ND2__a_70_O	18	64	40	39	0	0	0	8
a_61_ND2__a_70_OD1	0	0	0	1	0	0	0	0
a_61_ND2__a_72_O	0	0	0	0	33	15	16	17
a_61_ND2__a_72_OE1	12	4	6	14	2	23	6	5
a_61_ND2__a_73_O	66	17	128	86	16	1	0	3
a_61_ND2__a_74_O	0	0	0	0	0	1	0	0
a_61_ND2__a_74_SD	0	0	0	0	58	0	0	7
a_61_N__a_49_O	233	245	241	244	241	239	240	245
a_61_N__a_59_O	0	1	2	0	0	0	0	0
a_61_N__a_60_OG1	5	4	3	15	0	1	0	14
a_61_N__a_61_OD1	0	0	0	0	0	1	0	0

Table S1 C

a_62_NZ__a_43_O	2	14	0	31	0	0	0	1
a_62_NZ__a_43_OD1	32	54	21	52	7	3	3	11
a_62_NZ__a_43_OD2	41	48	31	51	3	7	2	14
a_62_NZ__a_46_OD1	16	17	29	16	14	14	25	27
a_62_NZ__a_46_OD2	9	10	34	24	13	14	26	23
a_62_NZ__a_47_O	0	0	1	1	0	0	0	0
a_62_NZ__a_48_OG1	0	8	31	5	21	8	10	23
a_62_NZ__a_60_OG1	0	0	0	0	8	8	0	8
a_62_NZ__a_63_O	0	0	0	0	0	0	1	0
a_62_NZ__a_64_OG1	1	0	0	0	0	3	2	4
a_62_NZ__a_70_OD1	0	0	0	0	0	0	0	1
a_62_NZ__a_72_OE1	0	0	0	0	1	0	0	0
a_62_N__a_60_O	1	1	2	0	0	0	0	0
a_62_N__a_61_OD1	10	11	9	11	60	75	58	94
a_62_N__a_70_O	0	0	0	3	0	0	0	0
a_62_N__a_72_O	0	0	0	0	0	0	0	3
a_62_N__a_72_OE1	0	0	0	1	39	26	37	28
a_63_N__a_47_O	248	248	247	246	240	244	243	247
a_63_N__a_61_O	0	0	0	1	0	0	0	0
a_64_N__a_46_OD1	0	0	0	1	0	0	0	0
a_64_N__a_46_OD2	0	0	0	1	0	0	0	0
a_64_N__a_69_OG	209	226	225	221	0	0	0	0
a_64_N__a_70_O	0	0	0	0	11	0	14	10
a_64_N__a_70_OD1	0	0	0	0	186	228	222	223
a_64_OG1__a_45_O	5	1	17	5	11	6	12	19
a_64_OG1__a_46_OD1	101	113	85	86	109	96	106	88
a_64_OG1__a_46_OD2	99	87	72	98	73	107	90	87
a_64_OG1__a_63_O	1	1	2	2	2	2	5	4
a_64_OG1__a_64_O	18	13	23	19	4	0	1	2
a_64_OG1__a_67_O	0	10	5	4	4	0	0	0
a_64_OG1__a_68_O	9	2	2	1	0	0	0	0
a_64_OG1__a_69_OG	7	7	3	12	0	0	0	0
a_64_OG1__a_70_OD1	0	0	0	0	25	13	16	17
a_65_N__a_45_O	157	130	196	124	237	246	208	203
a_65_N__a_63_O	0	0	0	0	1	0	0	1
a_65_N__a_64_OG1	2	0	4	16	1	2	0	7
a_66_N__a_64_O	2	4	4	4	0	0	1	2
a_66_N__a_82_OG1	0	0	0	0	7	0	0	0
a_66_N__a_83_O	234	211	214	170	213	202	219	178
a_67_NZ__a_45_OE1	0	0	0	0	3	0	0	2
a_67_NZ__a_64_OG1	16	7	2	10	18	19	11	3
a_67_NZ__a_65_O	0	0	0	0	3	1	0	0
a_67_NZ__a_67_O	0	0	0	1	0	0	0	0
a_67_NZ__a_68_O	21	15	11	8	2	1	0	1
a_67_NZ__a_68_OE1	25	8	7	13	4	3	1	0
a_67_NZ__a_68_OE2	20	6	2	8	3	0	1	2
a_67_N__a_64_O	64	61	86	38	50	61	67	61
a_67_N__a_65_O	0	1	0	6	0	0	0	3
a_67_N__a_83_O	23	12	21	22	1	0	3	0

a_68_N__a_68_OE1	82	29	53	86	14	12	19	51
a_68_N__a_68_OE2	84	46	48	78	13	13	15	45
a_68_N__a_81_O	0	0	0	2	0	0	0	0
a_69_N__a_67_O	1	9	2	2	0	0	0	0
a_69_N__a_68_OE1	0	0	0	0	0	0	3	3
a_69_N__a_68_OE2	0	0	0	0	0	0	2	0
a_69_N__a_79_O	0	0	0	0	20	34	14	74
a_69_N__a_80_O	0	0	0	0	0	2	0	0
a_69_N__a_81_O	247	245	246	243	157	95	171	37
a_69_N__a_82_OG1	0	0	0	0	0	0	2	1
a_69_OG__a_61_OD1	0	0	0	4	0	0	0	0
a_69_OG__a_62_O	6	1	3	3	0	0	0	0
a_69_OG__a_64_O	23	29	24	37	0	0	0	0
a_69_OG__a_64_OG1	1	8	2	3	0	0	0	0
a_69_OG__a_67_O	53	90	65	40	0	0	0	0
a_69_OG__a_68_O	85	68	70	39	2	6	11	29
a_69_OG__a_68_OE1	0	0	0	0	24	9	28	27
a_69_OG__a_68_OE2	0	0	0	0	49	11	19	41
a_69_OG__a_69_O	2	0	0	2	106	148	129	73
a_69_OG__a_70_O	0	5	1	2	0	0	0	0
a_69_OG__a_78_OG1	0	0	0	0	6	11	31	7
a_69_OG__a_79_O	0	0	0	0	19	12	5	0
a_69_OG__a_81_O	9	0	3	2	0	0	0	0
a_6_N__a_38_O	248	248	248	248	233	238	243	246
a_6_N__a_38_OG1	0	0	0	0	1	0	0	0
a_6_N__a_4_O	0	0	0	0	0	1	0	0
a_70_ND2__a_61_OD1	0	7	2	0	0	0	0	0
a_70_ND2__a_62_O	0	0	0	0	4	0	0	0
a_70_ND2__a_64_OG1	0	0	0	0	20	7	3	16
a_70_ND2__a_67_O	0	0	0	0	1	4	0	4
a_70_ND2__a_68_O	0	0	0	0	12	11	16	25
a_70_ND2__a_68_OE1	0	0	0	1	0	0	0	0
a_70_ND2__a_69_O	0	0	0	0	0	0	0	2
a_70_ND2__a_70_O	1	0	1	0	3	1	0	0
a_70_ND2__a_72_OE1	1	0	16	0	0	0	0	0
a_70_ND2__a_78_OG1	53	65	26	51	0	0	0	0
a_70_N__a_62_O	0	0	0	5	0	0	0	0
a_70_N__a_67_O	0	0	0	0	20	9	16	7
a_70_N__a_68_O	0	0	0	0	18	10	9	2
a_70_N__a_69_OG	5	4	7	5	0	0	0	0
a_70_N__a_70_OD1	0	1	2	0	2	0	0	0
a_70_N__a_79_O	0	0	0	0	0	0	0	1
a_70_N__a_81_O	0	0	0	0	17	2	13	21
a_71_N__a_67_O	0	0	0	0	0	1	0	0
a_71_N__a_69_O	3	1	3	11	5	0	9	2
a_71_N__a_70_OD1	0	2	5	0	0	0	0	0
a_71_N__a_79_O	246	246	245	246	4	2	0	4
a_71_N__a_81_O	0	0	0	0	3	0	3	6
a_72_NE2__a_51_OE1	0	3	1	0	0	0	0	0

Table S1 C

a_72_NE2__a_59_SD	3	4	9	5	0	0	0	0
a_72_NE2__a_60_O	6	18	21	19	8	8	8	8
a_72_NE2__a_61_OD1	14	14	9	14	13	9	1	11
a_72_NE2__a_62_O	0	0	0	0	6	2	2	2
a_72_NE2__a_70_O	0	0	0	0	3	7	3	5
a_72_NE2__a_72_O	3	2	0	4	0	2	0	1
a_72_NE2__a_73_O	0	0	10	0	1	1	3	5
a_72_NE2__a_74_O	0	1	0	1	0	0	0	0
a_72_NE2__a_75_O	74	6	4	48	0	0	0	3
a_72_NE2__a_76_O	7	3	13	6	39	30	38	20
a_72_NE2__a_77_O	14	1	1	8	2	0	2	3
a_72_NE2__a_78_OG1	15	1	11	2	2	0	1	4
a_72_N__a_61_OD1	169	167	179	157	1	0	0	3
a_72_N__a_62_O	0	0	0	0	8	1	0	4
a_72_N__a_70_O	0	0	0	0	0	0	0	2
a_72_N__a_72_OE1	52	13	74	29	2	19	4	3
a_73_N__a_59_SD	0	4	0	15	0	0	0	0
a_73_N__a_61_OD1	155	63	166	149	0	0	0	0
a_73_N__a_71_O	18	36	83	63	0	0	1	3
a_73_N__a_72_OE1	0	0	6	2	1	3	3	9
a_73_N__a_73_O	36	11	10	22	16	17	19	26
a_73_N__a_73_OG1	0	0	0	3	248	248	248	248
a_73_N__a_76_O	0	0	0	0	0	1	1	1
a_73_N__a_77_O	0	0	0	0	248	248	248	248
**a_73_OG1__a_202_N25**	**0**	**0**	**0**	**0**	**3**	**0**	**0**	**0**
**a_73_OG1__a_202_OT1**	**0**	**0**	**0**	**0**	**40**	**32**	**45**	**37**
**a_73_OG1__a_202_OT2**	**0**	**0**	**0**	**0**	**46**	**31**	**41**	**29**
a_73_OG1__a_71_O	1	1	0	4	0	0	0	0
a_73_OG1__a_72_O	248	248	240	244	0	0	0	0
a_73_OG1__a_72_OE1	0	0	0	0	0	0	0	1
a_73_OG1__a_73_O	0	0	0	2	34	43	42	43
a_73_OG1__a_74_O	27	4	10	0	0	0	0	2
a_73_OG1__a_75_O	0	0	0	22	0	0	0	1
a_73_OG1__a_76_O	0	0	0	0	0	1	0	0
a_73_OG1__a_77_O	38	201	183	66	164	194	187	173
a_73_OG1__a_78_O	0	0	0	0	1	0	0	0
a_73_OG1__a_97_OH	0	0	0	1	0	0	0	0
**a_74_N__a_202_N25**	**0**	**0**	**0**	**0**	**5**	**6**	**4**	**3**
**a_74_N__a_202_O24**	**0**	**0**	**0**	**0**	**68**	**128**	**134**	**121**
**a_74_N__a_202_OT1**	**0**	**0**	**0**	**0**	**22**	**31**	**14**	**27**
**a_74_N__a_202_OT2**	**0**	**0**	**0**	**0**	**34**	**24**	**12**	**26**
a_74_N__a_72_O	123	18	30	91	0	0	0	0
a_74_N__a_73_OG1	195	126	139	151	0	0	0	0
a_74_N__a_74_SD	0	0	0	3	4	18	7	17
a_74_N__a_75_O	0	0	0	24	0	0	0	0
a_74_N__a_97_OH	0	3	2	10	0	0	0	0
**a_75_N__a_202_O24**	**0**	**0**	**0**	**0**	**2**	**10**	**1**	**2**
**a_75_N__a_202_OT1**	**0**	**0**	**0**	**0**	**27**	**22**	**27**	**19**
**a_75_N__a_202_OT2**	**0**	**0**	**0**	**0**	**10**	**22**	**19**	**18**

Table S1 C

a_75_N__a_53_OH	1	2	42	0	0	0	0	0
a_75_N__a_59_SD	18	9	17	8	0	0	0	0
a_75_N__a_72_O	0	0	0	169	0	0	0	0
a_75_N__a_72_OE1	0	0	0	5	0	0	0	0
a_75_N__a_73_O	8	0	8	1	8	8	7	12
a_75_N__a_73_OG1	0	0	0	23	139	133	135	139
a_75_N__a_74_SD	3	3	7	1	0	2	1	0
a_75_N__a_75_O	183	26	62	131	0	0	0	5
a_76_N__a_72_O	2	6	51	10	0	0	0	0
a_76_N__a_72_OE1	0	0	0	6	0	0	0	0
a_76_N__a_73_O	0	0	0	0	201	208	223	173
a_76_N__a_73_OG1	0	0	0	1	89	71	74	90
a_76_N__a_74_O	0	8	7	0	0	0	0	0
**a_77_NZ__a_202_N25**	**0**	**0**	**0**	**0**	**2**	**0**	**0**	**0**
**a_77_NZ__a_202_O24**	**0**	**0**	**0**	**0**	**2**	**0**	**0**	**1**
**a_77_NZ__a_202_OT1**	**0**	**0**	**0**	**0**	**5**	**1**	**8**	**6**
**a_77_NZ__a_202_OT2**	**0**	**0**	**0**	**0**	**4**	**4**	**7**	**10**
a_77_NZ__a_21_O	0	6	46	7	0	0	0	0
a_77_NZ__a_22_O	9	1	5	2	0	0	0	0
a_77_NZ__a_26_OD2	0	0	1	0	0	0	0	0
a_77_NZ__a_73_OG1	0	0	0	0	0	1	0	0
a_77_NZ__a_75_O	0	0	0	1	8	1	4	6
a_77_NZ__a_78_O	6	3	0	1	0	1	0	2
**a_77_N__a_202_OT1**	**0**	**0**	**0**	**0**	**3**	**0**	**0**	**2**
a_77_N__a_72_O	8	13	45	10	0	0	0	0
a_77_N__a_73_OG1	0	0	0	0	160	130	136	150
a_77_N__a_75_O	10	48	18	65	115	147	130	102
a_78_N__a_72_OE1	0	0	0	2	0	0	0	0
a_78_N__a_76_O	10	1	12	13	0	0	0	0
a_78_OG1__a_69_O	0	0	0	0	0	1	4	5
a_78_OG1__a_69_OG	0	0	0	0	1	0	0	3
a_78_OG1__a_70_OD1	10	5	3	8	0	0	0	0
a_78_OG1__a_71_O	0	0	9	0	1	0	12	0
a_78_OG1__a_72_OE1	9	2	12	21	10	4	6	5
a_78_OG1__a_76_O	3	4	7	4	1	0	0	1
a_78_OG1__a_77_O	0	0	0	29	0	0	0	0
a_78_OG1__a_78_O	33	15	48	29	3	5	10	8
a_79_N__a_70_OD1	1	0	5	0	0	0	0	0
a_79_N__a_71_O	248	247	240	247	178	205	191	204
a_79_N__a_77_O	0	0	2	0	0	0	0	0
a_79_N__a_78_OG1	0	0	2	0	0	0	13	0
a_7_N__a_123_OG	0	0	0	0	0	4	5	7
a_7_N__a_124_O	248	248	248	248	231	241	241	208
a_80_NZ__a_68_O	0	0	0	0	0	0	1	0
a_80_NZ__a_68_OE1	25	52	53	25	67	37	48	46
a_80_NZ__a_68_OE2	18	32	36	18	62	37	48	54
a_80_NZ__a_69_OG	0	0	0	0	41	45	47	80
a_80_NZ__a_70_OD1	0	2	10	9	0	0	0	0
a_80_NZ__a_78_O	0	0	0	0	0	1	0	0

Table S1 C

a_80_NZ__a_78_OG1	0	0	0	1	3	10	6	15
**a_80_N__a_202_OT1**	**0**	**0**	**0**	**0**	**1**	**0**	**0**	**0**
a_80_N__a_78_O	0	0	0	0	0	0	0	2
a_81_N__a_68_OE1	0	0	0	0	1	2	2	1
a_81_N__a_68_OE2	0	0	0	0	0	1	0	0
a_81_N__a_69_O	248	247	247	247	0	0	0	0
a_81_N__a_69_OG	0	0	0	0	1	3	0	0
a_81_N__a_79_O	47	19	57	104	236	215	240	235
a_81_N__a_81_O	245	248	225	226	229	190	220	210
a_82_N__a_93_O	106	34	70	106	63	17	52	15
a_82_N__a_93_OD1	1	0	22	16	0	0	0	0
a_82_OG1__a_66_O	0	0	0	0	1	0	0	0
a_82_OG1__a_67_O	3	2	1	0	20	14	4	15
a_82_OG1__a_68_OE1	0	0	0	0	2	11	6	2
a_82_OG1__a_68_OE2	0	1	0	0	4	4	7	0
a_82_OG1__a_80_O	0	0	0	0	0	3	0	0
a_82_OG1__a_81_O	2	4	1	2	7	4	2	1
a_82_OG1__a_82_O	97	67	99	126	17	13	30	46
a_82_OG1__a_83_O	0	0	0	0	2	0	0	0
a_82_OG1__a_84_OE1	17	9	20	12	12	27	51	61
a_82_OG1__a_93_O	10	1	12	8	7	3	7	5
a_82_OG1__a_93_OD1	53	10	58	63	0	0	1	11
a_83_N__a_66_O	0	0	0	1	0	0	0	0
a_83_N__a_67_O	121	87	102	97	15	8	37	4
a_83_N__a_81_O	0	1	2	6	0	2	0	4
a_83_N__a_82_OG1	0	0	0	0	17	0	0	0
a_84_NE2__a_66_O	0	1	0	0	0	0	0	0
a_84_NE2__a_82_O	0	1	1	2	8	8	8	0
a_84_NE2__a_82_OG1	0	0	1	0	6	1	8	13
a_84_NE2__a_83_O	0	0	1	3	0	0	0	0
a_84_NE2__a_84_O	0	0	0	1	0	0	0	0
a_84_NE2__a_85_O	3	1	3	3	3	0	4	1
a_84_NE2__a_86_OE1	5	6	4	3	4	1	0	6
a_84_NE2__a_86_OE2	2	7	4	4	2	3	1	5
a_84_NE2__a_91_O	42	24	58	46	28	64	61	59
a_84_N__a_82_O	0	0	0	0	0	0	1	0
a_84_N__a_84_OE1	0	0	0	0	2	2	2	2
a_84_N__a_91_O	247	245	247	248	239	235	206	232
a_85_N__a_83_O	0	1	0	1	0	0	0	0
a_85_N__a_84_OE1	17	11	14	12	16	2	8	11
a_86_N__a_84_O	0	0	0	0	0	1	0	2
a_86_N__a_85_SD	10	11	10	7	3	0	1	3
a_86_N__a_86_O	98	24	64	43	146	164	142	140
a_86_N__a_86_OE1	0	2	0	0	0	0	0	0
a_86_N__a_89_O	248	226	235	243	247	245	248	243
a_87_N__a_85_O	8	1	2	6	2	11	4	9
a_87_N__a_85_SD	12	39	26	28	3	1	3	3
a_87_N__a_86_OE1	4	7	2	3	0	1	0	0
a_87_N__a_86_OE2	6	1	1	2	0	0	0	0

Table S1 C

a_87_N__a_89_O	2	39	17	46	0	0	3	0
a_88_N__a_85_O	0	0	1	0	0	0	0	3
a_88_N__a_85_SD	19	1	7	0	22	16	2	16
a_88_N__a_86_O	110	79	104	95	37	84	88	53
a_88_N__a_86_OE1	0	17	0	2	0	0	0	0
a_88_N__a_86_OE2	0	0	0	2	0	0	1	0
a_88_N__a_89_O	0	0	1	0	1	5	0	3
a_89_NZ__a_100_OG1	0	0	0	0	0	1	0	2
a_89_NZ__a_101_O	0	0	0	0	17	12	65	18
a_89_NZ__a_102_OE1	20	27	21	29	57	89	44	93
a_89_NZ__a_102_OE2	14	32	19	24	55	87	68	92
a_89_NZ__a_86_O	15	0	8	2	21	17	15	15
a_89_NZ__a_86_OE1	0	1	5	6	1	10	4	2
a_89_NZ__a_86_OE2	6	10	19	12	4	2	3	1
a_89_N__a_85_SD	0	1	1	2	0	0	0	0
a_89_N__a_86_O	79	22	68	45	60	82	96	64
a_89_N__a_86_OE1	0	13	0	1	0	0	0	0
a_89_N__a_86_OE2	0	0	0	2	0	0	1	0
a_89_N__a_87_O	0	0	1	0	0	2	0	2
a_8_N__a_36_O	119	217	222	223	132	115	182	128
a_8_N__a_7_OE1	15	0	0	5	23	5	0	1
a_8_N__a_7_OE2	3	0	0	3	14	8	0	0
a_8_N__a_8_SD	1	2	1	8	0	0	1	0
a_90_N__a_101_O	247	247	247	241	66	23	67	38
a_90_N__a_101_OG	0	0	0	3	37	129	32	104
**a_90_N__a_201_O**	**0**	**0**	**0**	**0**	**39**	**22**	**56**	**32**
a_91_N__a_84_O	247	247	247	247	247	244	237	244
a_92_N__a_99_O	247	247	245	244	214	211	186	187
a_92_N__a_99_OE1	0	0	3	0	0	1	35	20
a_93_ND2__a_82_O	0	0	8	0	0	0	0	0
a_93_ND2__a_82_OG1	12	4	11	19	4	1	1	2
a_93_ND2__a_84_OE1	0	0	0	1	12	11	12	15
a_93_ND2__a_92_O	0	0	0	0	0	0	1	0
a_93_ND2__a_93_O	1	4	1	1	9	3	1	4
a_93_ND2__a_94_O	0	1	0	2	1	0	0	0
a_93_ND2__a_97_O	0	0	0	0	2	0	0	0
a_93_ND2__a_98_ND1	41	78	43	23	16	16	20	19
a_93_ND2__a_98_NE2	0	1	0	0	0	1	0	1
a_93_N__a_82_O	124	46	93	152	80	24	60	29
a_93_N__a_82_OG1	0	0	0	0	0	0	1	0
a_93_N__a_91_O	0	0	0	0	1	0	0	0
a_93_N__a_93_OD1	64	55	89	66	0	0	0	6
a_94_N__a_92_O	1	2	0	3	0	1	0	3
a_94_N__a_93_OD1	0	0	1	1	0	1	3	7
a_94_N__a_97_O	245	237	239	240	188	233	204	221
a_96_ND2__a_114_O	0	0	0	0	2	1	7	6
**a_96_ND2__a_202_N25**	**0**	**0**	**0**	**0**	**0**	**1**	**0**	**0**
**a_96_ND2__a_202_O24**	**0**	**0**	**0**	**0**	**0**	**1**	**0**	**1**
a_96_ND2__a_20_O	16	1	38	8	0	4	0	0

Table S1 C

a_96_ND2__a_21_O	119	9	16	22	0	0	0	0
a_96_ND2__a_73_OG1	22	30	5	4	0	0	0	0
a_96_ND2__a_74_O	0	14	8	0	0	0	0	0
a_96_ND2__a_74_SD	0	0	0	7	0	0	0	0
a_96_ND2__a_95_O	0	0	0	0	3	1	2	0
a_96_ND2__a_96_O	2	1	7	9	36	30	31	18
a_96_N__a_94_O	123	124	72	71	26	12	32	37
a_96_N__a_96_OD1	82	132	73	92	0	3	0	16
a_96_N__a_98_NE2	0	0	0	0	0	0	14	0
a_97_N__a_94_O	106	78	109	113	199	206	215	177
a_97_N__a_95_O	0	0	0	0	1	0	3	0
a_97_N__a_96_OD1	50	96	33	22	0	0	0	0
a_97_OH__a_112_OG	0	0	10	0	2	9	7	6
a_97_OH__a_119_OH	0	0	0	0	0	0	0	16
**a_97_OH__a_201_O2**	**0**	**0**	**0**	**0**	**0**	**5**	**0**	**0**
**a_97_OH__a_202_O7**	**0**	**0**	**0**	**0**	**13**	**4**	**0**	**6**
a_97_OH__a_21_O	1	0	0	1	0	0	0	0
a_97_OH__a_73_O	1	0	0	0	0	0	0	0
a_97_OH__a_73_OG1	13	8	1	7	0	0	0	0
a_97_OH__a_74_SD	0	1	4	2	0	0	0	0
a_97_OH__a_99_OE1	12	11	17	21	197	151	147	131
a_98_ND1__a_113_O	0	0	0	0	0	1	1	1
a_98_ND1__a_113_OG1	0	0	0	0	5	0	5	0
a_98_ND1__a_92_O	0	0	0	0	141	192	149	148
a_98_ND1__a_93_OD1	0	0	0	0	70	39	43	25
a_98_ND1__a_94_O	0	0	0	0	0	5	1	10
a_98_ND1__a_96_O	0	0	0	0	0	0	0	4
a_98_ND1__a_97_O	0	0	0	0	0	4	2	5
a_98_ND1__a_98_O	0	0	0	0	0	1	0	0
a_98_ND1__a_99_O	0	0	0	0	0	2	1	3
a_98_NE2__a_100_OG1	7	6	6	7	0	0	0	0
a_98_NE2__a_93_OD1	1	0	0	0	0	0	0	0
a_98_N__a_113_O	245	243	240	244	233	217	234	226
a_98_N__a_113_OG1	0	0	0	1	0	1	0	1
a_98_N__a_96_O	0	0	0	0	0	0	0	1
a_99_NE2__a_100_O	1	0	0	0	1	0	0	2
a_99_NE2__a_101_OG	1	1	7	6	26	15	12	11
a_99_NE2__a_110_OE1	21	32	27	15	93	85	142	86
a_99_NE2__a_110_OE2	25	11	25	17	122	80	71	103
a_99_NE2__a_111_O	2	11	0	1	0	0	0	0
a_99_NE2__a_112_OG	52	62	46	51	0	2	1	2
**a_99_NE2__a_201_O**	**0**	**0**	**0**	**0**	**0**	**1**	**7**	**8**
**a_99_NE2__a_201_O1**	**0**	**0**	**0**	**0**	**0**	**0**	**1**	**3**
**a_99_NE2__a_201_O3**	**0**	**0**	**0**	**0**	**2**	**0**	**1**	**0**
**a_99_NE2__a_202_O3**	**0**	**0**	**0**	**0**	**3**	**3**	**3**	**2**
**a_99_NE2__a_202_O7**	**0**	**0**	**0**	**0**	**1**	**1**	**0**	**0**
a_99_NE2__a_90_O	0	0	0	0	0	19	0	2
a_99_NE2__a_97_OH	2	0	4	2	0	2	3	7
a_99_NE2__a_98_O	0	0	0	0	0	1	0	0

Table S1 C

a_99_NE2__a_99_O	0	2	1	1	0	0	0	0
a_99_N__a_92_O	248	247	245	245	178	213	179	194
a_99_N__a_98_ND1	4	6	11	8	18	29	42	24
a_99_N__a_99_OE1	0	0	0	0	0	0	1	0
a_9_N__a_10_O	0	0	0	1	21	1	0	3
a_9_N__a_11_OE1	0	0	0	0	0	0	1	1
a_9_N__a_11_OE2	0	0	0	0	0	1	0	0
a_9_N__a_122_O	246	248	242	228	248	246	236	234
a_9_N__a_123_OG	0	0	0	1	1	0	0	0
a_9_N__a_7_O	19	41	37	28	4	5	5	12
a_9_N__a_8_SD	3	7	4	5	4	5	7	10
a_9_N__a_9_O	0	0	0	0	188	64	233	113
a_9_N__a_9_OE2	0	0	0	2	0	0	0	0

Table S1 C
