# Supplementary material for: Different modes of barrel opening suggest a complex pathway of ligand binding in human gastrotropin
Source: PLoS One. 2019 May 10;14(5):e0216142. doi: 10.1371/journal.pone.0216142 (PMC6510414; doi:10.1371/journal.pone.0216142)
Supplement: S1 Appendix — (DOCX) [file pone.0216142.s009.docx]

Main features of protein-ligand interactions are similar in the simulated and docked structures

The orientation of the ligands and amino acids being in contact with the ligands were determined by comparing distances between ligand heavy atoms and amino acid heavy atoms. An amino acid is counted being in contact with a certain heavy atom of the ligand if there is at least one heavy atom in the amino acid, which is closer than 4 Å from the selected heavy atom of the ligand. For each simulation frame, starting from the *holo* structure, a Tanimoto distance was determined based on these data, depicted in (Fig. S2 A).

The distance data plotted in (Fig S2 A) reveals, that there are many ligand orientations, which are similar to each other, but a certain group of frames have a large distance with any other frames. These are some frames from 2500 to 2900, from a 20 ns long simulation on 298 K without any restraints. Also the docked conformers, coming after 3500 are very different from the simulation frames, indicating different ligand orientations.

Depicting for each amino acids the average of the number of ligand heavy atoms being close than 4 Å (Fig S2 B-C), it is visible, that in the extent of amino acid contacts, the docked structures are not significantly different from all the other structures. This implicates different ligand orientations involving the same amino acids.
